# Unexpected Vulnerability of DPEphos to C−O Activation in the Presence of Nucleophilic Metal Hydrides

**DOI:** 10.1002/chem.202001685

**Published:** 2020-07-28

**Authors:** Mateusz K. Cybulski, Nicholas A. Beattie, Stuart A. Macgregor, Mary F. Mahon, Michael K. Whittlesey

**Affiliations:** ^1^ Department of Chemistry University of Bath Claverton Down Bath BA2 7AY UK; ^2^ Institute of Chemical Sciences Heriot-Watt University Edinburgh EH14 4AS UK

**Keywords:** C−O bond activation, density functional calculations, hydride ligands, N-heterocyclic carbenes, phosphines

## Abstract

C−O bond activation of DPEphos occurs upon mild heating in the presence of [Ru(NHC)_2_(PPh_3_)_2_H_2_] (NHC=N‐heterocyclic carbene) to form phosphinophenolate products. When NHC=IEt_2_Me_2_, C−O activation is accompanied by C−N activation of an NHC ligand to yield a coordinated *N*‐phosphino‐functionalised carbene. DFT calculations define a nucleophilic mechanism in which a hydride ligand attacks the aryl carbon of the DPEphos C−O bond. This is promoted by the strongly donating NHC ligands which render a *trans* dihydride intermediate featuring highly nucleophilic hydride ligands accessible. C−O bond activation also occurs upon heating *cis*‐[Ru(DPEphos)_2_H_2_]. DFT calculations suggest this reaction is promoted by the steric encumbrance associated with two bulky DPEphos ligands. Our observations that facile degradation of the DPEphos ligand via C−O bond activation is possible under relatively mild reaction conditions has potential ramifications for the use of this ligand in high‐temperature catalysis.

## Graphical abstract

**Hydride ligands**: Under mild conditions (25→90 °C), [Ru(N‐heterocyclic carbene)_2_(PPh_3_)_2_H_2_]/DPEphos or [Ru(DPEphos)_2_H_2_] undergo C−O bond activation of this catalytically important diphosphine ligand by attack of the nucleophilic Ru−H ligands.

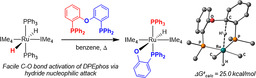

Since their introduction ca. 20 years ago,^1^ wide‐angle phosphines such as xantphos and DPEphos (Scheme 1) have become indispensable ligands for a range of catalytic reactions.^2^ Their usage stems from two advantageous properties; firstly, the availability of highly flexible bite angles that allow *cis*‐ and *trans*‐, as well as hemilabile P‐O‐P coordination modes, to be adopted^3^ and, secondly, resistance to the types of P−C degradation reactions reported in tertiary phosphine metal complexes.^4^ This latter property has promoted the use of xantphos and DPEphos in reactions that require high temperatures.2c, 2g, 2l, 5

**Scheme 1:**
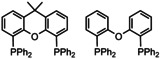
Structures of xantphos and DPEphos.

Any suggestion that such phosphines might be susceptible to degradative reactions, particularly under relatively mild conditions, could therefore have important ramifications for their applications in catalysis. While xantphos has been reported to be susceptible to P−C bond activation at room temperature,^6^ cleavage of DPEphos appears to be restricted to a single example of high temperature C−O bond activation reported by Weller and Willis.^7^ In the course of studies on [Rh(η^6^‐*ortho*‐xylene)(DPEphos)]^+^ catalysed carbothiolation of alkynes, they reported that heating the Rh complex together with *ortho*‐MeSC_6_H_4_C(O)Me at 120 °C in the absence of any alkyne led to C−O cleavage of DPEphos to afford a catalytically inactive Rh complex with chelating phosphine aryloxide and bidentate phosphine arylthioether ligands. Herein, we demonstrate that C−O activation of DPEphos can take place even at room temperature in the presence of ruthenium dihydride complexes. DFT calculations reveal that such processes involve attack of highly nucleophilic hydride ligands on the aryl carbon on the C−O bond.

In the course of studies to investigate the substitution chemistry of the all *trans*‐dihydride complex [Ru(IMe_4_)_2_(PPh_3_)_2_H_2_] (**1**, Scheme 2),^8^ **1** was treated with 1.1–1.5 equiv DPEphos in benzene. No immediate reaction was observed at room temperature, but upon heating to 90 °C for ca. 12 h, a single ruthenium‐containing product **2** (Scheme 2) was formed. An X‐ray crystal structure (Figure 1) revealed the presence of a phosphinophenolate ligand generated upon C−O activation of DPEphos.^9^ The P,O‐termini of the ligand were *trans* to PPh_3_ and Ru−H respectively. The coordination sphere was completed by two mutually *trans* IMe_4_ ligands, each of which displayed an *N*‐Me group with a short C−H⋅⋅⋅O contact to the phosphinophenolate ligand (Supporting Information). The *trans* H‐Ru‐O arrangement led to both a long Ru−O distance (2.2720(16) Å)^10^ and a low frequency (*δ*=−18.40 ppm) hydride resonance.^11^

**Scheme 2:**
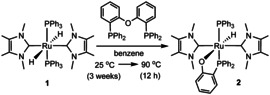
C−O activation of DPEphos by **1** to give **2**.

**Figure 1:**
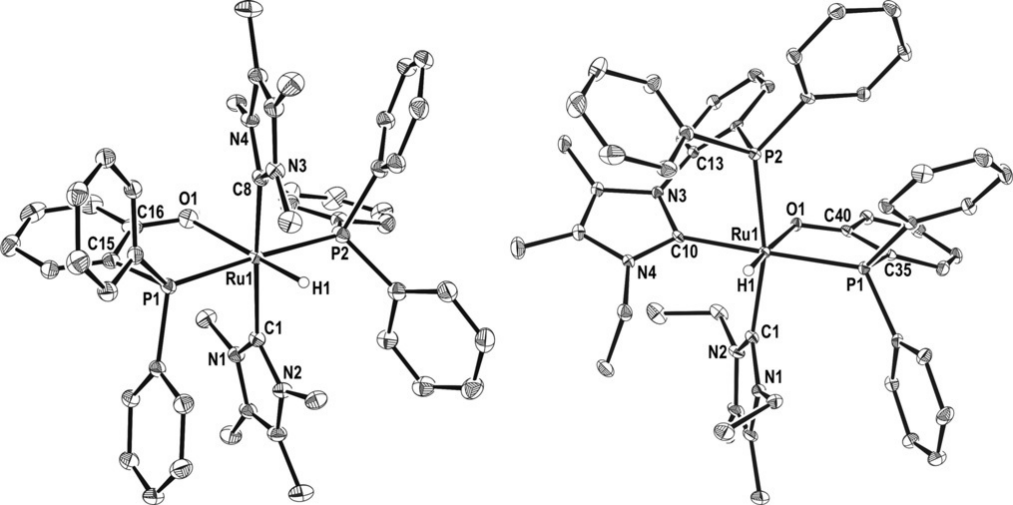
Molecular structures of (left) **2** and (right) **4**. Thermal ellipsoids are shown at 30 % level. All hydrogen atoms, except for Ru−H have been omitted for clarity.

The formation of **2** was achieved under even milder conditions, although at the expense of longer reaction times (e.g. 6 days at 70 °C), and a low (5 %) yield could even be formed at room temperature, albeit only over 3 weeks.^12^ No simple substitution product arising from replacement of the two PPh_3_ ligands by DPEphos was observed under these conditions (vide infra). Treatment of **1** with the more‐electron rich cyclohexyl diphosphine Cy_2_P(C_6_H_4_)O(C_6_H_4_)PCy_2_ also resulted in C–O activation, although the reaction failed to reach completion, even after heating at 120 °C for 2 days. There was no evidence for C−O activation of xantphos by **1**.^13^

Replacing **1** by the *N*‐Et substituted carbene derivative *cis*, *cis*, *trans*‐[Ru(IEt_2_Me_2_)_2_(PPh_3_)_2_H_2_] (**3**, Scheme 3) led to an even more unexpected reaction with DPEphos. Heating in toluene at 90 °C gave the phosphinophenolate complex **4** (Scheme 3), in which the {Ph_2_P(C_6_H_4_)} moiety generated upon C−O cleavage had combined with a C−N activated IEt_2_Me_2_ ligand to generate a Ru‐bound *N*‐phosphino‐functionalised carbene ligand.^14^ The X‐ray structure of **4** (Figure 1) showed the presence of a distorted octahedral ruthenium centre with a *cis*‐arrangement of the two carbenes and two phosphines and the same *trans* H‐Ru‐OAr arrangement as in **1** (Ru−O=2.265(2) Å). The phosphino moiety appended to N3 exhibited a considerable cone‐tilt, with Ru‐P‐C_ipso_ angles ranging from 102° to 132°. In support of the C−N cleavage process, the ^1^H NMR spectrum showed just three NCH_2_CH_3_ methyl and six NCH_2_CH_3_ methylene resonances. The Ru−H resonance (*δ*=−17.7 ppm) was coupled to the two inequivalent phosphorus nuclei (*δ*=59 and 55 ppm) with *cis*‐^2^ *J*(H,P) coupling constants of 20 and 15 Hz.

**Scheme 3:**
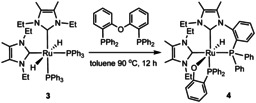
C−O and C−N activation to yield **4**.

C−N activation of a metal‐bound NHC ligand has been described previously,^15^ including in studies on Ru‐NHC complexes related to those employed here.^16^ However, this process has only rarely been observed alongside the activation of another ligand,^17^ and, certainly not as a route to the formation of a phosphinocarbene.^18^

The C−O activation of DPEphos was not restricted to NHC‐containing ruthenium hydride precursors. The reaction of [Ru(PPh_3_)_4_H_2_] with DPEphos gave the isolable *cis*‐dihydride complex [Ru(DPEphos)_2_H_2_] (**5**; Supporting Information),^19^ which upon heating to 80 °C overnight underwent C−O activation of one of the DPEphos ligands to afford [Ru(DPEphos)(Ph_2_PC_6_H_4_O)H] (**6**, Scheme 4).^20^ This was characterized by the presence of a quartet Ru−H resonance at *δ*=−14 ppm with a ^2^ *J*(H,P) splitting (22 Hz) indicative of hydride *cis* to all three phosphorus nuclei and a ^31^P{^1^H} NMR spectrum which showed a triplet at *δ*=77 ppm (^2^ *J*(P,P)=30 Hz), together with a broad, featureless signal at *δ*=50 ppm. We attribute the latter to the intact DPEphos ligand switching rapidly between *κ* ^2^‐P,P and *κ* ^3^‐P,O,P coordination. At −15 °C, this signal resolved into two doublets, the two ends of the DPEphos ligand becoming inequivalent as a result of the oxygen now staying bound to Ru. Although an X‐ray structure of **6** proved elusive, crystals of the chloride derivative **7** were isolated from CH_2_Cl_2_/pentane solutions of **6**, affording a structure (Figure 2) which confirmed the coordination modes at ruthenium.

**Scheme 4:**
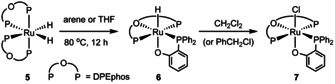
Formation of the C−O activated DPEphos complex **6** and chloride derivative **7**.

**Figure 2:**
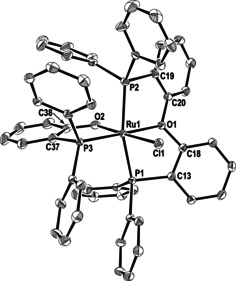
Molecular structure of **7**. Thermal ellipsoids are shown at 30 % probability. Cl1 is disordered with a hydride ligand in a 75:25 ratio. Hydrogen atoms and all minor disordered components have been for clarity.

DFT calculations^21^ have been used to explore the mechanism of the C−O bond cleavage reactions in **1** and **5** and the factors promoting them. For **1**, no intermediates are observed experimentally and so all free energies are quoted relative to this species plus free DPEphos. PPh_3_ substitution in **1** by DPEphos gives [Ru(IMe_4_)_2_(DPEphos)H_2_], **8**, for which the all‐*cis* isomer, **8_ccc_** (+3.6 kcal mol^−1^), and the *cis*, *cis*, *trans*‐isomer, **8_cct_** (+4.2 kcal mol^−1^) are most stable.^22^

The accessibility of the *trans* dihydride isomer **8_cct_** suggested a hydride nucleophilic attack mechanism may be involved, similar to that characterised for the hydrodefluorination of (hetero)aromatics at *trans*‐[Ru(NHC)_4_H_2_] complexes.^23^, ^24^ Figure 3 shows the computed reaction profiles for this process in **8_cct_** and **8_ccc_**. For **8_cct_** the *trans* hydride arrangement gives a long Ru−H^1^ bond (1.70 Å) and NBO calculations indicate significant hydridic character (−0.21). Nucleophilic attack proceeds via **TS(8‐2)_cct_** at +25.0 kcal mol^−1^, with a short H^1^⋅⋅⋅C^1^ distance of 1.56 Å and Ru⋅⋅⋅H^1^ stretching to 1.84 Å. The C^1^−O bond also lengthens to 1.48 Å and elongated C^1^−C^2^ and C^1^−C^6^ distances in the aryl ring suggest a Meisenheimer‐type structure consistent with nucleophilic aromatic substitution. Hydride attack is also accompanied by a conformational change in the 8‐membered Ru−P−C=C−O−C=C−P ring, from a distorted twist‐boat conformation in **8_cct_** to a boat conformation in the transition state,^25^ similar to the DPEphos *fac*‐*κ* ^3^‐P,O,P binding mode.^26^ IRC calculations confirm that **TS(8‐2)_cct_** links directly to **2_cct_** in which H^2^ is *trans* to the phosphinophenolate oxygen. The lowest energy conformation of **2_cct_** is at −31.5 kcal mol^−1^.^27^

**Figure 3:**
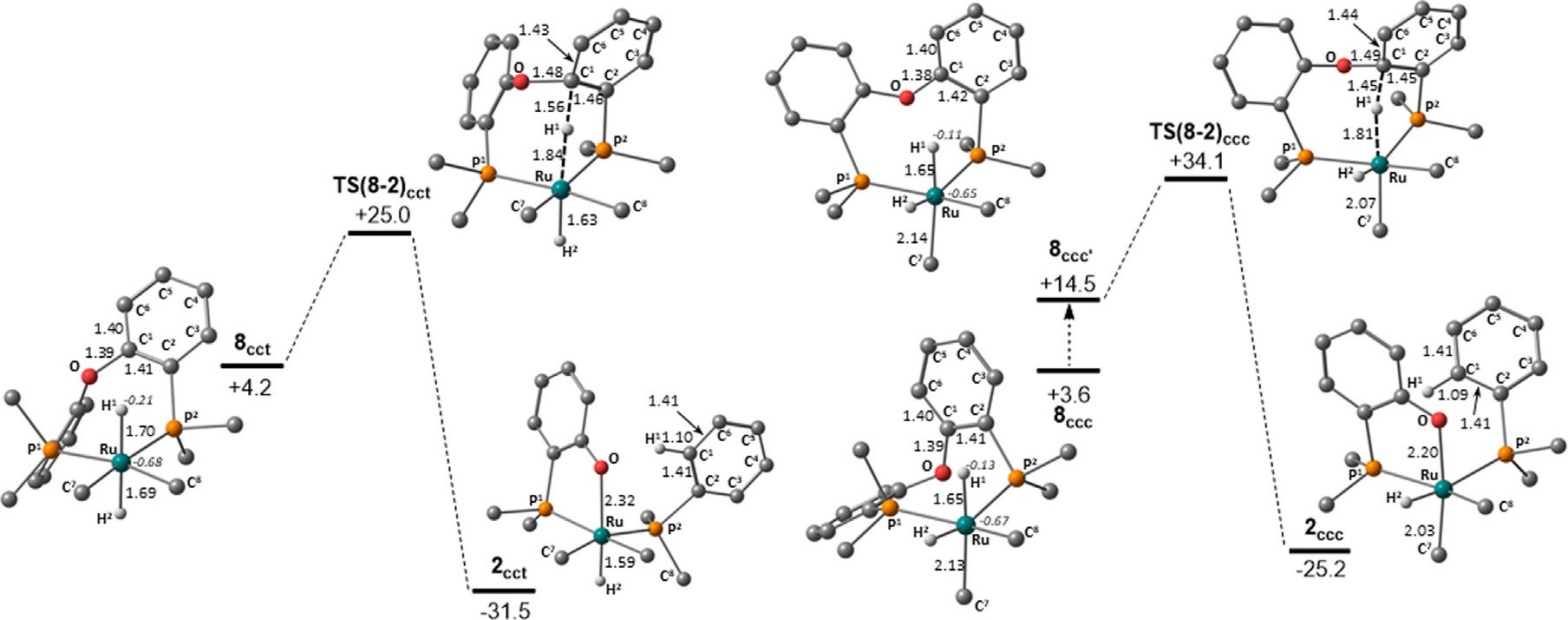
Computed free energy profiles (kcal mol^−1^, BP86(benzene, D3BJ)) for hydride attack in **8_cct_** and **8_ccc_**, with selected distances in Å. Energies are relative to **1** plus free DPEphos and NBO charges at Ru and H^1^ are indicated in italics for dihydride precursors. For clarity, IMe_4_ ligands are truncated at the C2 position (i.e. C^7^ and C^8^ in the Figure) and phenyl substituents at the *ipso* carbons. DPEphos hydrogens are also omitted. **8_ccc’_** is a conformer of **8_ccc_** that lies directly on the pathway for C−O cleavage (see text for details).

The equivalent reaction of **8_ccc_** involves an initial conformational change of the Ru−P−C=C−O−C=C−P ring to form **8_ccc’_** at +14.5 kcal mol^−1^. C−O bond cleavage then proceeds via **TS(8‐2)_ccc_** at +34.1 kcal mol^−1^ with similar geometric changes to those described above for **TS(8‐2)_cct_**. The shorter Ru‐H^1^ distances in **8_ccc_** and **8_ccc’_** (1.65 Å) and lower NBO charges (ca. −0.12) indicate that H^1^ is now less nucleophilic than in **8_cct_**, and this reflects the change in the *trans* ligand, from a hydride in **8_cct_** to IMe_4_ in **8_ccc_**. This also correlates with C−O bond cleavage being less kinetically accessible in **8_ccc_**. **TS(8‐2)_ccc_** leads to **2_ccc_** at −25.2 kcal mol^−1^, substantially less stable than **2_cct_** as this structure lacks the favourable *trans*‐H‐Ru‐O arrangement.^28^

C−O bond cleavage was also modelled for [Ru(DPEphos)_2_H_2_] and the most accessible pathway is shown in Figure 4. The all‐*cis* isomer, **5_ccc_**, reacts via **5_ccc’_** and **TS(5**–**9)_ccc_** at +29.9 kcal mol^−1^ to give a phosphinophenolate product, **9_ccc_**, at −23.0 kcal mol^−1^. The short Ru−H^1^ distance in **5_ccc_** (1.60 Å) and low NBO charge on H^1^ (−0.02) indicate reduced hydride nucleophilicity compared to **8_ccc_**, although the barrier in the bis‐DPEphos system is actually lower (see below). In stark contrast to **8_cct_**, the *trans* dihydride isomer of [Ru(DPEphos)_2_H_2_] **5_cct_**, has a large barrier of +48.5 kcal mol^−1^. This difference is due in part to the higher energy of **5_cct_** (+13.8 kcal mol^−1^) and the reduced charge on H^1^ (ca. −0.08 cf. −0.21 in **8_cct_**). The latter result highlights how the NHC ligands also serve to enhance hydride nucleophilicity. Differential steric effects in the transition states may also be a factor, as probed by calculations on **5_ccc_** and **5_cct_** in which the PPh_2_ groups were replaced by PH_2_. This model system gave a similar relative energy for **5_cct_** (+12.6 kcal mol^−1^), but a reduced barrier for the subsequent nucleophilic attack (i.e. from **5_cct_** to **TS(5**–**9)_cct_**: 30.2 kcal mol^−1^ cf. 34.7 kcal mol^−1^ in the full system). In contrast, the computed barrier for **5_ccc_** with the small model is 38.6 kcal mol^−1^, 8.7 kcal mol^−1^ *higher* than the full model.

**Figure 4:**
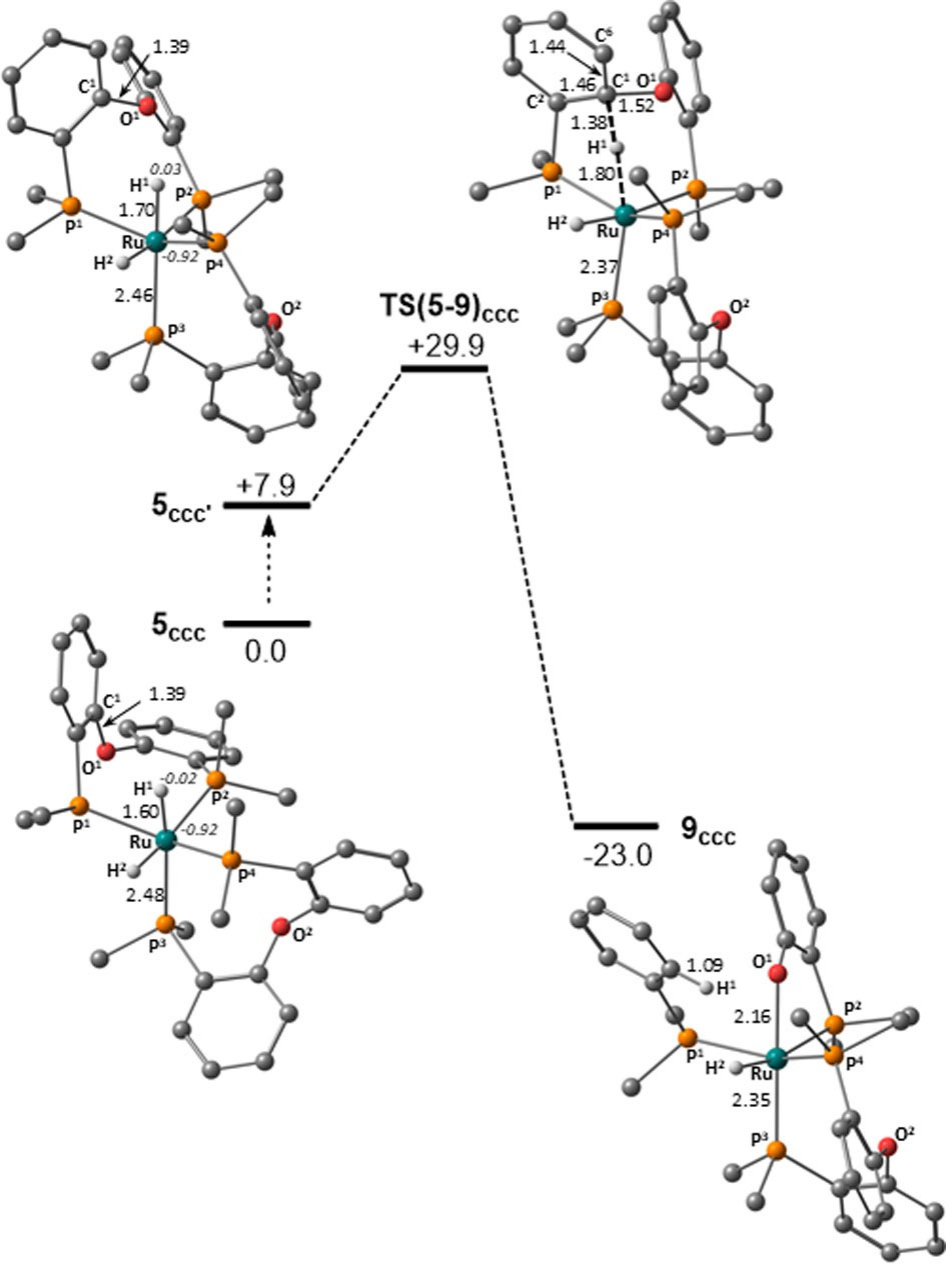
Computed free energy profile (kcal mol^−1^, BP86(benzene, D3BJ)) for hydride attack in **5_ccc_**, with selected distances in Å and computed NBO charges at Ru and H^1^ in italics for the dihydride precursors. For clarity, phenyl substituents are truncated at the *ipso* carbons and DPEphos hydrogens are omitted.

Computed geometries show significant distortions in the full model: in **5_ccc_** the *trans*‐P‐Ru‐P angle is 142° with the bulky PAr_3_ moieties tilting over the hydride ligands. As this angle is only 160° in the small model, we speculate that the greater distortion of the full model enables nucleophilic attack.

Comparing [Ru(IMe_4_)_2_(DPEphos)H_2_] and [Ru(DPEphos)_2_H_2_] shows C−O bond cleavage via **8_cct_** (Δ*G* ^≠^=25.0 kcal mol^−1^) is more accessible than in **5_ccc_** (Δ*G* ^≠^=29.9 kcal mol^−1^) and this is consistent with the lower reactivity of the bis‐DPEphos system observed experimentally. Lower barriers are computed with higher *trans* influence ligands (H>IMe_4_) *trans* to the hydride nucleophile. The mixed NHC/DPEphos systems appear particularly vulnerable to C−O bond cleavage as the strongly donating NHC ligands both enhance hydride nucleophilicity and render **8_cct_**, the key *trans* dihydride precursor, accessible. The hydride attack mechanism described here has similarities to the “asynchronous oxidative addition” pathway described by Crimmin and co‐workers where a Ru^II^ metal centre acts as a nucleophile prior to C−O bond cleavage.^12^ A similar asynchronicity is seen here, with C−H bond formation in the transition state being far advanced of either C−O bond cleavage or Ru−O bond formation.

In summary, we have characterised the surprisingly facile C−O bond activation of DPEphos ligands in the presence of nucleophilic hydrides. Ligand exchange of all‐*trans*‐[Ru(IMe_4_)_2_(PPh_3_)_2_H_2_] with DPEphos results in the formation of phosphinophenolate complex, **2**, while with *cis*, *cis*, *trans*‐[Ru(IEt_2_Me_2_)_2_(PPh_3_)_2_H_2_], C−O bond cleavage is accompanied by C−N activation of the NHC to form the *N*‐phosphino‐functionalised carbene complex **4**. DFT calculations indicate that C−O activation involves a nucleophilic pathway in which a hydride ligand attacks the aryl carbon of the DPEphos C−O bond. This process is promoted by the accessibility of a *trans* dihydride intermediate that features highly nucleophilic hydride ligands. C−O bond activation also occurs upon heating *cis*‐[Ru(DPEphos)_2_H_2_], a process that DFT calculations indicate is promoted by the steric encumbrance of the mutually *cis* DPEphos ligands. This undesirable ligand degradation of DPEphos is of particular note given the wide use of this ligand in high temperature homogeneous catalysis. Indeed, degradation of the Rh‐DPEphos system described by Weller and Willis is also thought to involve nucleophilic attack, in this case by a thiolate ligand.^7^ On a more constructive note, the hydride nucleophilic attack mechanism proposed here has already been shown to operate in catalytic C−F functionalization,23c, 23d and so may also be an effective means of promoting C−O bond activation of the type required for the valorization of lignin and of its highly oxygenated monomers.^29^

## Conflict of interest

The authors declare no conflict of interest.

## Supporting information

As a service to our authors and readers, this journal provides supporting information supplied by the authors. Such materials are peer reviewed and may be re‐organized for online delivery, but are not copy‐edited or typeset. Technical support issues arising from supporting information (other than missing files) should be addressed to the authors.

Supplementary
