# Supplementary material for: Unexpected Vulnerability of DPEphos to C−O Activation in the Presence of Nucleophilic Metal Hydrides
Source: Chemistry. 2020 Jul 28;26(49):11141–5. doi: 10.1002/chem.202001685 (PMC7496192; doi:10.1002/chem.202001685)
Supplement: Supplementary file 1 — Supplementary [file CHEM-26-11141-s001.pdf]

Chemistry–A European Journal

Supporting Information

Unexpected Vulnerability of DPEphos to C−O Activation in the Presence of Nucleophilic Metal Hydrides

Mateusz K. Cybulski,[a] Nicholas A. Beattie,[b] Stuart A. Macgregor,*[b] Mary F. Mahon,[a] and Michael K. Whittlesey*[a]

General Comments. All manipulations were carried out using standard Schlenk, high vacuum and glovebox techniques using dry and degassed solvents. [Ru(IMe$_4$)$_2$(PPh$_3$)$_2$H$_2$] (**1**), [Ru(IEt$_2$Me$_2$)$_2$(PPh$_3$)$_2$H$_2$] (**3**) and [Ru(PPh$_3$)$_4$H$_2$] were prepared according to the literature.[1,2] DPEphos (Strem) was used as received. NMR spectra were recorded in [D$_6$]benzene (vacuum transferred from potassium) on Bruker Avance 500 and 400 MHz NMR spectrometers and referenced to δ = 7.15 ppm (^{1}H) and δ = 128.0 ppm (^{13}C). ^{31}P spectra were referenced to H_3PO_4 at δ = 0.0 ppm; spectra were also recorded in [D$_8$]toluene (vacuum transferred from potassium) for VT NMR studies of **6** or recorded unlocked in CH_2Cl_2 in the case of **7**. Elemental analyses were performed by Elemental Microanalysis Ltd, Okehampton, Devon, UK.

[Ru(IMe$_4$)$_2$(PPh$_3$)(Ph$_2$PC$_6$H$_4$O)H] (2). A 0.4 mL C_6H_6 solution of **1** (29.5 mg, 0.034 mmol) and DPEphos (22 mg, 0.040 mmol) was heated at 90 °C overnight in a J. Young's resealable NMR tube.‡ Subsequent addition of pentane resulted in precipitation of a pale yellow solid, which was further washed with pentane (3 x 0.5 mL) and dried in vacuo. A concentrated (0.2 mL) toluene solution of the solid was layered with pentane to afford **2** as yellow crystals. Yield: 13 mg (43%). ^{1}H NMR ([D$_6$]benzene, 500 MHz, 298 K): δ = 7.77 (s, 6H, s, ArH), 7.52 (m, 1H, ArH), 7.39 (m, 1H, ArH), 7.32 (s, 4H, ArH), 6.96 (s, 10H, ArH), 6.86 (m, 2H, ArH), 6.80 (m, 4H, ArH), 6.50 (m, 1H, ArH), 3.79 (s, 6H, NC*H*$_3$), 3.03 (s, 6H, NC*H*$_3$), 1.34 (s, 6H, C*H*$_3$), 1.23 (s, 3H, C*H*$_3$), -18.40 (t, ^2J(H,P) = 22.0 Hz, 1H, Ru*H*) ppm; ^{31}P{^{1}H} NMR ([D$_6$]benzene, 202 MHz, 298 K): δ = 51.3 (app s, presumably overlapping signals for P$_1$ and P$_2$) ppm; ^{13}C{^{1}H} NMR ([D$_6$]benzene, 126 MHz, 298 K): δ = 192.1 (t, ^2J(C,P) = 15 Hz, N*C*N), 178.4 (dd, ^2J(C,P) = 14 Hz, ^3J(C,P) = 12 Hz, OAr), 143.1 (dd, J(C,P) = 17 Hz, J(C,P) = 14 Hz, Ar), 141.5 (dd, J(C,P) = 18 Hz, J(C,P) = 15 Hz, Ar), 135.3 (s, Ar), 134.5 (t, J(C,P) = 6 Hz, Ar), 132.6 (t, J(C,P) = 6 Hz, Ar), 131.1 (s, Ar), 127.4 (s, Ar), 126.7 (m, Ar), 123.3 (s, N*C*CH$_3$), 123.2 (s, N*C*CH$_3$), 121.4 (t, J(C,P) = 3 Hz, Ar), 111.5 (t, J(C,P) =

2 Hz, Ar), 35.1 (s, N*C*H_3), 22.0 (s, N*C*H_3), 10.1 (s, *C*H_3), 8.8 (s, *C*H_3) ppm; elemental analysis (%) calcd for $C_{50}H_{54}N_4OP_2Ru$: C 67.48, H 6.11, N 6.29; found: C 68.02, H 6.08, N 6.30. ‡The formation of **2** could also be achieved (in ca. 5% yield) at room temperature, as shown by spectroscopic monitoring of a [D6]benzene solution of **1** (26 mg, 0.030 mmol) and DPEphos (19 mg, 0.035 mmol) over ca. 3 weeks at room temperature (Figure S4).

[Ru(IEt2Me2)(IEtMe2(C6H4)PPh2)(Ph2PC6H4O)H] (4). An Et_2O (0.3 mL) solution of **3** (31 mg, 0.033 mmol) and DPEphos (21.5 mg, 0.04 mmol) was refluxed overnight in a J. Young's resealable NMR tube to afford pale orange crystals, which were isolated by cannula filtration, washed with Et_2O (3 x 0.5 mL) and dried in vacuo. Yield: 12 mg (39%).^{1}H NMR ([D6]benzene, 500 MHz, 298 K): δ = 8.97 (br s, 1H, Ar*H*), 8.43 (br s, 2H, Ar*H*), 7.63 (dt, *J*(H,H) = 7.7 Hz, *J*(H,H) = 1.6 Hz, 1H, ArH), 7.35-7.20 (m, 4H, ArH), 7.08-6.95 (m, 4H, ArH), 6.91 (br s, 3H, ArH), 6.88-6.74 (m, 5H, ArH), 6.70-6.48 (m, 7H, ArH), 5.72 (m, 1H, NCH_2CH$_3$), 5.11 (m, 2H, NCH_2CH$_3$), 3.82 (m, 1H, NCH_2CH$_3$), 3.38 (m, 1H, NCH_2CH$_3$), 2.18 (m, 1H, NCH_2CH$_3$), 1.59 (s, 3H, NCCH_3), 1.53 (s, 3H, NCCH_3), 1.46 (s, 3H, NCCH_3), 1.42 (s, 3H, NCCH_3), 1.12 (t, ^3J(H,H) = 7.1 Hz, 3H, NCH$_2$CH_3), 0.85 (t, ^3J(H,H) = 7.0 Hz, 3H, NCH$_2$CH_3), 0.48 (t, ^3J(H,H) = 7.0 Hz, 3H, NCH$_2$CH_3), -17.71 (dd, ^2J(H,P) = 20.0 Hz, ^2J(H,P) = 15 Hz, 1H, Ru*H*) ppm; ^{31}P{^{1}H} NMR ([D6]benzene, 202 MHz, 298 K): δ = 59.3 (d, ^2J(P,P) = 29 Hz), 54.6 (d, ^2J(P,P) = 29 Hz) ppm; ^{13}C{^{1}H} NMR ([D6]benzene, 100 MHz, 298 K): δ = 194.5 (dd, ^2J(C,P) = 80 Hz, ^2J(C,P) = 21 Hz, N*C*N), 191.5 (dd, ^2J(C,P) = 88 Hz, ^2J(C,P) = 15 Hz, N*C*N), 179.2 (d, ^2J(C,P) = 23 Hz, OAr), 149.0 (dd, *J*(C,P) = 29 Hz, *J*(C,P) = 4 Hz, Ar), 143.4 (d, *J*(C,P) = 9 Hz, Ar), 139.8 (d, *J*(C,P) = 41 Hz, Ar), 135.3 (s, Ar), 134.3 (t, *J*(C,P) = 11 Hz, Ar), 133.8 (d, *J*(C,P) = 31 Hz, Ar), 132.3 (s, Ar), 130.8 (s, Ar), 130.2 (s, Ar), 124.7 (d, *J*(C,P) = 3 Hz, N*C*CH$_3$), 124.0 (t, *J*(C,P) = 2 Hz, N*C*CH$_3$), 123.8 (d, *J*(C,P) = 3 Hz, N*C*CH$_3$), 123.7 (d, *J*(C,P) = 2 Hz, N*C*CH$_3$), 43.8 (s, N*C*H$_2$CH$_3$), 43.5 (s, N*C*H$_2$CH$_3$), 41.4 (s, N*C*H$_2$CH$_3$), 16.1 (s, NCH$_2$*C*H$_3$), 14.4 (s, NCH$_2$*C*H$_3$), 14.0 (s, NCH$_2$*C*H$_3$), 11.1 (s, NC*C*H$_3$),

10.0 (s, CH_3), 9.3 (s, $NCCH_3$), 8.9 (s, $NCCH_3$) ppm; elemental analysis (%) calcd for $C_{52}H_{55}N_4OP_2Ru$: C 68.18, H 6.16, N 6.12; found: C 68.57, H 6.16, N 6.02.

[Ru(DPEphos)$_2$H$_2$] (5). [Ru(PPh$_3$)$_4$H$_2$] (300 mg, 0.26 mmol) and DPEphos (336 mg, 0.62 mmol) were dissolved in C_6H_6 (2 mL) in a J. Youngs resealable ampoule and stirred at room temperature for 8 h. The solution was filtered by cannula and layered with pentane to afford **5** as yellow crystals. Yield: 270 mg (88%). ^{1}H NMR ([D$_6$]benzene, 500 MHz, 298 K): δ = 7.78-7.31 (m, 12 H, ArH), 7.02-6.19 (m, 44 H, ArH), -9.80 (m, 2 H, Ru*H*) ppm; ^{31}P{^{1}H} NMR ([D$_6$]benzene, 202 MHz, 298 K): δ = 41.4 (t, ^2J(P,P) = 18 Hz), 35.3 (t, ^2J(P,P) = 18 Hz) ppm; elemental analysis (%) calcd for $C_{69}H_{55}O_2P_4Ru$: C 73.26, H 4.86; found: C 73.14, H 5.12.

[Ru(DPEphos)(Ph$_2$PC$_6$H$_4$O)H] (6) and [Ru(DPEphos)(Ph$_2$PC$_6$H$_4$O)Cl] (7). [Ru(PPh$_3$)$_4$H$_2$] (300 mg, 0.26 mmol) and DPEphos (310 mg, 0.57 mmol) were dissolved in THF (3 mL) in an ampoule fitted with a J. Young's resealable tap and heated at 80 °C overnight. The solution was filtered by cannula and the filtrate reduced to dryness to give **6** as a yellow solid. This was washed with pentane (3 x 10 mL), dried in vacuo and then recrystallised from toluene/pentane. Yield: 125 mg (51 %). ^{1}H NMR ([D$_6$]benzene , 500 MHz, 298 K): δ = 8.33-7.20 (m, 18H, Ar*H*), 7.09-6.33 (m, 24H, Ar*H*), -13.95 (q, ^2J(H,P) = 21.7 Hz, Ru*H*) ppm; ^{31}P{^{1}H} NMR ([D$_6$]benzene, 202 MHz, 298 K): δ = 76.8 (t, ^2J(P,P) = 30.1 Hz, Ph$_2$*P*C$_6$H$_4$O), 49.9 (br s, DPEphos) ppm; elemental analysis (%) calcd for $C_{54}H_{43}O_2P_3Ru \cdot C_7H_8$: C 72.53, H 5.09; found: C 72.49, H 5.13. Attempts to grow crystals of **6** proved unsuccessful, but upon layering a saturated CD_2Cl_2 solution of the complex with pentane, a small amount of X-ray quality crystalline material was obtained which comprised of a mixture of **6** and [Ru(DPEphos)(Ph$_2$PC$_6$H$_4$O)Cl] (**7**) (see crystallography section for details).

[Ru(DPEphos)($Ph_2PC_6H_4O$)Cl] (7). [Ru(DPEphos)($Ph_2PC_6H_4O$)H] (20 mg, 0.022 mmol) was refluxed for 10 h in CD_2Cl_2 (0.5 mL) or in [D$_6$]benzene containing 5 equiv benzyl chloride (14 mg, 0.11 mmol) in J. Young's resealable NMR tubes. In both cases, spectroscopic monitoring showed formation of **7**. The compound was accessible on a preparative scale by overnight reflux of [Ru(DPEphos)($Ph_2PC_6H_4O$)H] (60 mg, 0.06 mmol) in CH_2Cl_2 in a J. Young's resealable NMR tube. An orange microcrystalline solid was obtained upon layering the solution with Et_2O. The solid was washed with Et_2O (3 x 0.5 mL) and dried in vacuo. Yield: 26 mg (42%). $^{31}P\{^1H\}$ NMR (CH_2Cl_2, 202 MHz, 298 K): δ = 64.5 (t, ^2J(P,P) =30 Hz, $Ph_2P(C_6H_4)O$), 35.0 (br, DPEphos), 30.7 (br, DPEphos) ppm; elemental analysis (%) calcd for $C_{54}H_{42}O_2P_3ClRu{\cdot}0.3CH_2Cl_2$: C 66.69, H 4.39; found: C 66.74, H 4.24.

X-ray crystallography. Data for **2**, **5** and **7** were collected on an Agilent SuperNova diffractometer, using a Cu-Kα source, while those for **4** were obtained using an Agilent Xcalibur instrument equipped with Mo-Kα radiation. All experiments were conducted at 150 K. Convergences were achieved using SHELX[3] via Olex2[4] and were relatively straightforward. Only points of note are mentioned hereafter. The hydrogen atoms in **2** were included at calculated positions with the exception of the hydride ligand. This was located and refined subject to being a distance of 1.6 Å from Ru1. The U_{iso} values for the each of the hydrogens attached to C4 and C14 were refined freely, as two of these fall within the range of being involved in typical C-H···O interactions. The residual electron density in this structure was explored at length. It is indicative of disorder in the Ph-O-Ph moiety, after cleavage of the C-O bond during the reaction. The electron density maximum is at a position that suggests disorder with O1. A model was constructed for this disorder but, as it is in the region of 10%, it was abandoned in the interests of negating the mandatory inclusion of multiple restraints for the minor component in order to secure a chemically meaningful convergence.

Treatment of the hydride ligands in **4** and **5** (Figure S14) was similar to that in **2**. The asymmetric unit in **5** was also host to half of a guest molecule of benzene. In addition to one ruthenium complex molecule, the asymmetric unit in **7** was seen to contain one full molecule of dichloromethane and a separate solvent region that equated to 0.7 molecules of CH_2Cl_2 disordered over two sites in a 40:30 ratio. C-Cl distances were restrained to being similar in both of the latter disordered components. The main feature in **7** was also a disorder composite, such that the motif represents 75% of a chloride complex overlaid with 25% of the analogous hydride complex. The included 25% hydride ligand (H1) in the minor component is tenuous in terms of refining it from the electron density and it is really included for completeness. The Ru1-H1 distance was restrained to a value of 1.6 Å (as per hydride treatment in the other structures presented here) and the U_{iso} for the hydride was allowed to ride on that for the metal center. Interestingly, however, the phenyl ring based on C25 refined to a site-occupancy of 75% (quite separately to the refinement of the 75% occupancy for Cl1). The corresponding disordered minor component (25% site occupancy) of this ring (based on C25A) is more proximate to the Ru1-H1/Cl1 vectors, which corroborates the presence of a less sterically demanding ligand (i.e. H1) within the crystal in one quarter of instances. The ring based on C25A was treated as a rigid hexagon. ADP restraints were also incorporated into the model for the atoms therein and, finally, the P2-C25 and P2-C25A distances were refined subject to being similar.

CCDC 1532000-1532003 contain the supplementary crystallographic data for compounds **2**, **4**, **5** and **7** respectively. These data can be obtained free of charge at http://www.ccdc.cam.ac.uk/conts/retrieving.html, or from the Cambridge Crystallographic Data Centre, CCDC, 12 Union Road, Cambridge CB2 1EZ, UK (Fax: 44-1223-336-033; or E-Mail: deposit@ccdc.cam.ac.uk).

Table S1. Crystallographic details for **2**, **4** and **7**.

Identification code	**2**	**4**	**7**
Empirical formula	$C_{50}H_{54}N_4OP_2Ru$	$C_{52}H_{56}N_4OP_2Ru$	$C_{55.7}H_{44.9}Cl_{4.15}O_2P_3Ru$
Formula weight	889.98	916.01	1087.31
Temperature/K	150.00(10)	149.99(10)	150.01(10)
Crystal system	monoclinic	triclinic	monoclinic
Space group	$P2_1/n$	P–1	$P2_1/c$
a/Å	19.76710(12)	11.0706(6)	12.1394(2)
b/Å	10.96787(7)	12.4859(6)	36.4738(6)
c/Å	20.23805(12)	17.8815(9)	12.1334(2)
α/°	90	73.508(4)	90
β/°	95.4189(6)	85.536(4)	111.404(2)
γ/°	90	68.278(5)	90
U/Å^3	4368.06(5)	2200.7(2)	5001.77(17)
Z	4	2	4
ρ_{calc} /g cm^{-3}	1.353	1.382	1.444
μ/mm^{-1}	3.918	0.473	5.812
F(000)	1856.0	956.0	2219.0
Crystal size/mm^3	0.217 × 0.148 × 0.107	0.18 × 0.107 × 0.063	0.24 × 0.084 × 0.031
Radiation	CuKα (λ = 1.54184)	MoKα (λ = 0.71073)	CuKα (λ = 1.54184)
2θ range for data collection/°	8.988 to 143.898	6.684 to 54.966	7.822 to 146.782
Index ranges	–24 ≤ h ≤ 24, –13 ≤ k ≤ 11, –24 ≤ l ≤ 24	–14 ≤ h ≤ 13, –16 ≤ k ≤ 15, –23 ≤ l ≤ 23	–15 ≤ h ≤ 13, –44 ≤ k ≤ 44, –14 ≤ l ≤ 13
Reflections collected	61367	20381	59637
Independent reflections	8588, 0.0362	10088 0.0550	9999, 0.0499
Data/restraints/parameters	8588/1/541	10088/1/552	9999/44/676
Goodness–of–fit on F^2	1.029	1.001	1.152
Final $R1$,$wR2$ [I>=2σ (I)]	0.0296, 0.0725	0.0518, 0.0852	0.0421, 0.0995
Final $R1$,$wR2$ [all data]	0.0313, 0.0738	0.0894, 0.0974	0.0447, 0.1009
Largest diff. peak/hole / e Å^{-3}	1.41/–0.51	0.51/–0.59	0.78/–0.64

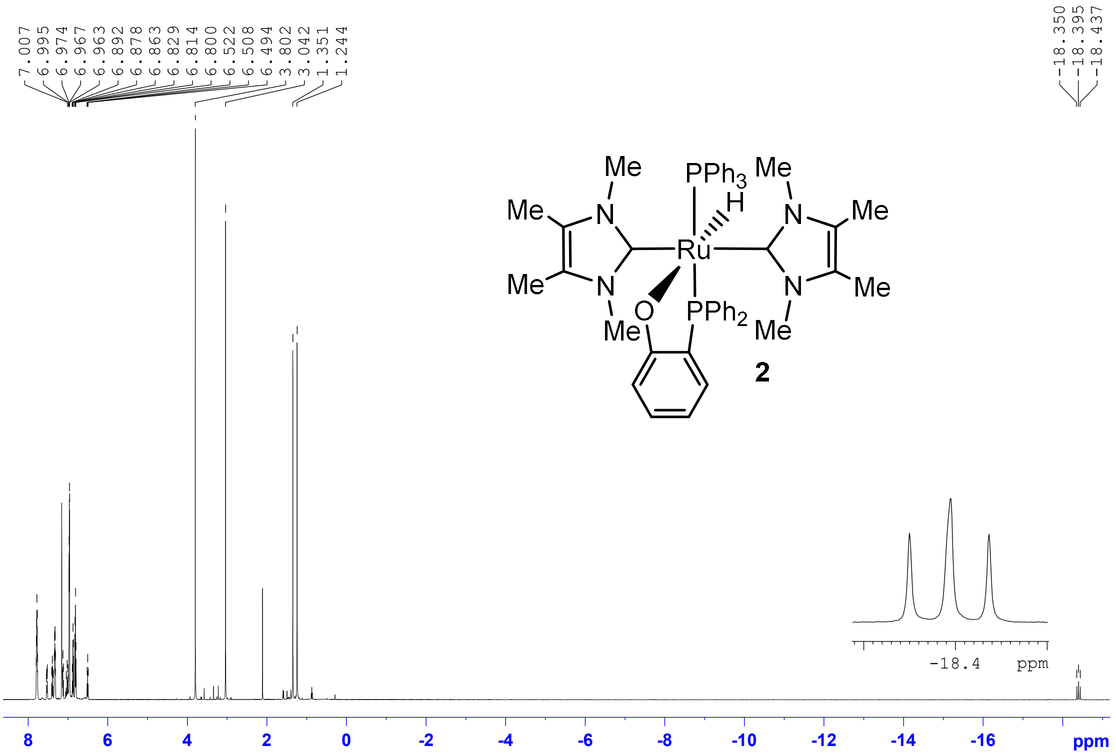

Figure S1. ^{1}H NMR spectrum ([D6]benzene, 500 MHz, 298 K) of **2**.

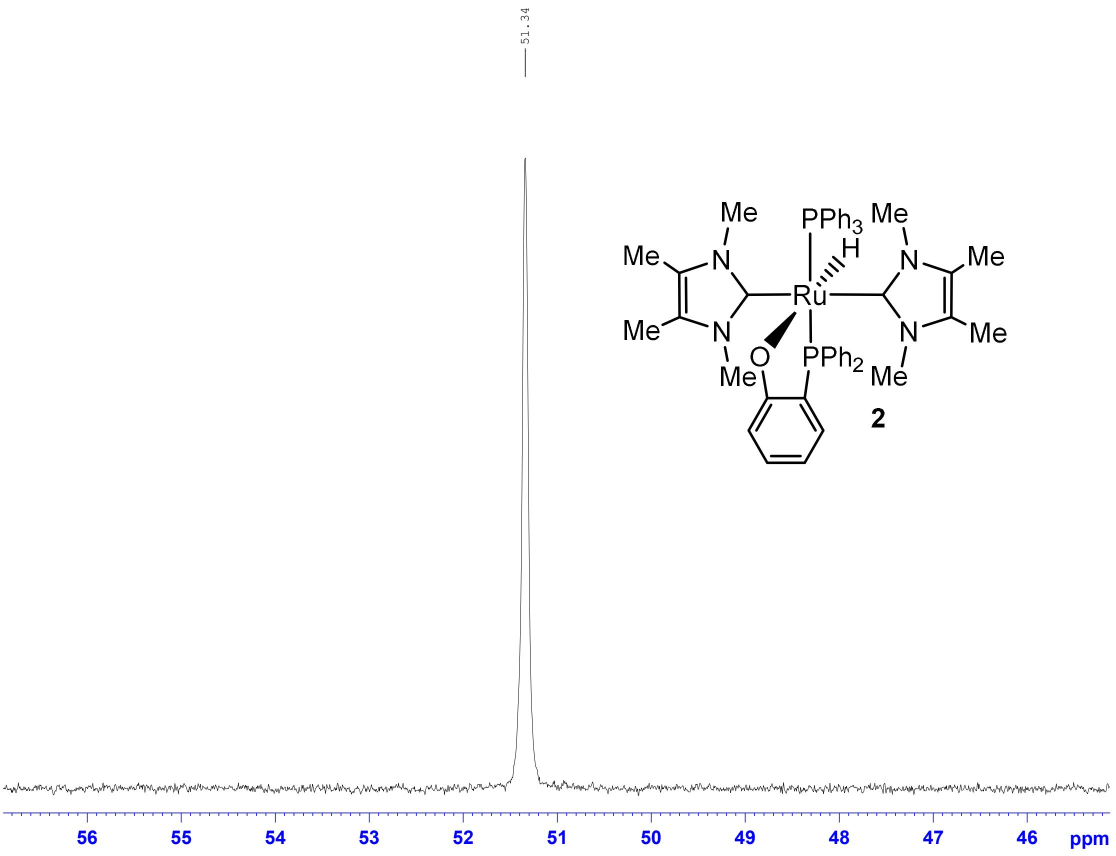

Figure S2. $^{31}P\{^{1}H\}$ NMR spectrum ([D6]benzene, 202 MHz, 298 K) of **2**.

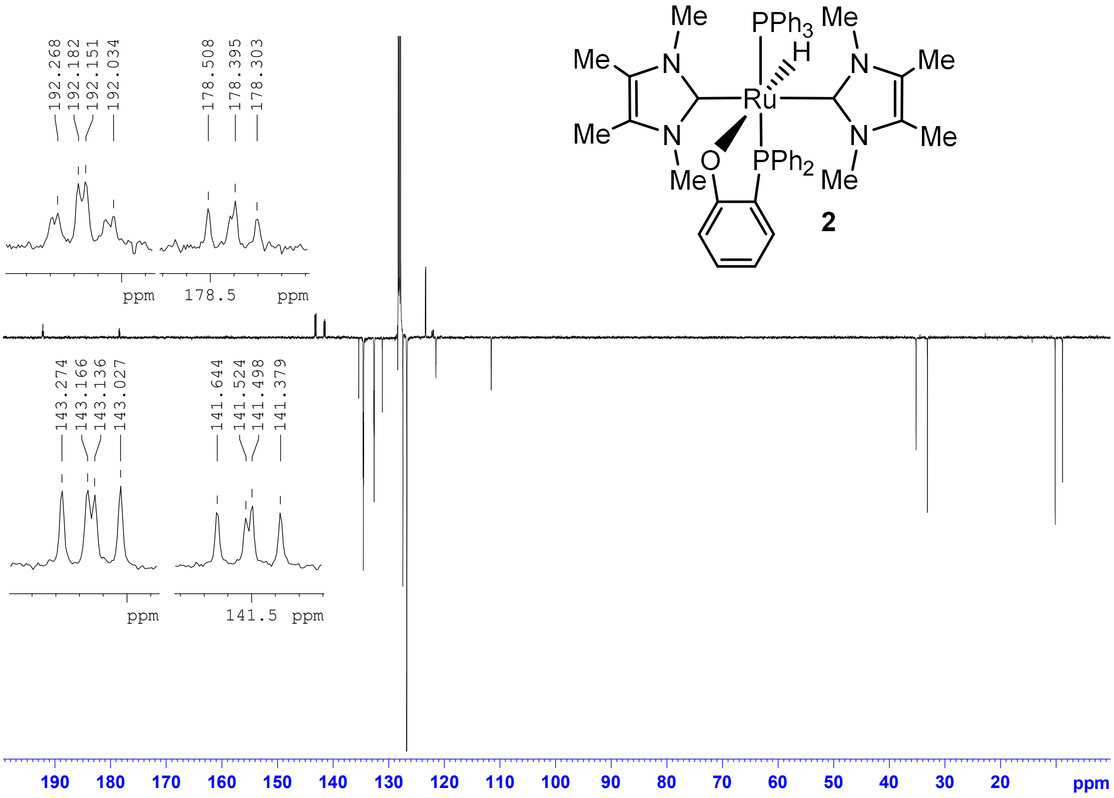

Figure S3. $^{13}C\{^{1}H\}$ PENDANT NMR spectrum ([D$_6$]benzene, 126 MHz, 298 K) of **2**.

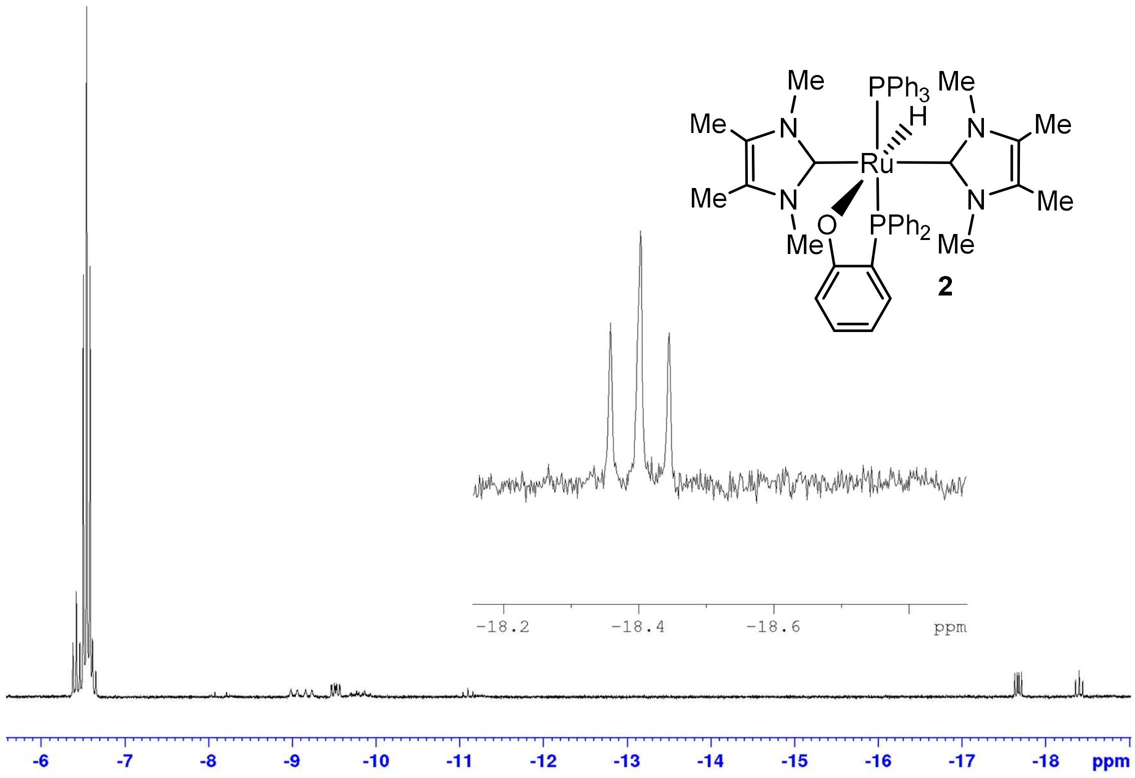

Figure S4. Low frequency region of a ^{1}H NMR spectrum ([D$_6$]benzene, 500 MHz, 298 K) of the reaction of **1** with DPEphos recorded after 20 days at room temperature, illustrating the formation of **2** (Ru-H signal of **2** expanded in the inset). Note that the signal for **1** at ca. δ = -6.5 ppm is made complex by H/D exchange with solvent.

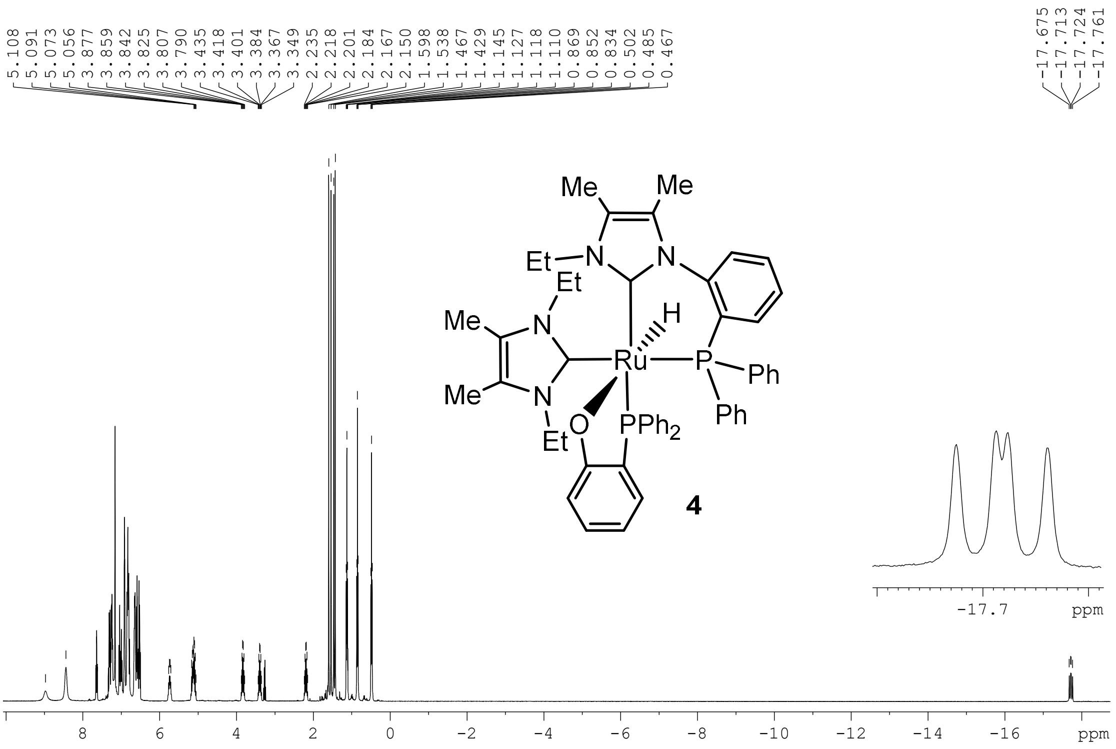

Figure S5. ^{1}H NMR spectrum ([D$_6$]benzene, 500 MHz, 298 K) of **4**.

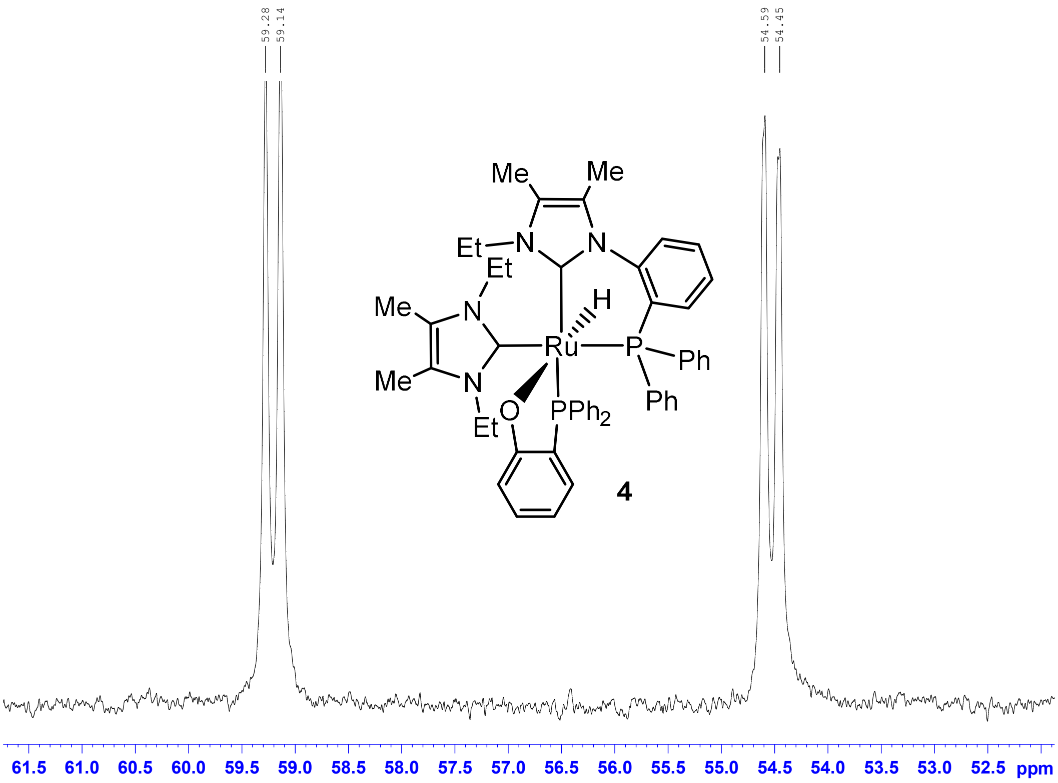

Figure S6. ^{31}P{^{1}H} NMR spectrum ([D$_6$]benzene, 202 MHz, 298 K) of **4**.

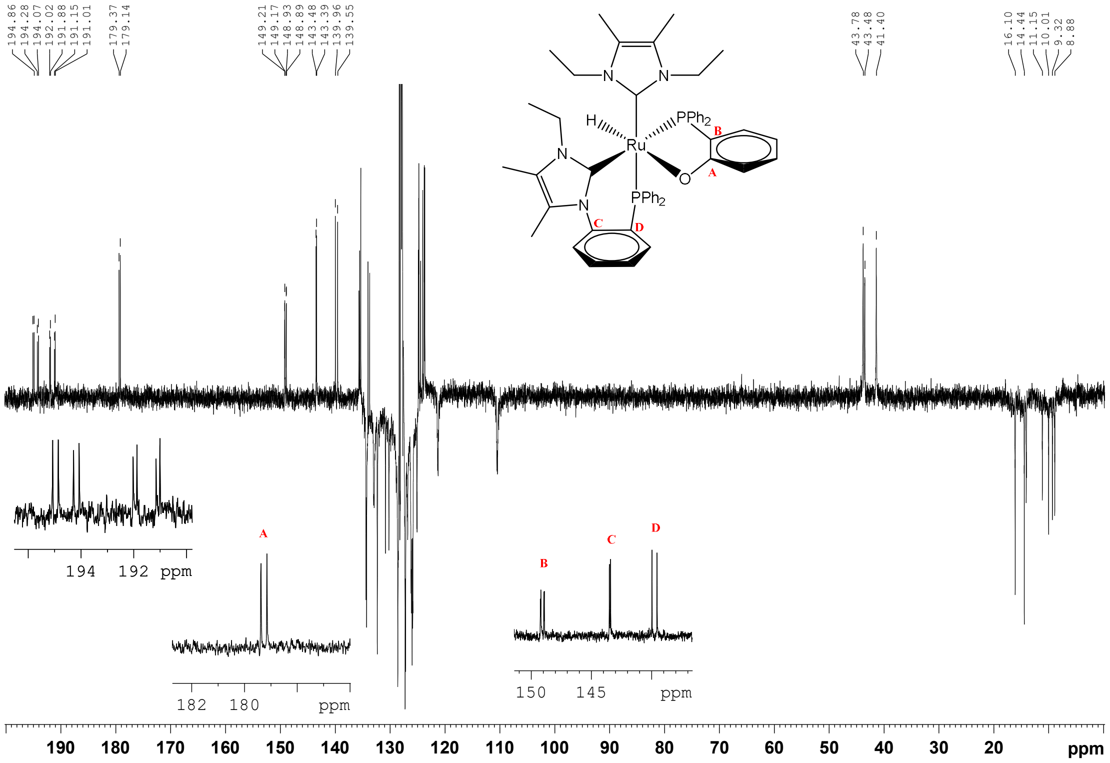

Figure S7. $^{13}C\{^{1}H\}$ PENDANT NMR spectrum ([D$_6$]benzene, 126 MHz, 298 K) of **4**.

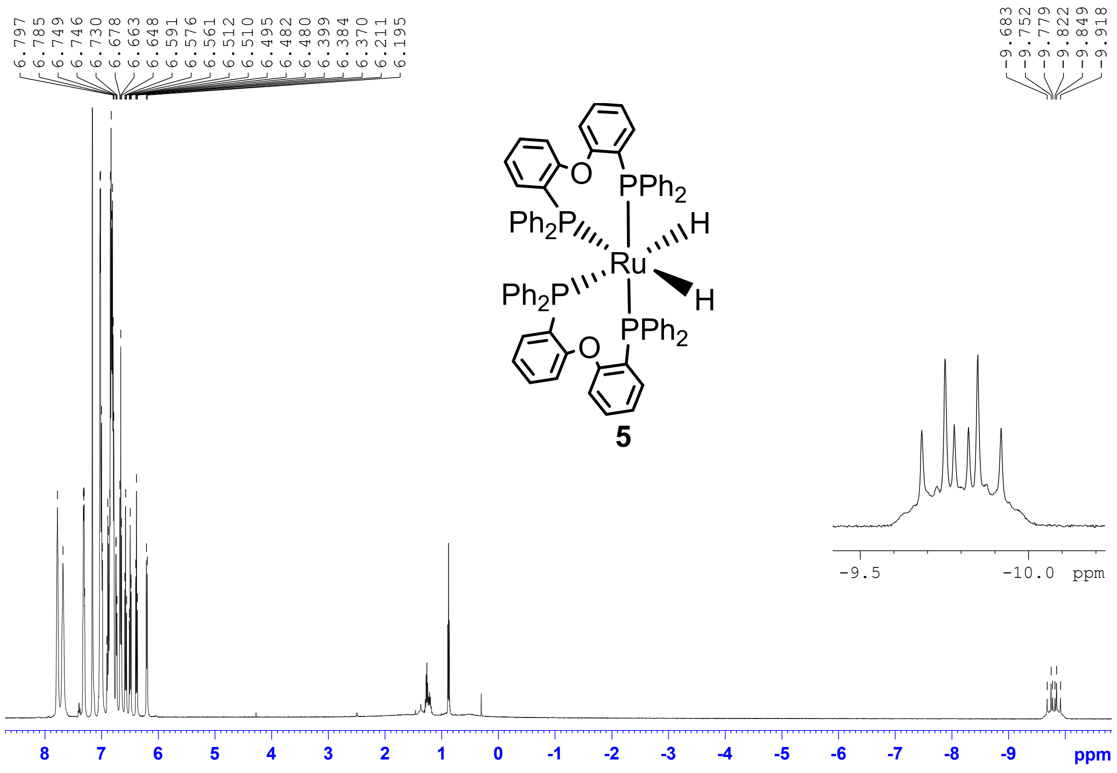

Figure S8. ^{1}H NMR spectrum ([D$_6$]benzene, 500 MHz, 298 K) of **5**.

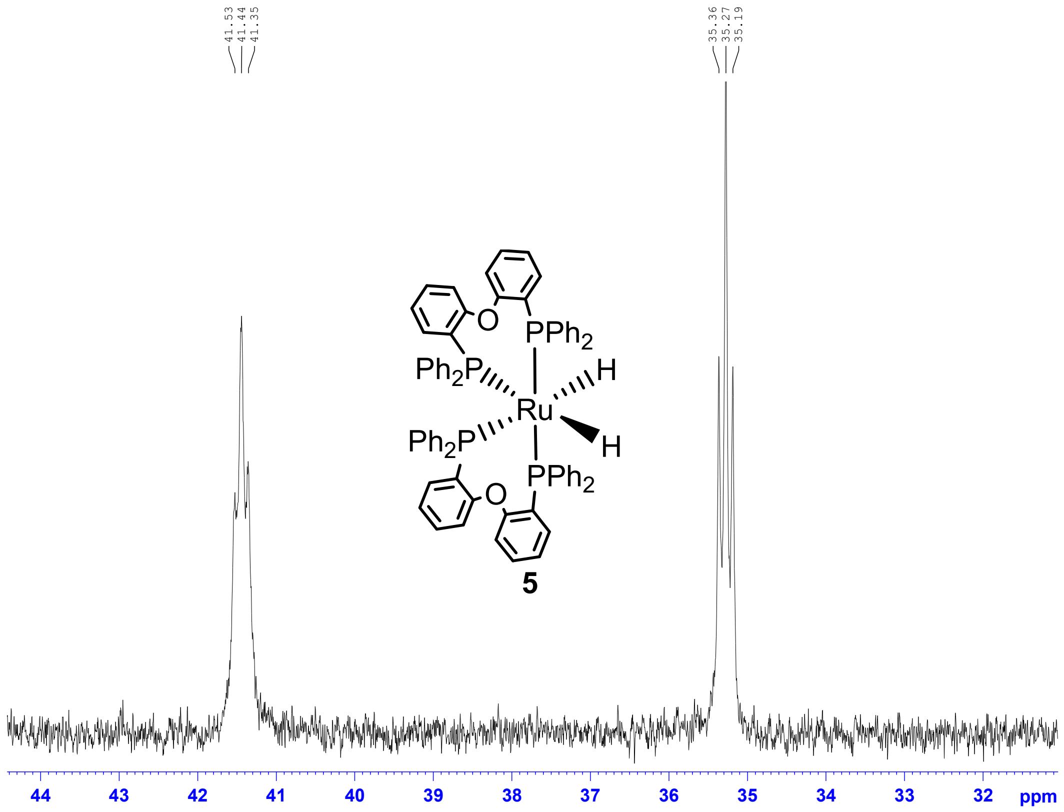

Figure S9. $^{31}P\{^{1}H\}$ NMR spectrum ([D$_6$]benzene, 202 MHz, 298 K) of **5**.

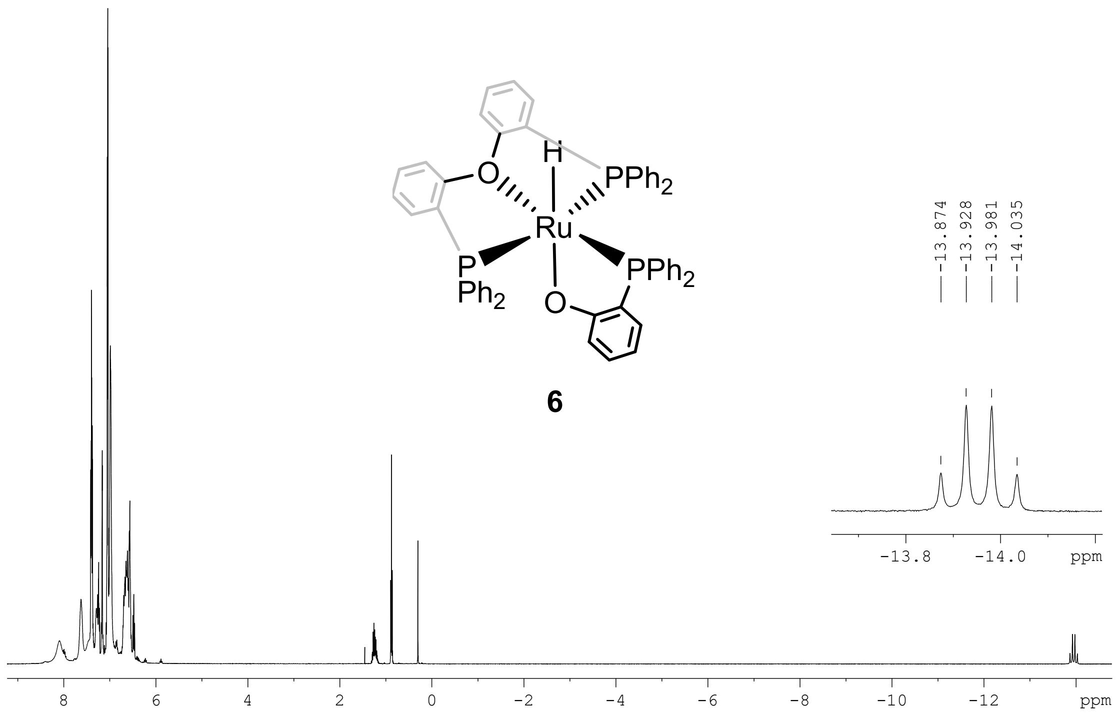

Figure S10. ^{1}H NMR spectrum ([[D$_6$]benzene, 500 MHz, 298 K) of **6**.

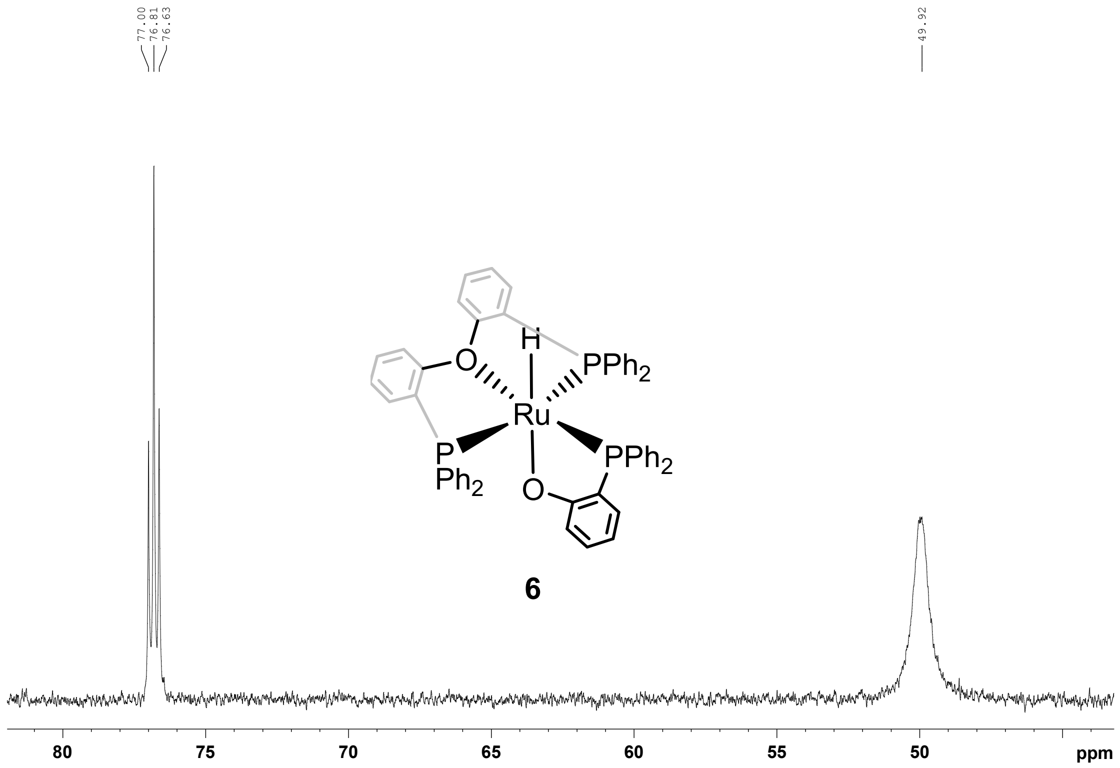

Figure S11. $^{31}P\{^{1}H\}$ NMR spectrum ([D$_6$]benzene, 202 MHz, 298 K) of **6**.

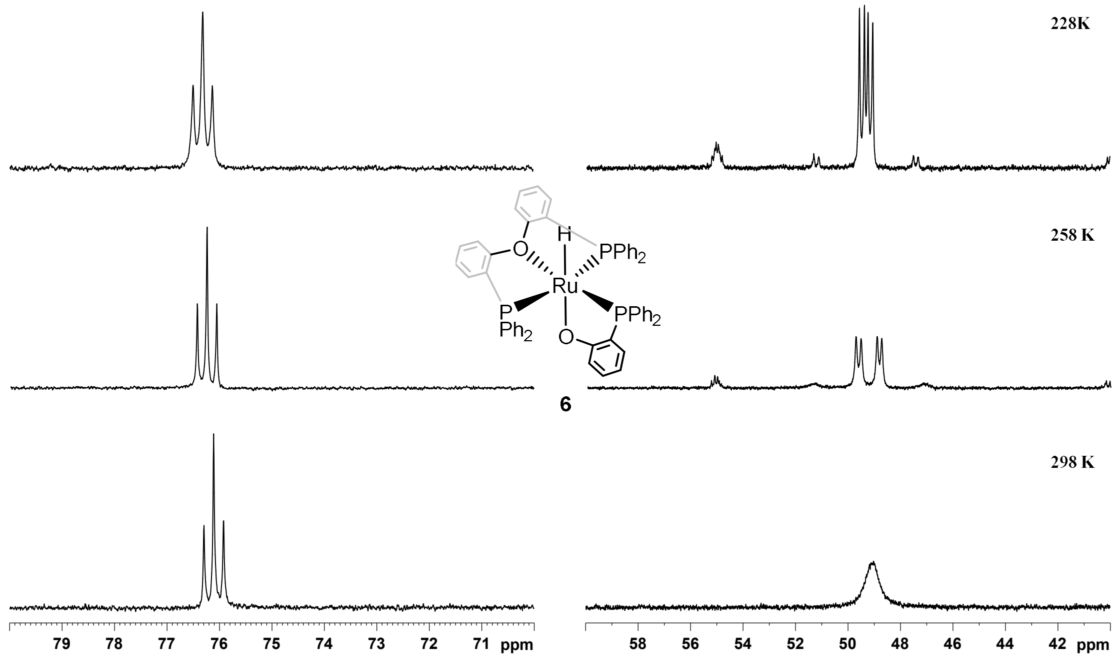

Figure S12. Variable temperature $^{31}P\{^{1}H\}$ NMR spectra ([D$_8$]toluene, 400 MHz) of **6**.

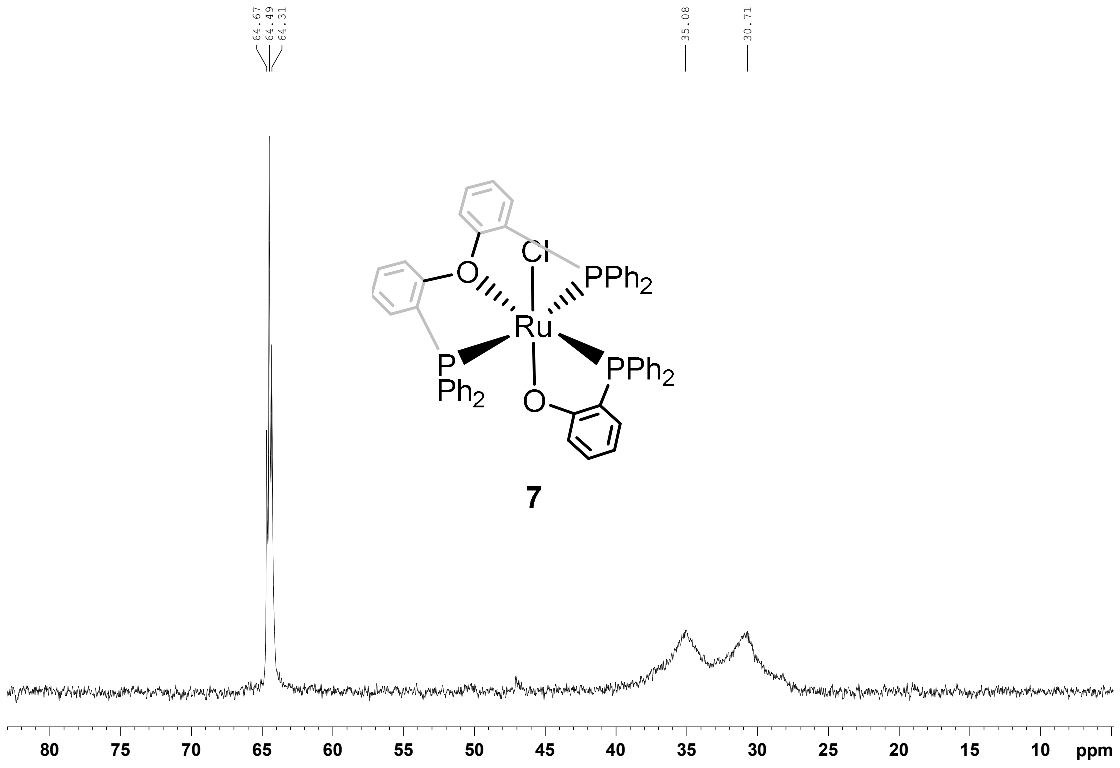

Figure S13. $^{31}P\{^{1}H\}$ NMR spectrum (CH_2Cl_2, 202 MHz, 298 K) of **7**.

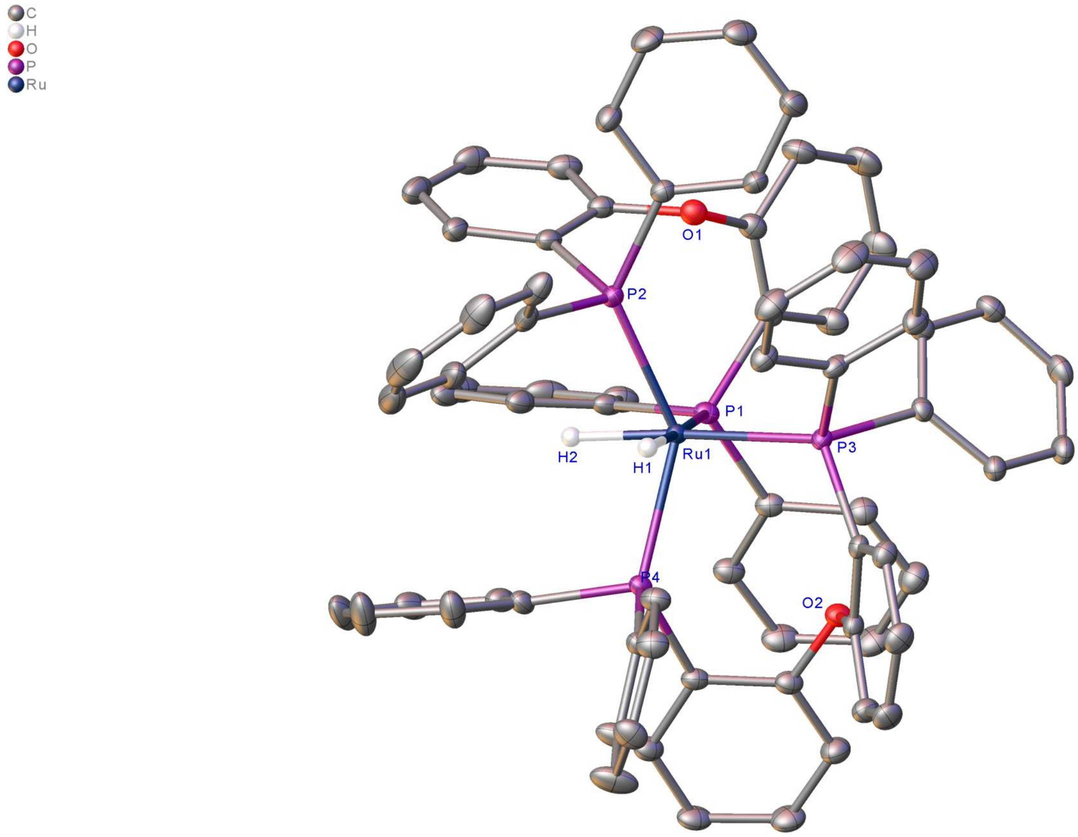

Figure S14. Molecular structure of cis-Ru(DPEphos)$_2$H$_2$ (**5**). Ellipsoids are shown at the 30% level with all hydrogen atoms (except Ru-H) removed for clarity. Selected bond lengths (Å) and angles (°): Ru(1)-P(1) 2.4108(5), Ru(1)-P(2) 2.3179(5), Ru(1)-P(3) 2.3827(5), Ru(1)-P(4) 2.3156(5), P(1)-Ru(1)-P(3) 104.96(17), P(2)-Ru(1)-P(4) 138.806(18).

Computational Details. DFT calculations were run with Gaussian 09 (Revision D.01).[5] Ru and P centres were described with the Stuttgart RECPs and associated basis sets[6] and 6-31G** basis sets were used for all other atoms.[7] A set of d-orbital polarisation functions was also added to P (ζ^d =0.387).[8] Optimizations employed the BP86 functional[9] and all stationary points were fully characterised via analytical frequency calculations as either minima (all positive eigenvalues) or transition states (one negative eigenvalue). Transition states were also characterised via IRC calculations and subsequent geometry optimizations confirmed they linked to the minima as reported in the text or in below in the Supporting Information. All stationary points were subjected to rigorous conformational searching (details below) and the lowest energy conformer is reported in each case. SCF energies were corrected for the effects of benzene solvent via the polarizable continuum model (PCM[10]) and for dispersion using Grimme's D3 parameter set with Becke-Johnson damping.[11] Natural Bonding Orbital analyses employed the NBO 6.0 programs[12] and all geometries are supplied as a separate xyz file readable by Chemcraft[13] and Mercury.[14]

The following protocol was adopted to ensure that the lowest energy conformations were located for each stationary point.[15] For each initial structure (derived from experiment or derived from an initial optimisation or TS search) a molecular dynamics (MD) simulation was performed using the Tinker program[16] and the MM3 force field. These runs allowed movement of the substituents on the phosphine and IMe_4 ligands, with all other bonds being fixed. Additional force field parameters were added as required to describe the latter. MD simulations were run for 1 ns with a 1 fs time step in an NVT ensemble, with coordinates being collected every picosecond to generate 1000 structures. The trajectories were propagated using the modified Beeman integration algorithm,[17] and a Berendsen thermostat[18] was used to keep the temperature around 1000 K. This temperature was needed to span the conformational space in the most efficient way. The 1000 generated structures

were then optimised with the MM3 force field with the same geometry constraints as above. Energetically unique structures (typically between 5 and 100 conformations for each stationary point) were then selected for optimization at the DFT level using the approach described above. The lowest energy structure was then fully characterised with a frequency calculation. For transition states, key distances (Ru-H^1, H^1-C^1, C^1-O and O···Ru) were fixed for the MD and MM3 minimisations, as well as for the initial DFT optimisation. A full TS optimisation was then performed on the lowest energy structure. In some cases where >100 conformations were located (typically in the [Ru(DPEphos)$_2$H$_2$] systems) an initial optimization at the BP86 level was performed with the lanl2dz basis sets and ECPs on Ru and P[19] and 6-31g basis sets[7] on other atoms (while maintaining the ultrafine grid). The lowest energy structures were then optimised and characterised with the full basis sets above.

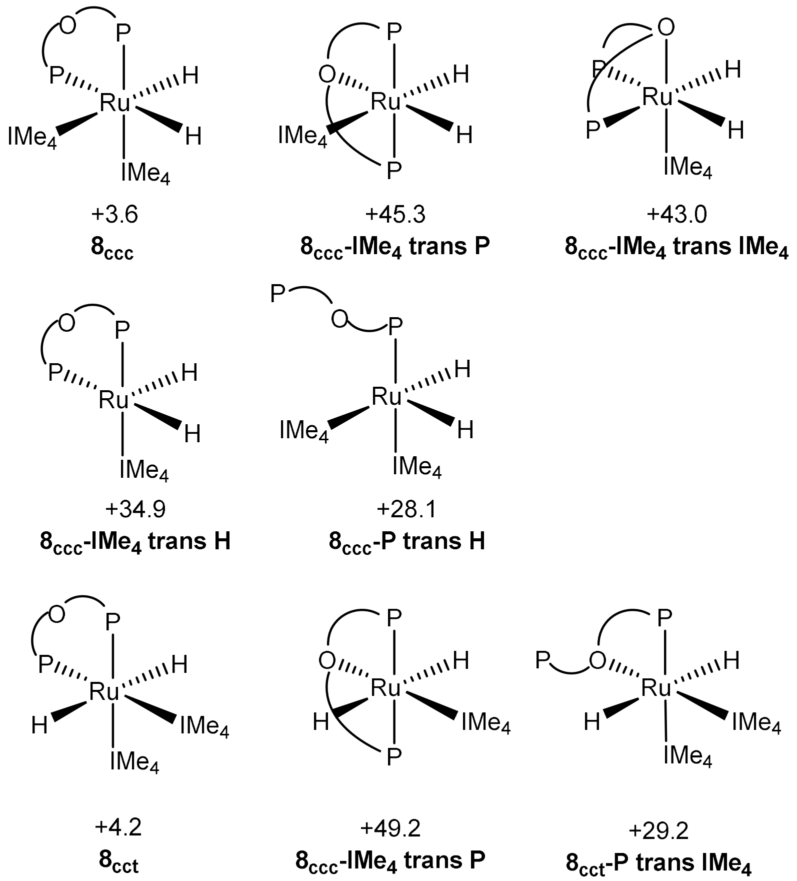

Figure S15. Computed ligand dissociation free energies (kcal/mol, BP86(benzene, D3BJ)) in [Ru(IMe$_4$)$_2$(DPEphos)H$_2$].

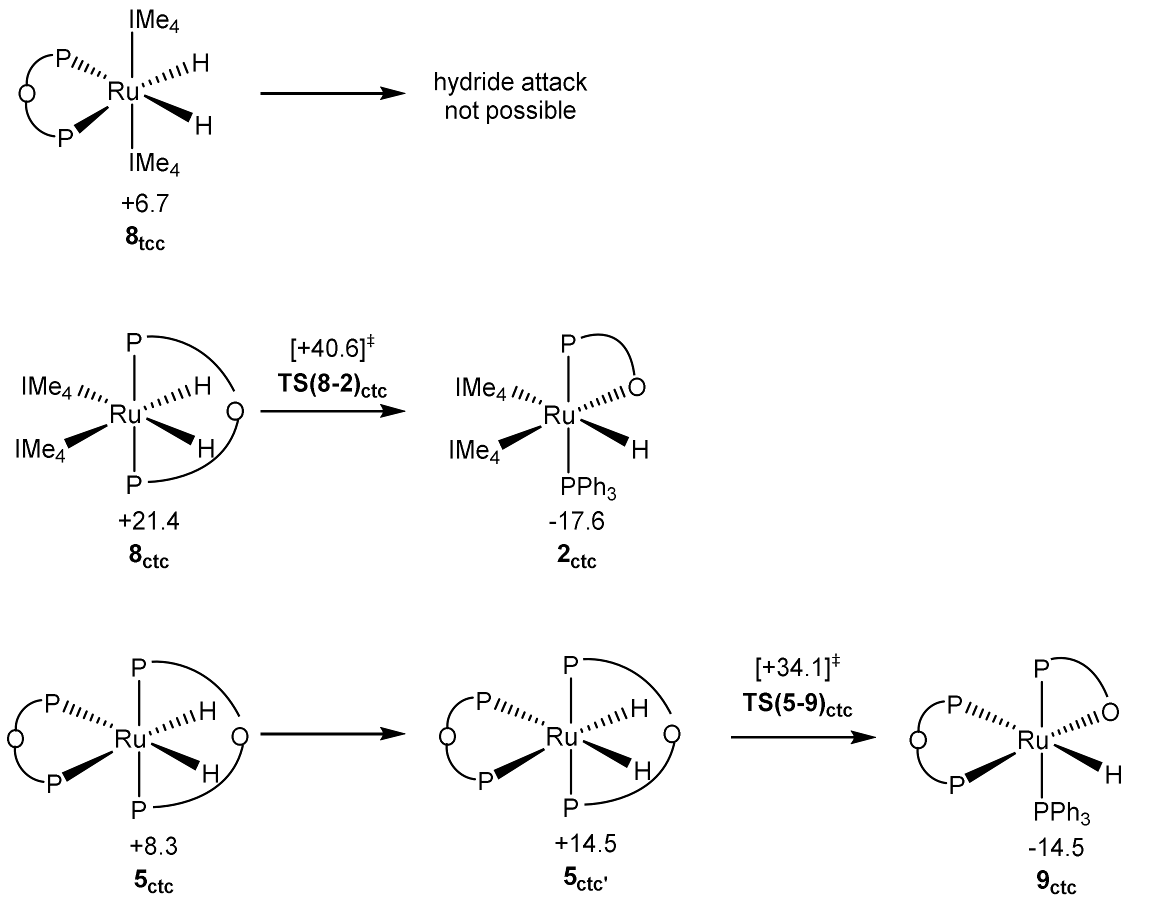

Figure S16. Free energy profiles (kcal/mol, BP86(benzene, D3BJ)) for C-O activation in alternative isomers of [Ru(IMe$_4$)$_2$(DPEphos)H$_2$] and [Ru(DPEphos)$_2$H$_2$].

1. Formation and reactivity of Ru((IMe4)2(DPEphos)H2

1

SCF = -2266.24091529
H(0 K)= -2265.332508
G(298 K)= -2265.427258
SCF(C6H6) = -2266.24612511
BP86(D3BJ) = -2266.59927766
Low Freq. = 16.1734cm-1, 17.8349cm-1

113
C -3.20793 -2.59132 0.74636
C -3.14690 -1.24562 1.17222
C -3.64805 -0.92557 2.45002
C -4.19017 -1.92071 3.28178
C -4.24255 -3.25503 2.84968
C -3.75063 -3.58580 1.57499
P -2.33270 0.02658 0.02876
C -3.33856 -0.39116 -1.52279
C -2.68747 -0.45573 -2.76769
C -3.39908 -0.77261 -3.93811
C -4.77660 -1.03301 -3.87565
C -5.43772 -0.98188 -2.63601
C -4.72414 -0.66858 -1.46858
Ru 0.00007 0.07742 -0.00011
C 0.00052 2.18084 -0.00024
N 0.46542 3.06212 0.97316
C 0.30135 4.40663 0.61620
C -0.29905 4.40665 -0.61723
N -0.46390 3.06215 -0.97386
C 1.07096 2.66260 2.23428
C -1.06994 2.66263 -2.23474
P 2.33281 0.02545 -0.02863
C 3.14643 -1.24734 -1.17183
C 3.64781 -0.92765 -2.44964
C 4.18937 -1.92315 -3.28132
C 4.24092 -3.25749 -2.84917
C 3.74876 -3.58790 -1.57448
C 3.20664 -2.59305 -0.74590
C 3.18240 1.60604 -0.61346
C 2.68327 2.20554 -1.79278
C 3.25292 3.38504 -2.29623
C 4.31495 4.00430 -1.61567
C 4.80133 3.43248 -0.43065
C 4.24373 2.23998 0.06442
C 3.33823 -0.39258 1.52311
C 2.68713 -0.45624 2.76805
C 3.39854 -0.77321 3.93857
C 4.77587 -1.03462 3.87617
C 5.43700 -0.98440 2.63651
C 4.72362 -0.67101 1.46898
C -0.00048 -2.03932 -0.00015
N -0.07424 -2.92168 1.07811
C -0.04913 -4.26556 0.68453
C 0.04645 -4.26559 -0.68485
N 0.07258 -2.92173 -1.07844
C -0.22936 -2.54617 2.47878
C 0.22826 -2.54635 -2.47908
C -3.18133 1.60766 0.61361
C -2.68181 2.20694 1.79287
C -3.25082 3.38674 2.29632
C -4.31260 4.00652 1.61583
C -4.79937 3.43490 0.43086
C -4.24239 2.24210 -0.06421
C -0.13487 -5.39803 1.65774
C 0.13114 -5.39812 -1.65808
C -0.73996 5.54329 -1.48326
C 0.74297 5.54321 1.48195
H -0.11824 0.11459 1.68554
H -0.73528 3.33377 -3.04276
H -2.17238 2.69160 -2.17630
H -0.74295 1.63063 -2.43084
H 0.73604 3.33377 3.04218
H 2.17343 2.69149 2.17626
H 0.74377 1.63065 2.43031
H -1.15111 -2.98934 2.89155
H -0.29847 -1.44704 2.50275
H 0.63988 -2.88524 3.06887
H -0.64100 -2.88498 -3.06938
H 1.14989 -2.99003 -2.89158
H 0.29796 -1.44726 -2.50309
H 5.62400 3.91025 0.11325
H 4.75545 4.92906 -2.00400
H 2.86047 3.82636 -3.21925
H 1.82924 1.73849 -2.29540
H -5.24666 -0.64799 -0.50628
H -6.51131 -1.19275 -2.57660
H -5.33352 -1.28248 -4.78552
H -2.87367 -0.82066 -4.89876
H -1.60645 -0.27149 -2.78896
H 4.64291 1.80463 0.98533
H 0.11854 0.11434 -1.68578
H 3.63089 0.10903 -2.79901
H 4.58023 -1.64869 -4.26750
H 4.66967 -4.03197 -3.49439
H 3.79289 -4.62314 -1.21797
H 2.83106 -2.86675 0.24538
H 1.60624 -0.27121 2.78932
H 2.87311 -0.82055 4.89924
H 5.33263 -1.28415 4.78612
H 6.51044 -1.19605 2.57714
H 5.24613 -0.65111 0.50666
H -1.82800 1.73945 2.29547
H -2.85808 3.82789 3.21930
H -4.75260 4.93150 2.00417
H -5.62183 3.91306 -0.11299
H -4.64186 1.80692 -0.98508
H -3.63047 0.11112 2.79935
H -4.58081 -1.64596 4.26797
H -4.67176 -4.02921 3.49496
H -3.79543 -4.62102 1.21852
H -2.83252 -2.86530 -0.24492
H 1.82521 5.49944 1.70193
H 0.54671 6.50222 0.97716
H 0.21134 5.56768 2.45153
H -0.54332 6.50229 -0.97859
H -0.20815 5.56736 -2.45275
H -1.82218 5.50002 -1.70343
H -1.08037 -5.37702 2.23026
H -0.08705 -6.36198 1.12685
H 0.69084 -5.38168 2.39263
H 0.08278 -6.36203 -1.12718
H -0.69476 -5.38120 -2.39275
H 1.07649 -5.37780 -2.23085

DPEphos

SCF = -1477.02441902
H(0 K)= -1476.509312
G(298 K)= -1476.583577
SCF(C6H6) = -1477.02930551
BP86(D3BJ) = -1477.20362745
Low Freq. = 7.4117cm-1, 9.2942cm-1

67
C -2.31994 -1.41136 0.56461
C -1.00083 -1.91237 0.66153
C -0.69216 -3.03023 1.45600
C -1.71508 -3.67509 2.16481
C -3.03461 -3.20054 2.08573
C -3.32688 -2.07820 1.29537
O 0.00006 -1.20335 0.00013
C 1.00093 -1.91243 -0.66124
C 0.69222 -3.03034 -1.45563
C 1.71511 -3.67526 -2.16443
C 3.03465 -3.20072 -2.08542
C 3.32696 -2.07833 -1.29515
C 2.32005 -1.41144 -0.56439
P 2.62796 0.02294 0.60355
C 4.50321 0.00532 0.67611
P -2.62779 0.02294 -0.60345
C -4.50303 0.00516 -0.67631

```
C  -2.32890  1.49433  0.51487
C   2.32877  1.49426 -0.51478
H   0.34274 -3.37960  1.51385
H  -1.47560 -4.54499  2.78528
H  -3.83566 -3.70102  2.63918
H  -4.35637 -1.71282  1.22932
H   4.35646 -1.71296 -1.22915
H   3.83568 -3.70124 -2.63887
H   1.47560 -4.54520 -2.78483
H  -0.34269 -3.37970 -1.51343
C   5.34861  0.71370 -0.20404
C   6.74360  0.66757 -0.04376
C   7.31282 -0.08916  0.99332
C   6.48064 -0.79652  1.87690
C   5.08681 -0.74131  1.72407
H   4.91143  1.30692 -1.01371
H   7.38735  1.22523 -0.73293
H   8.40052 -0.12211  1.11714
H   6.91670 -1.38268  2.69311
H   4.44002 -1.27819  2.42824
C   2.22068  2.75505  0.11218
C   2.01197  3.91588 -0.64735
C   1.88852  3.83033 -2.04414
C   1.97711  2.57939 -2.67546
C   2.19938  1.41777 -1.91711
H   2.29641  2.82507  1.20341
H   1.93538  4.88689 -0.14650
H   1.71741  4.73487 -2.63762
H   1.87517  2.50480 -3.76370
H   2.27113  0.44607 -2.41615
C  -2.22078  2.75510 -0.11214
C  -2.01228  3.91597  0.64737
C  -1.88908  3.83050  2.04419
C  -1.97768  2.57959  2.67555
C  -2.19974  1.41792  1.91722
H  -2.29633  2.82506 -1.20338
H  -1.93568  4.88697  0.14649
H  -1.71813  4.73508  2.63765
H  -1.87593  2.50506  3.76382
H  -2.27151  0.44623  2.41630
C  -5.34863  0.71349  0.20369
C  -6.74358  0.66724  0.04319
C  -7.31258 -0.08958 -0.99395
C  -6.48020 -0.79689 -1.87738
C  -5.08640 -0.74156 -1.72433
H  -4.91163  1.30678  1.01341
H  -7.38750  1.22487  0.73224
H  -8.40026 -0.12261 -1.11795
H  -6.91608 -1.38312 -2.69364
H  -4.43946 -1.27841 -2.42838
```

PPh3

```
SCF  =          -701.502629684
H(0 K)=         -701.237099
G(298 K)=       -701.284049
SCF(C6H6) =     -701.505204420
BP86(D3BJ) =    -701.583032714
Low Freq. = 23.0073cm-1, 25.2587cm-1
```

```
34
C  -0.78046  4.21297  0.67511
C  -0.01358  4.06660 -0.49293
C   0.19786  2.79152 -1.03889
C  -0.33782  1.64207 -0.41587
C  -1.10868  1.80016  0.75512
C  -1.32915  3.07854  1.29461
P   0.00027 -0.00024 -1.25088
C  -1.25337 -1.11399 -0.41619
C   1.59147 -0.52853 -0.41559
H  -1.54080  0.92001  1.24212
H  -1.93197  3.18699  2.20297
H  -0.95512  5.20848  1.09685
H   0.41165  4.94747 -0.98590
H   0.78148  2.68379 -1.96082
C  -1.00522 -1.86133  0.75444
C  -2.00245 -2.69132  1.29346
C  -3.25927 -2.78251  0.67390
C  -3.51560 -2.04449 -0.49377
C  -2.51669 -1.22402 -1.03931
H  -0.02700 -1.79616  1.24167
H  -1.79521 -3.26815  2.20153
H  -4.03435 -3.43141  1.09530
H  -4.49109 -2.11598 -0.98680
H  -2.71495 -0.66419 -1.96097
C   2.32265 -1.56257 -1.04203
C   3.53271 -2.01665 -0.49591
C   4.03930 -1.42990  0.67579
C   3.32758 -0.39205  1.29882
C   2.11021  0.05582  0.75910
H   1.94031 -2.01065 -1.96679
H   4.08583 -2.82171 -0.99161
H   4.98881 -1.77607  1.09769
H   3.72015  0.07254  2.21003
H   1.56123  0.86668  1.24871
```

IMe4

```
SCF  =          -383.432446140
H(0 K)=         -383.255338
G(298 K)=       -383.290232
SCF(C6H6) =     -383.436076820
BP86(D3BJ) =    -383.462871813
Low Freq. = 121.9546cm-1, 124.0941cm-1
```

```
21
C  -0.68843  0.64433 -0.00009
N  -1.06514 -0.71067 -0.00023
C   0.00000 -1.59189 -0.00005
N   1.06514 -0.71067  0.00006
C   0.68843  0.64433  0.00010
C  -2.44336 -1.18034  0.00015
C   2.44336 -1.18034 -0.00002
C   1.66794  1.77560  0.00006
C  -1.66794  1.77559 -0.00004
H   2.40319 -2.27846  0.00014
H   2.98833 -0.83447  0.89649
H   2.98817 -0.83473 -0.89673
H  -2.98923 -0.83223 -0.89493
H  -2.98726 -0.83697  0.89828
H  -2.40320 -2.27846 -0.00270
H   1.14013  2.74304  0.00018
H   2.32521  1.75870  0.88945
H   2.32500  1.75882 -0.88949
H  -1.14014  2.74304  0.00002
H  -2.32510  1.75882 -0.88952
H  -2.32512  1.75869  0.88942
```

8ccc

```
SCF  =          -2340.25299607
H(0 K)=         -2339.361345
G(298 K)=       -2339.456861
SCF(C6H6) =     -2340.25937375
BP86(D3BJ) =    -2340.60855419
Low Freq. = 17.5936cm-1, 20.7694cm-1
```

```
112
N   2.51531  1.71019 -2.34116
C   2.13364  1.35850 -1.04805
N   3.25807  1.71633 -0.31160
C   4.28310  2.25175 -1.10583
C   3.80664  2.25530 -2.39256
Ru  0.31414  0.59477 -0.45566
P   1.21123 -1.45314  0.41653
C   2.89508 -1.90530 -0.33503
C   3.39579  1.51786  1.12244
C   1.67722  1.58870 -3.52832
C   0.06630  2.05461  1.07271
N  -0.37347  1.94658  2.39260
C  -0.43957  3.18198  3.05367
C  -0.02606  4.12515  2.14555
N   0.26914  3.43120  0.96184
C  -0.81463  0.70401  3.01095
```

```
C   0.68762  4.11382 -0.25738
P  -2.00152  0.13128 -0.59992
C  -2.93950  1.72979 -0.96630
C  -2.64669 -0.94605 -2.02666
C  -3.15317 -0.57835  0.75525
C  -2.72746 -1.67108  1.54800
C  -3.50966 -2.18994  2.59248
C  -4.76825 -1.63400  2.86428
C  -5.22650 -0.55730  2.09020
C  -4.42582 -0.04138  1.05713
O  -1.43979 -2.18263  1.40765
C  -1.12999 -3.03815  0.35289
C   0.18213 -2.92776 -0.15740
C   0.57736 -3.86476 -1.13084
C  -0.31369 -4.84359 -1.60526
C  -1.62233 -4.90373 -1.10380
C  -2.03599 -4.00260 -0.11066
C   1.56427 -2.06414  2.19501
H   0.48173 -0.33424 -1.80976
C   0.11893  5.60754  2.28488
C  -0.88326  3.31976  4.47563
H   0.45540  3.44571 -1.09905
H   1.77038  4.33273 -0.24959
H   0.12973  5.05766 -0.36521
H  -0.57935 -0.11179  2.31492
H  -1.90269  0.72225  3.19582
H  -0.29148  0.53349  3.96614
H  -3.04680 -4.04192  0.30607
H  -2.32501 -5.65660 -1.47573
H   0.01754 -5.55454 -2.36936
H   1.59300 -3.81874 -1.53507
H  -4.79327  0.81049  0.47718
H  -6.20753 -0.11238  2.28829
H  -5.38145 -2.03880  3.67617
H  -3.10762 -3.01901  3.18292
H  -0.22947  1.69429 -1.51519
C   4.44691  2.71527 -3.66352
C   5.60597  2.67798 -0.55350
H   3.97446  0.60386  1.34496
H   3.89908  2.38571  1.57972
H   2.38210  1.41657  1.53185
H   0.85985  0.89737 -3.28423
H   1.24422  2.56513 -3.81243
H   2.27502  1.20354 -4.37091
C  -1.76278 -1.40196 -3.02151
C  -2.23046 -2.16326 -4.10650
C  -3.59268 -2.47968 -4.21334
C  -4.48530 -2.03260 -3.22440
C  -4.01653 -1.27452 -2.14100
H  -0.69973 -1.15580 -2.91577
H  -1.52304 -2.51378 -4.86642
H  -3.95860 -3.07409 -5.05790
H  -5.55154 -2.27573 -3.29450
H  -4.72649 -0.93984 -1.37827
C  -3.44262  2.02539 -2.24955
C  -4.03546  3.27091 -2.52137
C  -4.14851  4.23852 -1.51197
C  -3.65601  3.95309 -0.22690
C  -3.05002  2.71600  0.03990
H  -3.36982  1.27980 -3.04662
H  -4.41618  3.47864 -3.52770
H  -4.61848  5.20550 -1.72188
H  -3.74195  4.69685  0.57334
H  -2.65889  2.51545  1.04228
C   3.98227 -2.38772  0.42542
C   5.22211 -2.66256 -0.17882
C   5.39610 -2.47779 -1.55838
C   4.32076 -2.00447 -2.32897
C   3.08971 -1.71062 -1.72238
H   3.86498 -2.55744  1.49931
H   6.05070 -3.03281  0.43558
H   6.35946 -2.70108 -2.02991
H   4.44115 -1.85851 -3.40851
H   2.25840 -1.31445 -2.31473
C   1.46797 -3.42232  2.56949
C   1.75602 -3.83369  3.88095
C   2.15720 -2.89527  4.84609
C   2.26378 -1.54218  4.48825
C   1.96376 -1.13516  3.17700
H   1.16738 -4.16738  1.82562
H   1.67031 -4.89317  4.14786
H   2.38374 -3.21635  5.86861
H   2.57639 -0.79959  5.23147
H   2.03219 -0.07559  2.91426
H   5.51312  3.49521  0.18619
H   6.13457  1.84474 -0.05535
H   6.25591  3.04263 -1.36441
H   3.85016  3.49283 -4.17478
H   5.43971  3.14386 -3.45497
H   4.58992  1.88882 -4.38436
H  -0.11538  5.91668  3.31579
H   1.14617  5.94965  2.06119
H  -0.56146  6.16245  1.61235
H  -0.23162  2.76129  5.17317
H  -0.86502  4.37767  4.78174
H  -1.91300  2.94787  4.62899
```

8ccc’ (from IRC)

```
SCF  =           -2340.24014594
H(0 K)=          -2339.347843
G(298 K)=        -2339.441986
SCF(C6H6) =      -2340.24652044
BP86(D3BJ) =     -2340.59315247
Low Freq. = 11.0042cm-1, 23.6886cm-1

112
N   2.56115  1.13427 -2.53581
C   2.13979  1.06724 -1.20810
N   3.21893  1.63790 -0.53606
C   4.24128  2.04928 -1.40337
C   3.82209  1.73360 -2.67137
Ru  0.28580  0.48373 -0.51320
P   1.09525 -1.59023  0.44556
C   2.91267 -1.61437  0.96300
C   3.34184  1.76184  0.91081
C   1.77682  0.71017 -3.68958
C   0.30217  2.08785  0.90986
N   0.26126  2.10508  2.30466
C   0.25132  3.39717  2.84580
C   0.29536  4.25884  1.77795
N   0.32421  3.45765  0.62501
C   0.23709  0.91490  3.13716
C   0.42089  4.04531 -0.70671
P  -2.06920  0.27882 -0.47967
C  -3.04582  0.59231  1.11352
C  -2.89478  1.53765 -1.63805
C  -3.06059 -1.22820 -1.10866
C  -2.51967 -2.53363 -1.14581
C  -3.30519 -3.64934 -1.50191
C  -4.63812 -3.46677 -1.89062
C  -5.19029 -2.17626 -1.90281
C  -4.40966 -1.08207 -1.50353
O  -1.23040 -2.69137 -0.68018
C  -0.19651 -3.28081 -1.38619
C   1.07992 -2.97333 -0.85187
C   2.19004 -3.64288 -1.39264
C   2.05334 -4.51858 -2.48679
C   0.79159 -4.72498 -3.05778
C  -0.34743 -4.11260 -2.50495
C   0.36954 -2.66093  1.84958
H   0.17544 -0.51394 -1.81726
C   0.30766  5.75437  1.73520
C   0.18718  3.66433  4.31631
H   0.22062  3.24214 -1.43107
H   1.42954  4.46048 -0.88423
H  -0.32332  4.85030 -0.82381
H   0.46184  0.06351  2.48098
H  -0.75749  0.76489  3.59129
H   0.99509  0.98512  3.93578
H  -1.33249 -4.28245 -2.94448
H   0.67700 -5.37876 -3.92907
H   2.93566 -5.02363 -2.89359
H   3.18309 -3.47058 -0.96867
```

H -4.85728 -0.08362 -1.49693
H -6.23043 -2.02110 -2.20733
H -5.24506 -4.33467 -2.16954
H -2.87181 -4.65079 -1.43846
H -0.13672 1.54554 -1.64018
C 4.48961 1.93876 -3.99424
C 5.51108 2.67726 -0.92332
H 4.33717 1.41555 1.23245
H 3.19033 2.80637 1.23725
H 2.57315 1.12919 1.36638
H 0.99760 0.02730 -3.32003
H 1.29343 1.57530 -4.17911
H 2.42751 0.19965 -4.41800
C -2.44664 1.56140 -2.97846
C -3.02010 2.43374 -3.91393
C -4.04494 3.31554 -3.52628
C -4.49949 3.29997 -2.20055
C -3.93744 2.40903 -1.26590
H -1.63437 0.89131 -3.27711
H -2.66239 2.42905 -4.94978
H -4.48657 4.00444 -4.25454
H -5.30230 3.97611 -1.88539
H -4.32243 2.39816 -0.24199
C -2.90184 1.84199 1.76194
C -3.52822 2.09467 2.99262
C -4.29636 1.09612 3.61631
C -4.44034 -0.15109 2.98917
C -3.82761 -0.39798 1.74811
H -2.29635 2.62602 1.29667
H -3.41670 3.07814 3.46313
H -4.78178 1.29145 4.57855
H -5.04349 -0.93633 3.45874
H -3.96404 -1.37243 1.26983
C 3.30336 -1.71704 2.31807
C 4.64907 -1.58976 2.69987
C 5.64303 -1.36483 1.73357
C 5.27244 -1.25225 0.38289
C 3.92394 -1.36035 0.00507
H 2.54844 -1.91150 3.08610
H 4.92091 -1.67944 3.75774
H 6.69455 -1.28112 2.02894
H 6.03462 -1.07627 -0.38469
H 3.65253 -1.23459 -1.04719
C 0.95395 -3.88858 2.23247
C 0.37113 -4.67896 3.23489
C -0.81401 -4.26045 3.86506
C -1.41389 -3.05257 3.47863
C -0.82417 -2.26231 2.47686
H 1.86966 -4.23174 1.73899
H 0.83996 -5.62743 3.52057
H -1.26978 -4.87906 4.64604
H -2.34633 -2.72100 3.94882
H -1.30783 -1.33258 2.16138
H 5.32706 3.58785 -0.32419
H 6.10367 1.98789 -0.29355
H 6.14074 2.96601 -1.77968
H 3.86642 2.53257 -4.68773
H 5.44107 2.47704 -3.85980
H 4.72068 0.98393 -4.50244
H 0.30721 6.16392 2.75767
H 1.20166 6.15105 1.21970
H -0.57567 6.16727 1.21343
H 1.06558 3.26360 4.85571
H 0.15041 4.74853 4.50759
H -0.71010 3.21281 4.77757

TS(8-2)ccc

SCF = -2340.20460858
H(0 K)= -2339.315269
G(298 K)= -2339.409267
SCF(C6H6) = -2340.21165727
BP86(D3BJ) = -2340.55856003
Low Freq. = -539.9240cm-1, 14.2400cm-1

112
N 2.38563 1.52509 -2.37618
C 1.94053 1.38028 -1.06782
N 2.94176 2.02247 -0.34587
C 3.95536 2.53558 -1.16993
C 3.60349 2.21407 -2.45748
Ru 0.13737 0.57343 -0.43853
P 1.32067 -1.48055 0.48367
C 3.01315 -1.33303 1.30293
C 2.96345 2.17206 1.10396
C 1.68436 1.03864 -3.56036
C -0.09560 2.08179 0.96340
N -0.12908 2.02522 2.36177
C -0.33044 3.27931 2.95743
C -0.43763 4.17983 1.92829
N -0.28911 3.44692 0.73947
C 0.16416 0.83386 3.14161
C -0.29601 4.10670 -0.56104
P -2.14987 -0.00078 -0.39956
C -3.03826 -0.77986 1.07002
C -3.33077 1.44418 -0.75077
C -2.69892 -1.15440 -1.77859
C -1.87276 -2.24529 -2.15100
C -2.28459 -3.14455 -3.15880
C -3.51161 -2.96570 -3.80923
C -4.33925 -1.88771 -3.45352
C -3.93449 -0.99903 -2.44702
O -0.70447 -2.50318 -1.47022
C 0.57154 -2.01369 -2.05361
C 1.68490 -2.26113 -1.15138
C 2.88307 -2.79490 -1.62565
C 3.07607 -3.07018 -2.99893
C 2.02835 -2.78055 -3.89015
C 0.79539 -2.28371 -3.44658
C 0.66477 -2.93408 1.50037
H 0.26133 -0.61891 -1.78886
C -0.68998 5.65405 1.94727
C -0.41105 3.47091 4.43867
H -0.09579 3.33564 -1.31659
H 0.48529 4.88599 -0.59998
H -1.27835 4.56631 -0.76198
H 0.30919 0.01566 2.42159
H -0.66768 0.58008 3.81926
H 1.08532 0.96737 3.73645
H -0.01550 -2.11117 -4.16093
H 2.15676 -2.97771 -4.96211
H 4.02623 -3.47780 -3.35604
H 3.70924 -2.95498 -0.92342
H -4.59503 -0.17362 -2.16579
H -5.30552 -1.74419 -3.94856
H -3.82545 -3.67245 -4.58518
H -1.62675 -3.98459 -3.39847
H -0.44050 1.57764 -1.53918
C 4.29920 2.47906 -3.75454
C 5.15113 3.26119 -0.64017
H 3.99685 2.07938 1.47062
H 2.54734 3.14712 1.41441
H 2.36069 1.36759 1.53927
H 0.68582 0.71381 -3.23766
H 1.59176 1.84966 -4.30296
H 2.21419 0.18316 -4.00917
C -3.05080 2.27861 -1.85611
C -3.90065 3.34168 -2.19642
C -5.04897 3.60335 -1.42944
C -5.34384 2.77917 -0.33413
C -4.49936 1.70310 -0.00477
H -2.15161 2.08569 -2.45034
H -3.66419 3.96732 -3.06457
H -5.71037 4.43696 -1.68940
H -6.24143 2.96244 0.26701
H -4.76410 1.06039 0.83911
C -3.05434 -0.07453 2.29575
C -3.67151 -0.61952 3.43259
C -4.27036 -1.89040 3.36890
C -4.25093 -2.60253 2.15988
C -3.64525 -2.05040 1.01801
H -2.59280 0.91767 2.34949
H -3.68988 -0.05004 4.36889
H -4.75164 -2.31809 4.25501

```
H  -4.71291 -3.59388  2.09905
H  -3.64645 -2.61399  0.08063
C   3.17691 -1.59560  2.68201
C   4.40850 -1.37572  3.32007
C   5.50965 -0.89991  2.58990
C   5.36342 -0.63938  1.21707
C   4.12695 -0.84284  0.58225
H   2.33648 -1.99502  3.25951
H   4.51054 -1.59370  4.38916
H   6.47476 -0.74278  3.08359
H   6.21873 -0.28300  0.63139
H   4.02658 -0.62638 -0.48497
C   1.24387 -4.21336  1.34971
C   0.79825 -5.29644  2.12014
C  -0.23615 -5.11955  3.05595
C  -0.82666 -3.85661  3.20437
C  -0.38133 -2.77113  2.42776
H   2.03787 -4.36196  0.61070
H   1.25685 -6.28267  1.98695
H  -0.58518 -5.96616  3.65738
H  -1.64799 -3.70836  3.91323
H  -0.87610 -1.80189  2.53237
H   4.87346  4.15846 -0.05707
H   5.76949  2.62277  0.01799
H   5.79136  3.59342 -1.47237
H   3.69199  3.10285 -4.43646
H   5.24707  3.01089 -3.57726
H   4.53707  1.54449 -4.29428
H  -0.77973  6.01146  2.98506
H   0.12624  6.22676  1.46951
H  -1.62501  5.92183  1.42180
H   0.51654  3.15901  4.95330
H  -0.57936  4.53274  4.67748
H  -1.24035  2.89487  4.88935
```

2ccc (from IRC)

```
SCF  =           -2340.29147879
H(0 K)=          -2339.396594
G(298 K)=        -2339.493025
SCF(C6H6) =      -2340.29921118
BP86(D3BJ) =     -2340.64148114
Low Freq. = 4.5520cm-1, 14.3797cm-1
```

```
112
N  -1.83008  2.02092  2.42616
C  -1.70914  1.59994  1.10181
N  -2.90257  2.05067  0.54528
C  -3.73278  2.68400  1.48151
C  -3.05381  2.66186  2.67330
Ru -0.06477  0.60566  0.33214
P  -1.38275 -1.37190 -0.43816
C  -2.91391 -1.14274 -1.53174
C  -3.24410  2.00420 -0.86897
C  -0.79796  1.93008  3.46073
C   0.06455  1.71588 -1.35614
N   0.44275  1.33074 -2.64921
C   0.48541  2.40550 -3.55109
C   0.13587  3.52351 -2.83999
N  -0.10549  3.09910 -1.52243
C   0.86300 -0.01045 -3.02785
C  -0.43941  4.04495 -0.46593
P   2.28879  0.06684  0.49723
C   3.38118 -1.27581 -0.28779
C   3.52941  1.48875  0.34341
C   2.35000 -0.39466  2.28536
C   1.06596 -0.68880  2.85380
C   1.04476 -1.19646  4.19464
C   2.22066 -1.33517  4.93074
C   3.47434 -0.99222  4.37574
C   3.52568 -0.53491  3.05331
O  -0.06164 -0.49644  2.20589
C  -2.73231 -1.61713  2.05820
C  -2.18295 -2.33437  0.97388
C  -2.31363 -3.73833  0.95686
C  -2.97708 -4.40950  1.99832
C  -3.52920 -3.68664  3.06642
C  -3.40774 -2.28749  3.08985
C  -0.58908 -2.81465 -1.36686
H  -2.61372 -0.53229  2.09581
C   0.01348  4.95026 -3.27165
C   0.86573  2.24169 -4.98810
H  -0.29119  3.54081  0.49769
H  -1.48760  4.38729 -0.53847
H   0.22096  4.92595 -0.52846
H   0.89543 -0.62613 -2.11944
H   1.86596  0.01741 -3.48414
H   0.16883 -0.47069 -3.75331
H  -3.82756 -1.71392  3.92375
H  -4.04764 -4.20967  3.87749
H  -3.06343 -5.50127  1.96832
H  -1.89863 -4.31635  0.12641
H   4.49360 -0.27703  2.60801
H   4.39110 -1.08436  4.96660
H   2.16789 -1.71032  5.96018
H   0.07163 -1.46148  4.62247
H   0.68674  1.82002  1.08338
C  -3.45036  3.17514  4.02145
C  -5.08383  3.22249  1.13288
H  -4.14354  1.39316 -1.04549
H  -3.42450  3.02626 -1.24877
H  -2.39603  1.56340 -1.40179
H   0.17552  1.82085  2.96684
H  -0.81282  2.85631  4.05842
H  -0.95517  1.06113  4.11664
C   3.08343  2.82361  0.26329
C   3.99477  3.89106  0.21280
C   5.37600  3.64516  0.24432
C   5.83632  2.32134  0.32155
C   4.92396  1.25502  0.36435
H   2.00771  3.01369  0.25036
H   3.62176  4.91965  0.14879
H   6.08800  4.47682  0.20698
H   6.91168  2.11296  0.34702
H   5.30523  0.23051  0.40429
C   3.88017 -1.10293 -1.59831
C   4.67633 -2.08435 -2.20759
C   4.99631 -3.26354 -1.51365
C   4.51152 -3.44761 -0.20911
C   3.70742 -2.46574  0.39656
H   3.67740 -0.16906 -2.13403
H   5.05928 -1.92218 -3.22149
H   5.62561 -4.02704 -1.98345
H   4.76765 -4.35506  0.34969
H   3.34884 -2.61263  1.42038
C  -2.76533 -0.56868 -2.81525
C  -3.87190 -0.35329 -3.65049
C  -5.16312 -0.69388 -3.21232
C  -5.32823 -1.25984 -1.93888
C  -4.21492 -1.48883 -1.11010
H  -1.77132 -0.27188 -3.16119
H  -3.72568  0.08736 -4.64301
H  -6.03001 -0.52198 -3.85926
H  -6.32810 -1.53611 -1.58587
H  -4.36315 -1.94204 -0.12618
C  -1.12473 -3.41379 -2.52678
C  -0.47274 -4.49834 -3.13882
C   0.71491 -5.00943 -2.59413
C   1.24975 -4.42767 -1.43307
C   0.60652 -3.33661 -0.82983
H  -2.06094 -3.04389 -2.95368
H  -0.90410 -4.94819 -4.04021
H   1.22137 -5.85592 -3.07039
H   2.18081 -4.80704 -1.00147
H   1.03234 -2.88620  0.07202
H  -5.03389  4.03295  0.38204
H  -5.74875  2.44056  0.72268
H  -5.57126  3.63615  2.02944
H  -2.80898  4.00861  4.36316
H  -4.48584  3.54900  3.99381
H  -3.39954  2.38779  4.79474
H   0.18320  5.03389 -4.35648
H  -0.98783  5.36818 -3.05951
H   0.75195  5.60617 -2.77361
```

H   0.21809 1.51478 -5.51187
H   0.77987 3.20357 -5.51732
H   1.90809 1.89214 -5.10811

2ccc (lowest conf)

SCF =   -2340.30333676
H(0 K)=   -2339.408025
G(298 K)=   -2339.502086
SCF(C6H6) =   -2340.31030980
BP86(D3BJ) =   -2340.65899210
Low Freq. = 16.5157cm-1, 20.5151cm-1

112
N -0.28162 -3.29959 0.73542
C -0.14524 -1.91772 0.93640
N -0.01191 -1.85288 2.32570
C -0.06857 -3.10944 2.94182
C -0.24632 -4.02653 1.93728
Ru -0.17575 -0.46591 -0.47728
C -2.00370 -1.21431 -1.11677
N -2.37332 -1.49267 -2.42572
C -3.61629 -2.13623 -2.50489
C -4.06970 -2.26795 -1.21604
N -3.08376 -1.70455 -0.39315
C -1.53040 -1.28861 -3.60428
C -3.21380 -1.70080 1.05836
C   0.19851 -0.62965 3.08152
C -0.48215 -3.95878 -0.55082
P -1.20530 1.57396 0.44548
C -0.51103 2.80325 1.71657
C -1.31181 2.60673 -1.09127
C -0.68812 2.02946 -2.24573
C -0.72045 2.78763 -3.46068
C -1.34112 4.03408 -3.51616
C -1.95340 4.59402 -2.37205
C -1.92664 3.87556 -1.17193
O -0.08431 0.85744 -2.21914
C   2.35556 2.67451 -0.87166
C   3.09015 1.48265 -0.73739
C   4.49151 1.52278 -0.92155
C   5.13992 2.73176 -1.21087
C   4.39602 3.91822 -1.33819
C   3.00422 3.88525 -1.17297
P   2.21316 -0.15099 -0.37277
C   3.06943 -1.15288 -1.72393
C -2.99779 1.55082 1.02732
C   3.13341 -0.71219 1.16829
H   1.26969 2.66182 -0.75777
H   0.35990 -1.56397 -1.51394
H   2.41093 4.79899 -1.28140
H   4.90170 4.86130 -1.57283
H   6.22709 2.74562 -1.34644
H   5.07840 0.60120 -0.84733
H -2.39543 4.30419 -0.27923
H -2.43961 5.57347 -2.42134
H -1.34921 4.58812 -4.46270
H -0.23315 2.35716 -4.34239
C -5.34978 -2.82623 -0.68057
C -4.24618 -2.53577 -3.80137
C -0.37465 -5.51614 1.99797
C   0.07953 -3.29219 4.41875
H -0.79274 -0.50780 -3.36617
H -1.02185 -2.22978 -3.88234
H -2.15785 -0.95908 -4.44775
H -2.41431 -1.07695 1.46854
H -4.18905 -1.28105 1.35145
H -3.11570 -2.72464 1.46256
H -1.55228 -4.16284 -0.73832
H -0.10278 -3.28725 -1.33393
H   0.07266 -4.91033 -0.57139
H   0.03299 0.20959 2.39529
H -0.51392 -0.56708 3.92178
H   1.22856 -0.57764 3.47378
C   2.57200 -0.97238 -3.03497
C   3.17004 -1.63560 -4.11580
C   4.26168 -2.49765 -3.90607
C   4.76032 -2.67850 -2.60832
C   4.17367 -2.00207 -1.52189
H   1.71778 -0.30249 -3.19140
H   2.78081 -1.48041 -5.12838
H   4.72237 -3.02039 -4.75141
H   5.61425 -3.34276 -2.43373
H   4.58385 -2.14342 -0.51709
C   3.82034 0.18948 2.01057
C   4.40394 -0.24725 3.21212
C   4.31996 -1.59641 3.59100
C   3.64563 -2.50624 2.75832
C   3.05162 -2.06731 1.56446
H   3.91323 1.24003 1.71825
H   4.93366 0.47053 3.84813
H   4.78260 -1.93860 4.52299
H   3.57948 -3.56354 3.03756
H   2.51652 -2.78428 0.93373
C -1.26652 3.87259 2.25057
C -0.68854 4.78888 3.14276
C   0.65595 4.65231 3.52663
C   1.41579 3.59135 3.01200
C   0.83436 2.67784 2.11708
H -2.32130 3.98424 1.98136
H -1.29294 5.61169 3.54070
H   1.10527 5.36635 4.22526
H   2.46413 3.47040 3.30574
H   1.43430 1.85262 1.72011
C -3.32526 1.44160 2.39813
C -4.65957 1.31124 2.81455
C -5.69552 1.27683 1.86499
C -5.38292 1.38146 0.49888
C -4.04722 1.51810 0.08445
H -2.52933 1.47577 3.15022
H -4.89084 1.24170 3.88348
H -6.73777 1.18144 2.18831
H -6.18293 1.37042 -0.24998
H -3.81174 1.61492 -0.98026
H -5.18126 -3.64015 0.04810
H -5.95497 -2.05329 -0.17185
H -5.95826 -3.23711 -1.50133
H -5.19747 -3.05844 -3.61521
H -4.46822 -1.66334 -4.44350
H -3.60163 -3.21576 -4.38720
H -0.37039 -5.85289 3.04650
H -1.31378 -5.87533 1.53866
H   0.45627 -6.03419 1.48333
H   0.02107 -4.36050 4.67994
H   1.05191 -2.91148 4.78096
H -0.70989 -2.76868 4.98921

8cct (lowest conf)

SCF =   -2340.24984064
H(0 K)=   -2339.358599
G(298 K)=   -2339.451359
SCF(C6H6) =   -2340.25608673
BP86(D3BJ) =   -2340.61004571
Low Freq. = 14.4994cm-1, 20.7437cm-1

112
N -2.97686 1.69261 -1.25213
C -2.05736 1.59564 -0.21065
N -2.64216 2.40265 0.76178
C -3.85773 2.96008 0.34334
C -4.07318 2.50610 -0.93308
Ru -0.23603 0.59303 -0.11743
C   0.54182 2.51579 -0.02903
N   1.18580 3.17365 1.01635
C   1.47618 4.51175 0.71615
C   0.99653 4.73753 -0.54928
N   0.44060 3.52591 -0.98226
C   1.55636 2.57024 2.28805
C -0.14191 3.36526 -2.30704
C -2.08355 2.67514 2.07930
C -2.84699 1.04303 -2.54821
P   1.97112 -0.28629 -0.09868
C   2.69723 -1.06666 1.47619

```
C   2.64227 -1.60966 -1.30389
C   1.93111 -2.81549 -1.49392
C   2.38426 -3.83043 -2.35142
C   3.59681 -3.66873 -3.03697
C   4.33262 -2.48748 -2.86403
C   3.85746 -1.47633 -2.01120
O   0.67156 -2.95220 -0.92991
C   0.46264 -3.43115  0.35363
C  -0.66862 -2.87646  0.99302
C  -1.03869 -3.41066  2.23999
C  -0.27681 -4.42766  2.84371
C   0.86845 -4.92496  2.20304
C   1.24323 -4.43472  0.94181
P  -1.45672 -1.42386  0.07841
C  -2.11683 -2.43085 -1.39012
C   3.26867  1.03848 -0.46949
C  -3.03385 -1.17638  1.08603
H  -0.10946  0.55420 -1.80950
H  -0.34094  0.55206  1.56621
H   2.12202 -4.82117  0.41722
H   1.47051 -5.70718  2.67725
H  -0.57909 -4.82633  3.81778
H  -1.92545 -3.02506  2.75194
H   4.44491 -0.56117 -1.89826
H   5.27970 -2.34433 -3.39506
H   3.95649 -4.45725 -3.70623
H   1.76579 -4.72403 -2.47875
C   1.01673  5.97321 -1.39146
C   2.20200  5.41665  1.65956
C  -5.20963  2.74685 -1.87505
C  -4.68849  3.84794  1.21383
H   2.64624  2.40812  2.34446
H   1.24453  3.22022  3.12352
H   1.03385  1.60342  2.34518
H   0.50539  3.84078 -3.06299
H  -0.21444  2.27917 -2.48499
H  -1.14821  3.81723 -2.36039
H  -1.80645  0.68891 -2.61692
H  -3.53172  0.18283 -2.63783
H  -3.06478  1.76229 -3.35609
H  -1.65852  3.69364  2.12630
H  -2.86691  2.57088  2.84745
H  -1.28749  1.93041  2.24045
C   1.92793 -1.04787  2.65431
C   2.42599 -1.59946  3.84694
C   3.70359 -2.17885  3.87692
C   4.47644 -2.21527  2.70376
C   3.97450 -1.67042  1.51158
H   0.92400 -0.60586  2.60518
H   1.80821 -1.58430  4.75180
H   4.09319 -2.61024  4.80569
H   5.47114 -2.67509  2.71426
H   4.58071 -1.72114  0.60094
C   4.32864  1.38193  0.39257
C   5.23517  2.40228  0.04742
C   5.10334  3.08399 -1.17096
C   4.05206  2.74557 -2.04161
C   3.13923  1.74055 -1.69009
H   4.45980  0.84377  1.33642
H   6.05316  2.65262  0.73248
H   5.81497  3.87087 -1.44387
H   3.94346  3.26684 -2.99948
H   2.31039  1.48540 -2.35950
C  -2.95037 -3.55752 -1.22065
C  -3.37190 -4.30884 -2.32969
C  -2.95113 -3.95406 -3.62265
C  -2.09610 -2.85385 -3.79696
C  -1.68023 -2.10103 -2.68626
H  -3.26591 -3.85686 -0.21514
H  -4.02312 -5.17776 -2.18216
H  -3.27745 -4.54220 -4.48759
H  -1.74158 -2.58684 -4.79911
H  -0.98136 -1.26240 -2.79766
C  -2.91501 -0.74358  2.42684
C  -4.05153 -0.48759  3.20941
C  -5.33670 -0.62495  2.65721
C  -5.46912 -1.02064  1.31827
C  -4.32960 -1.30010  0.54279
H  -1.91540 -0.58281  2.84386
H  -3.93164 -0.17295  4.25249
H  -6.22513 -0.42145  3.26504
H  -6.46429 -1.12197  0.87072
H  -4.45690 -1.62281 -0.49453
H   3.21106  5.03414  1.89911
H   2.32435  6.41498  1.21083
H   1.66596  5.54813  2.61784
H   0.00304  6.27642 -1.71168
H   1.44975  6.81222 -0.82433
H   1.62387  5.84767 -2.30727
H  -5.96751  3.38891 -1.39935
H  -4.88844  3.24993 -2.80622
H  -5.70950  1.80631 -2.17047
H  -5.05571  3.32091  2.11388
H  -4.12927  4.73649  1.55971
H  -5.57018  4.20652  0.65969
```

8cct (from IRC)

```
SCF  =           -2340.24915289
H(0 K)=          -2339.357411
G(298 K)=        -2339.449754
SCF(C6H6) =      -2340.25528816
BP86(D3BJ) =     -2340.60901768
Low Freq. = 14.0192cm-1, 22.5571cm-1
```

```
112
N  -2.27662  2.38695  1.30035
C  -1.54931  2.02919  0.16580
N  -2.04228  2.91982 -0.78492
C  -3.02264  3.77747 -0.26743
C  -3.17320  3.43603  1.05367
Ru -0.02648  0.62723 -0.04390
P  -1.70925 -1.04624 -0.18982
C  -3.19200 -0.94893  0.98901
C  -1.58697  2.99901 -2.16657
C  -2.17140  1.77271  2.62067
C   1.19854  2.28966 -0.23846
N   1.92361  2.74548 -1.33829
C   2.53709  3.98328 -1.10400
C   2.19817  4.35121  0.17386
N   1.39789  3.31801  0.68038
C   2.10160  2.02976 -2.59552
C   0.89209  3.31829  2.04596
P   1.84193 -0.79558  0.13873
C   2.13812 -2.22084 -1.06614
C   3.51510  0.07500 -0.02790
C   2.17524 -1.66945  1.80265
C   1.12073 -2.24958  2.54778
C   1.30610 -2.74058  3.84970
C   2.58166 -2.71781  4.43205
C   3.66171 -2.21122  3.69581
C   3.45305 -1.69289  2.40646
O  -0.16821 -2.30415  2.02540
C  -0.42017 -3.26647  1.04797
C  -1.24715 -2.87551 -0.02647
C  -1.56268 -3.86384 -0.98277
C  -1.08036 -5.17726 -0.87082
C  -0.25866 -5.53270  0.20884
C   0.07423 -4.57503  1.17575
C  -2.71875 -1.16354 -1.80140
H   0.08646  0.59807  1.63001
C   2.54871  5.57301  0.96224
C   3.39932  4.66566 -2.11732
H   1.69156  3.61985  2.74422
H   0.56247  2.28687  2.25587
H   0.03901  4.01036  2.15876
H   3.13768  1.66439 -2.69188
H   1.86730  2.69195 -3.44702
H   1.40169  1.17896 -2.57534
H   0.71210 -4.82828  2.02756
H   0.12513 -6.55406  0.30324
H  -1.34117 -5.91702 -1.63462
H  -2.18392 -3.59836 -1.84148
```

```
H   4.30546 -1.27597  1.86379
H   4.66952 -2.20476  4.12423
H   2.72817 -3.10540  5.44567
H   0.43933 -3.14465  4.38185
H  -0.07891  0.60316 -1.73015
C  -4.08872  3.97972  2.10387
C  -3.70684  4.82131 -1.09166
H  -0.91196  2.14040 -2.31723
H  -2.44454  2.94009 -2.85786
H  -1.04149  3.94244 -2.34645
H  -1.38093  1.00779  2.55403
H  -1.89673  2.53503  3.37156
H  -3.12773  1.30706  2.90794
C  -3.28335 -1.71225  2.17109
C  -4.37183 -1.55843  3.04675
C  -5.39242 -0.63853  2.75849
C  -5.31438  0.12813  1.58286
C  -4.22456 -0.02409  0.71076
H  -2.49816 -2.43003  2.41902
H  -4.42302 -2.16855  3.95555
H  -6.24454 -0.52576  3.43760
H  -6.10938  0.84112  1.33604
H  -4.18465  0.57529 -0.20440
C  -2.21508 -0.61450 -2.99453
C  -2.92833 -0.72426 -4.20128
C  -4.16339 -1.38880 -4.23398
C  -4.67706 -1.94830 -3.05155
C  -3.96156 -1.83836 -1.84885
H  -1.25443 -0.08518 -2.94200
H  -2.51582 -0.28599 -5.11748
H  -4.72336 -1.47253 -5.17203
H  -5.63865 -2.47356 -3.06371
H  -4.37453 -2.28377 -0.93838
C   1.52860 -2.13227 -2.33274
C   1.77892 -3.10360 -3.31659
C   2.63478 -4.18138 -3.03952
C   3.23600 -4.28536 -1.77366
C   2.99093 -3.31093 -0.79315
H   0.85064 -1.29060 -2.52287
H   1.29671 -3.02204 -4.29715
H   2.82788 -4.94260 -3.80347
H   3.89904 -5.12825 -1.54827
H   3.46752 -3.39818  0.18898
C   3.83813  1.09615  0.89561
C   5.03664  1.81579  0.78554
C   5.93787  1.53482 -0.25699
C   5.63007  0.52344 -1.17896
C   4.43021 -0.20336 -1.06313
H   3.14242  1.31725  1.71102
H   5.27017  2.59644  1.51792
H   6.87535  2.09507 -0.34274
H   6.32804  0.28685 -1.99002
H   4.21121 -0.99658 -1.78400
H   3.14720  5.33840  1.86228
H   3.14293  6.26560  0.34556
H   1.65064  6.11960  1.30442
H   2.85566  4.88775 -3.05422
H   3.77271  5.62172 -1.71780
H   4.27777  4.05116 -2.38637
H  -4.76256  3.19874  2.50086
H  -3.53804  4.40158  2.96484
H  -4.71554  4.78316  1.68587
H  -4.41875  5.38954 -0.47248
H  -2.99198  5.54512 -1.52476
H  -4.27555  4.38451 -1.93346
```

TS(8-2)cct

```
SCF  =          -2340.20937002
H(0 K)=         -2339.320714
G(298 K)=       -2339.412192
Low Freq. = -762.3884cm-1, 22.2082cm-1

112
N  -1.69302  2.92937  0.87523
C  -1.02146  2.34239 -0.19099
N  -1.21286  3.27563 -1.20541
C  -1.96384  4.38278 -0.78346
C  -2.27122  4.15984  0.53616
Ru  0.10393  0.60607 -0.21371
P  -1.87169 -0.72971 -0.20278
C  -3.44710 -0.01397  0.56298
C  -0.69573  3.15148 -2.56205
C  -1.83867  2.34480  2.20381
C   1.73411  1.86827 -0.39028
N   2.59192  2.06918 -1.46548
C   3.52406  3.08838 -1.22626
C   3.26076  3.56928  0.03250
N   2.18301  2.81686  0.52211
C   2.61271  1.27238 -2.68626
C   1.62922  3.00550  1.85719
P   1.55279 -1.26826  0.13919
C   1.26917 -2.99342 -0.53855
C   3.38053 -1.10221 -0.33151
C   1.63504 -1.39031  1.99185
C   0.55331 -0.67250  2.65430
C   0.79279 -0.23307  3.99822
C   1.99354 -0.51632  4.65560
C   3.00189 -1.28113  4.03813
C   2.80923 -1.68439  2.70279
O  -0.86461 -1.07676  2.50871
C  -1.14242 -2.31899  1.97811
C  -1.76192 -2.36933  0.70568
C  -2.16159 -3.62533  0.20624
C  -1.94787 -4.79516  0.95145
C  -1.32268 -4.72797  2.20833
C  -0.91452 -3.49082  2.72463
C  -2.65320 -1.25115 -1.84567
H   0.31099  0.45128  1.60528
C   3.91815  4.65882  0.81861
C   4.57949  3.46667 -2.21559
H   2.44395  3.10564  2.59250
H   1.02654  2.11285  2.08668
H   0.99018  3.90519  1.90211
H   3.52833  0.65970 -2.73352
H   2.56466  1.92857 -3.57274
H   1.72742  0.61881 -2.65485
H  -0.43802 -3.40644  3.70557
H  -1.15736 -5.64102  2.79069
H  -2.26429 -5.76134  0.54512
H  -2.62921 -3.69317 -0.78004
H   3.63259 -2.17543  2.17073
H   3.93066 -1.52121  4.56387
H   2.12378 -0.17257  5.68926
H  -0.02047  0.28057  4.52255
H   0.07511  0.53067 -1.84584
C  -3.07519  4.96809  1.50382
C  -2.30644  5.52510 -1.68608
H  -0.30736  2.12505 -2.65631
H  -1.50139  3.32346 -3.29581
H   0.11711  3.87658 -2.74432
H  -1.32577  1.37227  2.19810
H  -1.38257  3.00270  2.96470
H  -2.90416  2.19611  2.44229
C  -4.01031 -0.48933  1.76387
C  -5.16484  0.10720  2.30051
C  -5.77549  1.18746  1.64405
C  -5.22766  1.66296  0.43934
C  -4.07488  1.06688 -0.09481
H  -3.54935 -1.33302  2.28502
H  -5.58876 -0.28024  3.23363
H  -6.67775  1.64870  2.06035
H  -5.70242  2.49624 -0.09071
H  -3.66024  1.44249 -1.03646
C  -1.96491 -1.03504 -3.05431
C  -2.53654 -1.40295 -4.28476
C  -3.80893 -1.99341 -4.32278
C  -4.51073 -2.20646 -3.12389
C  -3.94018 -1.83391 -1.89679
H  -0.98358 -0.54582 -3.00534
H  -1.98670 -1.22199 -5.21547
H  -4.25711 -2.27952 -5.28067
H  -5.50886 -2.65788 -3.14373
H  -4.50484 -1.98753 -0.97109
```

```
C   0.78593 -3.13549 -1.85548
C   0.66117 -4.40768 -2.43796
C   1.01531 -5.55266 -1.70599
C   1.48683 -5.41945 -0.38946
C   1.61543 -4.14770  0.19139
H   0.49915 -2.24124 -2.41849
H   0.28140 -4.50269 -3.46116
H   0.91776 -6.54616 -2.15722
H   1.75566 -6.30989  0.18962
H   1.97548 -4.04738  1.22035
C   4.16858 -0.11407  0.30091
C   5.50399  0.09463 -0.07530
C   6.08235 -0.67895 -1.09710
C   5.31252 -1.66656 -1.73045
C   3.97499 -1.87684 -1.35029
H   3.73322  0.48900  1.10290
H   6.09577  0.86182  0.43614
H   7.12662 -0.51992 -1.38759
H   5.75271 -2.28615 -2.51983
H   3.39411 -2.65708 -1.85072
H   4.39187  4.28344  1.74471
H   4.70700  5.13772  0.21753
H   3.20271  5.44722  1.11645
H   4.15332  3.80047 -3.17962
H   5.19292  4.29239 -1.82212
H   5.25690  2.62038 -2.43126
H  -3.96800  4.41680  1.85116
H  -2.49389  5.24799  2.40145
H  -3.42107  5.89984  1.02929
H  -2.87000  6.29174 -1.13153
H  -1.40563  6.01191 -2.10297
H  -2.93155  5.21134 -2.54274
```

2cct (from IRC)

```
SCF  =          -2340.30176695
H(0 K)=         -2339.406466
G(298 K)=       -2339.501417
SCF(C6H6) =     -2340.30851034
BP86(D3BJ) =    -2340.65831393
Low Freq. = 15.6813cm-1, 20.5404cm-1
```

```
112
N  -2.15877  2.76327  0.70311
C  -1.66482  1.85533 -0.21877
N  -2.39137  2.16883 -1.36199
C  -3.28715  3.22751 -1.15099
C  -3.13541  3.60887  0.15823
Ru -0.12168  0.49689  0.01149
P  -1.73812 -1.22792  0.08985
C  -3.55487 -0.69641  0.10088
C  -2.30598  1.48274 -2.64448
C  -1.73269  2.84413  2.09677
C   1.09416  2.21199  0.17301
N   1.40788  3.11662 -0.83689
C   2.18906  4.18635 -0.38138
C   2.38458  3.97778  0.96042
N   1.71286  2.78726  1.27587
C   1.01805  3.00486 -2.23676
C   1.68761  2.26759  2.64081
P   1.91485 -0.75765 -0.21144
C   2.01950 -2.59921 -0.64247
C   2.91776  0.01965 -1.60155
C   3.20270 -0.88535  1.17202
C   2.73732 -0.86982  2.50233
C   3.62520 -1.08922  3.57088
C   4.98673 -1.32056  3.32456
C   5.45692 -1.34916  2.00016
C   4.57048 -1.14514  0.93215
O  -0.37668  0.12670  2.28482
C  -0.89887 -1.01675  2.69270
C  -1.56593 -1.92621  1.79920
C  -2.07192 -3.15351  2.27468
C  -1.94180 -3.51947  3.62106
C  -1.31498 -2.62508  4.51392
C  -0.80890 -1.40314  4.06819
C  -1.87363 -2.64884 -1.12811
H   1.67529 -0.68404  2.69196
C   3.13660  4.77908  1.97478
C   2.66773  5.28188 -1.27967
H   2.69651  1.94816  2.95128
H   0.99755  1.41221  2.68773
H   1.33771  3.05581  3.33057
H   1.91159  3.01896 -2.88261
H   0.34520  3.83360 -2.51979
H   0.49991  2.04255 -2.34712
H  -0.31759 -0.71010  4.76081
H  -1.21583 -2.89337  5.57270
H  -2.32427 -4.48323  3.97176
H  -2.55916 -3.84394  1.57861
H   4.94625 -1.20026 -0.09459
H   6.51598 -1.54357  1.79658
H   5.67835 -1.48917  4.15729
H   3.24494 -1.08233  4.59838
H   0.02490  0.54142 -1.57340
C  -3.82924  4.66940  0.95168
C  -4.21073  3.73022 -2.21364
H  -1.57948  0.66582 -2.53286
H  -3.29084  1.07499 -2.92505
H  -1.96311  2.17316 -3.43569
H  -1.22977  1.89938  2.35974
H  -1.04836  3.69785  2.24990
H  -2.61509  2.97986  2.74357
C  -4.11545 -0.16717  1.28293
C  -5.44309  0.28909  1.30728
C  -6.23679  0.22508  0.15010
C  -5.69161 -0.30218 -1.03143
C  -4.36221 -0.75793 -1.05476
H  -3.51183 -0.13491  2.19593
H  -5.86305  0.68265  2.23987
H  -7.27582  0.57139  0.17258
H  -6.30589 -0.37512 -1.93633
H  -3.95853 -1.18917 -1.97680
C  -0.98289 -2.67069 -2.21919
C  -1.05336 -3.68530 -3.18799
C  -2.02192 -4.69474 -3.07796
C  -2.93076 -4.67368 -2.00646
C  -2.86488 -3.65307 -1.04518
H  -0.23466 -1.87479 -2.29394
H  -0.34745 -3.68890 -4.02540
H  -2.07693 -5.49006 -3.82924
H  -3.70163 -5.44784 -1.92418
H  -3.61123 -3.62187 -0.24533
C   1.25053 -3.47930  0.14850
C   1.34293 -4.86798 -0.02689
C   2.19845 -5.40220 -1.00372
C   2.97153 -4.53630 -1.79217
C   2.89102 -3.14536 -1.60739
H   0.57268 -3.07759  0.90994
H   0.73621 -5.53068  0.59914
H   2.26521 -6.48615 -1.14692
H   3.65004 -4.94135 -2.55157
H   3.51805 -2.48968 -2.21880
C   3.91742  0.98186 -1.34765
C   4.57583  1.63657 -2.40374
C   4.24809  1.34061 -3.73647
C   3.24372  0.39434 -4.00411
C   2.58084 -0.24907 -2.94772
H   4.18745  1.22409 -0.31535
H   5.35491  2.37372 -2.17923
H   4.77034  1.84027 -4.55950
H   2.97376  0.15581 -5.03885
H   1.79133 -0.97329 -3.17319
H   3.93932  4.19175  2.45664
H   3.60586  5.65307  1.49656
H   2.48198  5.15541  2.78267
H   1.83223  5.82623 -1.75708
H   3.25552  6.01550 -0.70591
H   3.31292  4.89816 -2.09111
H  -4.43874  4.24574  1.77131
H  -3.11798  5.38204  1.40738
H  -4.50737  5.24614  0.30331
H  -4.78775  4.59042 -1.83970
H  -3.67049  4.06106 -3.11960
```

H -4.93588 2.95673 -2.52749

2ccc (lowest conf)

SCF =   -2340.31015976
H(0 K)=   -2339.414533
G(298 K)=   -2339.508299
SCF(C6H6) =   -2340.31645484
BP86(D3BJ) =   -2340.67031969
Low Freq. = 12.9425cm-1, 23.7055cm-1

112
N -2.26663 2.76804 0.63586
C -1.78808 1.82398 -0.26166
N -2.61558 2.01844 -1.36212
C -3.56138 3.03113 -1.14957
C -3.33621 3.51198 0.11629
Ru -0.13357 0.57855 -0.06878
P -1.60356 -1.29101 0.11589
C -3.35462 -1.24169 -0.60626
C -2.57067 1.26362 -2.60840
C -1.71060 3.01869 1.96383
C   0.95364 2.35931 0.03464
N   1.06501 3.34110 -0.94843
C   1.81113 4.44899 -0.52059
C   2.19802 4.17692 0.76758
N   1.66558 2.91998 1.08514
C   0.48308 3.27406 -2.28274
C   1.92292 2.28857 2.37860
P   1.89454 -0.67786 -0.22822
C   2.50402 -1.17871 -1.94917
C   3.51267 0.15286 0.31586
C   2.03986 -2.28271 0.74222
C   1.64752 -2.21750 2.09893
C   1.76126 -3.34360 2.92982
C   2.24936 -4.55511 2.41368
C   2.61199 -4.63492 1.06017
C   2.51186 -3.50686 0.22862
O -0.31960 0.36227 2.20490
C -1.20807 -0.46585 2.72203
C -1.98322 -1.36571 1.91879
C -2.97088 -2.18606 2.50833
C -3.18839 -2.17695 3.89164
C -2.40437 -1.32289 4.69848
C -1.43944 -0.48713 4.13585
C -1.12252 -3.03793 -0.38887
H   1.23488 -1.28058 2.48882
C   3.03801 4.96477 1.72124
C   2.06966 5.64209 -1.38501
H   1.28137 1.40148 2.47676
H   1.67293 2.99607 3.18809
H   2.98705 2.01153 2.46084
H   1.22972 3.57539 -3.03637
H -0.39886 3.93361 -2.36724
H   0.17886 2.23078 -2.44820
H -0.84235 0.18632 4.76131
H -2.56196 -1.30601 5.78391
H -3.95711 -2.81626 4.33716
H -3.58593 -2.82910 1.86735
H   2.79173 -3.59088 -0.82484
H   2.97413 -5.58104 0.64306
H   2.33357 -5.43602 3.05953
H   1.45202 -3.27315 3.97815
H -0.05644 0.63628 -1.65977
C -4.02951 4.58838 0.88920
C -4.59508 3.39707 -2.16624
H -3.51581 0.71923 -2.76619
H -2.39351 1.94286 -3.46154
H -1.73873 0.55127 -2.52600
H -1.06322 2.16760 2.22944
H -1.13466 3.96139 1.97216
H -2.52832 3.09544 2.69935
C -4.28627 -0.34886 -0.03082
C -5.58101 -0.21884 -0.55649
C -5.97422 -0.98313 -1.66921
C -5.06268 -1.88487 -2.24049
C -3.76565 -2.01280 -1.71336
H -3.99524 0.23902 0.84548
H -6.28783 0.47497 -0.08773
H -6.98704 -0.88803 -2.07577
H -5.36109 -2.50125 -3.09598
H -3.07565 -2.73339 -2.16282
C -1.19352 -4.14212 0.48356
C -0.85442 -5.42882 0.03379
C -0.44344 -5.63458 -1.29260
C -0.35240 -4.53928 -2.16621
C -0.68180 -3.25157 -1.71335
H -1.50257 -3.99365 1.52196
H -0.91094 -6.27437 0.72805
H -0.18400 -6.64040 -1.64050
H -0.01461 -4.68159 -3.19857
H -0.58381 -2.39975 -2.39370
C   3.80803 -1.69061 -2.14080
C   4.26034 -2.04595 -3.42107
C   3.42200 -1.88421 -4.53707
C   2.13198 -1.36065 -4.36228
C   1.67985 -1.01116 -3.07801
H   4.48026 -1.80039 -1.28355
H   5.27388 -2.44230 -3.54734
H   3.77769 -2.15574 -5.53699
H   1.47491 -1.21700 -5.22757
H   0.68624 -0.57385 -2.92950
C   4.28369 -0.27622 1.41560
C   5.46300 0.40101 1.77544
C   5.89221 1.51747 1.04158
C   5.13619 1.94862 -0.06298
C   3.96085 1.27193 -0.42124
H   3.96708 -1.14777 1.99600
H   6.04880 0.04622 2.63081
H   6.81316 2.04158 1.31942
H   5.46497 2.81216 -0.65172
H   3.38423 1.61415 -1.28699
H   3.35264 5.91323 1.25826
H   2.49699 5.21317 2.65272
H   3.95113 4.41325 2.00941
H   2.65628 5.39040 -2.28822
H   1.13366 6.11971 -1.72868
H   2.64006 6.39911 -0.82434
H -4.51775 4.19999 1.80216
H -3.33443 5.38666 1.20751
H -4.81142 5.05658 0.27096
H -5.25953 2.54398 -2.39578
H -5.22469 4.21888 -1.79075
H -4.14825 3.73093 -3.12080

8tcc (lowest conf)

SCF =   -2340.24967380
H(0 K)=   -2339.356642
G(298 K)=   -2339.449683
SCF(C6H6) =   -2340.25554725
BP86(D3BJ) =   -2340.60806019
Low Freq. = 16.1417cm-1, 24.0047cm-1

112
C -0.56287 0.81797 3.00381
C -1.47887 1.50354 2.16583
C -1.89337 2.78319 2.58567
C -1.40318 3.36677 3.76774
C -0.46584 2.67668 4.55017
C -0.03789 1.39330 4.17197
P -1.84041 0.75453 0.45279
C -2.97589 2.11782 -0.22253
O -0.24047 -0.44980 2.55708
C   0.84977 -1.19216 2.99346
C   1.85337 -1.50645 2.04669
C   2.86369 -2.39688 2.47745
C   2.88498 -2.93041 3.77524
C   1.86946 -2.60143 4.68713
C   0.83872 -1.73973 4.29009
P   1.81503 -0.77528 0.26180
C   2.80925 -2.16689 -0.58056
Ru -0.08042 0.00419 -0.99985
C   0.71570 1.90983 -1.39796

```
N   0.91801  2.43115 -2.67896
C   1.47585  3.71957 -2.66101
C   1.63238  4.05130 -1.33879
N   1.16833  2.95667 -0.60071
C   0.61614  1.72473 -3.91843
C   1.21450  2.94153  0.85264
C  -0.94191 -1.87357 -1.38838
N  -1.28550 -2.34376 -2.65657
C  -1.88042 -3.61485 -2.62483
C  -1.91496 -3.99127 -1.30556
N  -1.34634 -2.93558 -0.58247
C  -1.06252 -1.61460 -3.89936
C  -1.16641 -2.99527  0.85882
C   3.25091  0.44776  0.47110
C  -3.22205 -0.45373  0.91714
C   1.79990  4.50704 -3.89160
C   2.18156  5.28519 -0.69668
C  -2.44395 -5.22756 -0.65107
C  -2.35993 -4.34104 -3.84224
H  -2.11019 -3.28012  1.35067
H  -0.87493 -1.99588  1.19550
H  -0.37860 -3.72219  1.12697
H  -0.06771 -1.15060 -3.85835
H  -1.81123 -0.81382 -4.02699
H  -1.12513 -2.31487 -4.74574
H  -0.38319  1.27431 -3.83969
H   1.34099  0.91113 -4.09123
H   0.65146  2.43574 -4.75709
H   0.51285  3.67642  1.28122
H   2.23649  3.15598  1.20369
H   0.92082  1.93864  1.17907
H   0.00927 -1.49919  4.96206
H   1.86686 -3.02614  5.69647
H   3.69201 -3.60971  4.06942
H   3.65407 -2.67881  1.77536
H  -2.59953  3.34276  1.96537
H  -1.74829  4.36171  4.06762
H  -0.06369  3.12924  5.46290
H   0.69219  0.84595  4.77414
H  -1.12189  0.43667 -2.16927
H   0.88635 -0.40671 -2.24775
C  -3.61143 -0.73785  2.24175
C  -4.63226 -1.66787  2.50808
C  -5.28724 -2.32063  1.45187
C  -4.91699 -2.03344  0.12567
C  -3.89144 -1.11288 -0.13742
H  -3.12212 -0.22218  3.07418
H  -4.92020 -1.87450  3.54515
H  -6.08706 -3.04018  1.65858
H  -5.42837 -2.52846 -0.70742
H  -3.59901 -0.90072 -1.17149
C  -4.27439  2.33492  0.29122
C  -5.09183  3.35398 -0.22381
C  -4.62541  4.17563 -1.26335
C  -3.33818  3.96825 -1.78272
C  -2.52231  2.94594 -1.26814
H  -4.65308  1.69830  1.09748
H  -6.09696  3.50252  0.18654
H  -5.26444  4.96794 -1.66858
H  -2.96704  4.59891 -2.59851
H  -1.52846  2.76608 -1.68622
C   3.84305  0.78532  1.70552
C   4.87301  1.74109  1.77483
C   5.33659  2.36826  0.60809
C   4.76186  2.03196 -0.63100
C   3.72661  1.08771 -0.69623
H   3.50280  0.29988  2.62491
H   5.31634  1.98866  2.74599
H   6.14393  3.10692  0.66078
H   5.11890  2.50882 -1.55048
H   3.27202  0.83822 -1.66076
C   4.22025 -2.18346 -0.64612
C   4.90351 -3.23609 -1.28045
C   4.19049 -4.29926 -1.85408
C   2.78762 -4.29758 -1.79135
C   2.10678 -3.24060 -1.16703
H   4.79694 -1.36614 -0.20360
H   5.99849 -3.22069 -1.32259
H   4.72231 -5.11940 -2.34870
H   2.21605 -5.11860 -2.23872
H   1.01484 -3.23492 -1.15195
H  -3.12135 -3.76765 -4.40226
H  -1.54057 -4.57453 -4.54770
H  -2.81947 -5.29828 -3.54953
H  -1.66783 -5.75787 -0.06940
H  -3.27430 -4.99911  0.04220
H  -2.82604 -5.92940 -1.40912
H   2.52675  3.99051 -4.54527
H   0.90513  4.72553 -4.50422
H   2.24384  5.47456 -3.60888
H   2.46269  6.02423 -1.46345
H   1.44779  5.76673 -0.02469
H   3.08351  5.06768 -0.09569
```

8ctc

```
SCF  =           -2340.23079832
H(0 K)=          -2339.339647
G(298 K)=        -2339.434508
SCF(C6H6) =      -2340.23701167
BP86(D3BJ) =     -2340.58056840
Low Freq. = 13.0108cm-1, 19.1969cm-1
```

```
112
N  -0.67225 -0.33438  3.23896
C  -0.72768 -0.96775  1.99674
N  -1.47480 -2.10837  2.30837
C  -1.85459 -2.16853  3.65740
C  -1.34076 -1.04211  4.24852
Ru -0.02857 -0.28789  0.07654
C   0.41288 -2.18780 -0.79745
N   0.26668 -2.62203 -2.11777
C   0.82422 -3.89097 -2.34299
C   1.34906 -4.30055 -1.14359
N   1.09085 -3.26768 -0.23072
C  -0.32920 -1.84214 -3.19483
C   1.55591 -3.30578  1.14656
C  -1.91034 -3.10723  1.34448
C  -0.05713  0.96133  3.50101
P   2.07331  0.58529  0.16270
C   3.47450 -0.28236 -0.76958
C   2.31927  2.31667 -0.61732
C   1.30919  3.18573 -1.10705
C   1.61328  4.50597 -1.50263
C   2.93403  4.96793 -1.50286
C   3.96230  4.10548 -1.09316
C   3.64449  2.81506 -0.65065
O  -0.02875  2.80425 -1.11813
C  -0.68514  2.50984 -2.30328
C  -1.80364  1.63584 -2.19395
C  -2.56934  1.41333 -3.35627
C  -2.22359  1.98168 -4.59450
C  -1.07963  2.78389 -4.68807
C  -0.31000  3.05235 -3.54442
P  -2.04370  0.61072 -0.62417
C  -2.87221  1.83558  0.54006
C   2.89244  0.92946  1.83586
C  -3.61438 -0.35269 -1.11378
H  -0.16258  1.20456  0.74002
H   0.38754  0.22497 -1.42556
H   0.57765  3.68517 -3.61623
H  -0.78048  3.21732 -5.64860
H  -2.83882  1.78214 -5.47788
H  -3.44145  0.75611 -3.29625
H   4.45344  2.16348 -0.30460
H   5.00511  4.43903 -1.09928
H   3.15483  5.99246 -1.82046
H   0.78864  5.15873 -1.80440
C   2.07661 -5.55265 -0.77087
C   0.79534 -4.56748 -3.67637
C  -1.40590 -0.57092  5.66661
C  -2.65857 -3.29326  4.22808
H   0.39676 -1.70197 -4.01506
H  -1.22735 -2.33960 -3.60069
```

```
H  -0.59764 -0.85915 -2.78586
H   2.65617 -3.39738  1.18064
H   1.24569 -2.35925  1.61350
H   1.11363 -4.15760  1.69370
H   0.84593  0.85704  4.12534
H   0.21083  1.39516  2.52959
H  -0.78046  1.62444  4.00459
H  -1.47060 -2.83326  0.37706
H  -1.56883 -4.11526  1.64033
H  -3.01049 -3.11538  1.25606
C   3.20766 -0.66843 -2.10304
C   4.17868 -1.32695 -2.87081
C   5.43598 -1.62767 -2.31898
C   5.71358 -1.25058 -0.99640
C   4.74509 -0.57792 -0.23022
H   2.22421 -0.44499 -2.52963
H   3.95314 -1.60727 -3.90599
H   6.19315 -2.14636 -2.91694
H   6.69342 -1.46872 -0.55632
H   4.98966 -0.27677  0.79310
C   3.00236  2.21864  2.40143
C   3.46436  2.39451  3.71740
C   3.84036  1.28731  4.49349
C   3.73808 -0.00357  3.94401
C   3.25441 -0.17726  2.63892
H   2.71991  3.09720  1.81430
H   3.53353  3.40585  4.13370
H   4.20781  1.42597  5.51593
H   4.02842 -0.87857  4.53657
H   3.14932 -1.18921  2.23525
C  -3.06324  3.19403  0.22074
C  -3.67116  4.06799  1.13954
C  -4.10856  3.59319  2.38515
C  -3.93444  2.23592  2.70772
C  -3.31765  1.36666  1.79561
H  -2.73940  3.57361 -0.75328
H  -3.80740  5.12252  0.87452
H  -4.58571  4.27350  3.09892
H  -4.28184  1.85191  3.67361
H  -3.17789  0.31308  2.05921
C  -3.47207 -1.70132 -1.49564
C  -4.58840 -2.47528 -1.85499
C  -5.87338 -1.90966 -1.82490
C  -6.03154 -0.56751 -1.43985
C  -4.91214  0.20523 -1.08970
H  -2.46883 -2.14094 -1.49360
H  -4.45570 -3.52251 -2.15027
H  -6.74781 -2.51145 -2.09571
H  -7.03111 -0.11907 -1.41203
H  -5.04817  1.25061 -0.79378
H   1.27162 -5.55844 -3.61147
H  -0.23524 -4.71818 -4.04788
H   1.33660 -3.99102 -4.44936
H   3.10727 -5.34766 -0.42681
H   1.56780 -6.10872  0.03847
H   2.14786 -6.22597 -1.63958
H  -1.92802  0.39894  5.76247
H  -1.94914 -1.30133  6.28687
H  -0.40035 -0.44114  6.10706
H  -3.64528 -3.39280  3.73901
H  -2.14751 -4.26903  4.12636
H  -2.83870 -3.12622  5.30173
```

8ctc (from IRC)

```
SCF  =          -2340.22877312
H(0 K)=         -2339.338009
G(298 K)=       -2339.432098
SCF(C6H6) =     -2340.23482532
BP86(D3BJ) =    -2340.57999678
Low Freq. = 16.5476cm-1, 22.1580cm-1
```

```
112
N  -0.85987 -3.11619 -1.23249
C  -0.15472 -1.93431 -1.44497
N   0.30166 -2.11586 -2.74744
C  -0.10759 -3.33483 -3.30996
C  -0.84634 -3.97160 -2.34411
Ru  0.03640 -0.29456 -0.09033
C   0.78225 -1.59310  1.43805
N   0.58599 -1.58444  2.81779
C   1.26737 -2.62099  3.47314
C   1.94024 -3.31581  2.49991
N   1.64391 -2.67935  1.28564
C  -0.19639 -0.59482  3.54828
C   2.29527 -3.06612  0.03955
C   1.14218 -1.16397 -3.45468
C  -1.56487 -3.44598 -0.00316
P  -2.12430  0.40422  0.16986
C  -3.03763  0.18430  1.81309
C  -2.45942  2.28417 -0.04392
C  -1.47867  3.30509 -0.14432
C  -1.83721  4.65150 -0.36570
C  -3.18294  5.02528 -0.44473
C  -4.17677  4.04211 -0.32061
C  -3.80811  2.70344 -0.13451
O  -0.13034  2.98570 -0.16650
C   0.74668  3.27864  0.86004
C   1.92449  2.48361  0.86620
C   2.92970  2.81371  1.79101
C   2.74846  3.84493  2.73186
C   1.54511  4.56259  2.75365
C   0.53766  4.28765  1.81235
P   1.94346  1.00864 -0.32408
C   2.35685  2.03504 -1.86811
C  -3.42618 -0.21944 -1.06096
C   3.61200  0.22147  0.05288
H  -0.38531  0.58698 -1.41985
H  -0.03566  0.79486  1.12835
H  -0.39399  4.85991  1.80876
H   1.38591  5.35465  3.49336
H   3.54405  4.07511  3.44815
H   3.86332  2.24303  1.79403
H  -4.59720  1.94866 -0.06606
H  -5.23627  4.31131 -0.38311
H  -3.45035  6.07350 -0.61513
H  -1.03672  5.38684 -0.49126
C   2.84528 -4.50248  2.59807
C   1.19530 -2.83519  4.95176
C  -1.55187 -5.29008 -2.36373
C   0.24826 -3.75445 -4.70076
H  -0.43224  0.21695  2.84908
H  -1.13410 -1.02501  3.93831
H   0.39545 -0.19330  4.38833
H   3.37686 -3.19518  0.20838
H   1.87147 -4.00252 -0.36553
H   2.13204 -2.25496 -0.68047
H  -1.45046 -2.58314  0.66917
H  -2.63442 -3.62817 -0.20783
H  -1.13253 -4.34359  0.47351
H   1.55667 -0.47423 -2.70792
H   1.95890 -1.68823 -3.97833
H   0.56482 -0.57872 -4.19227
C  -3.52418 -1.09037  2.18793
C  -4.06616 -1.31625  3.46226
C  -4.11039 -0.27555  4.40751
C  -3.61895  0.99153  4.05573
C  -3.09593  1.22005  2.77177
H  -3.48494 -1.91586  1.47045
H  -4.45354 -2.30892  3.71868
H  -4.52659 -0.45147  5.40517
H  -3.64810  1.81262  4.78089
H  -2.72598  2.21724  2.51338
C  -4.76192 -0.54056 -0.72964
C  -5.65514 -1.01219 -1.70728
C  -5.23632 -1.15937 -3.03843
C  -3.91314 -0.83509 -3.38275
C  -3.01765 -0.37874 -2.40407
H  -5.11436 -0.42412  0.30000
H  -6.68528 -1.25675 -1.42404
H  -5.93411 -1.52217 -3.80094
H  -3.57391 -0.94300 -4.41919
H  -1.97965 -0.14851 -2.66549
C   1.40107  2.19968 -2.88770
```

```
C   1.66161  3.02595 -3.99421
C   2.88619  3.70340 -4.09592
C   3.84194  3.56264 -3.07530
C   3.57562  2.74359 -1.96751
H   0.43866  1.68457 -2.78544
H   0.90083  3.14469 -4.77400
H   3.09234  4.34634 -4.95865
H   4.79507  4.09978 -3.13603
H   4.32081  2.66061 -1.16955
C   3.86512 -0.26001  1.36117
C   5.00711 -1.02470  1.64441
C   5.91496 -1.35350  0.62211
C   5.66548 -0.90425 -0.68556
C   4.53040 -0.12674 -0.96576
H   3.15134 -0.04462  2.16288
H   5.18520 -1.36791  2.66986
H   6.80650 -1.95026  0.84279
H   6.36329 -1.15132 -1.49383
H   4.36141  0.22029 -1.99025
H   1.76016 -3.73766  5.23418
H   1.62138 -1.98806  5.52099
H   0.15542 -2.96903  5.30212
H   3.88158 -4.26090  2.29714
H   2.88023 -4.87003  3.63588
H   2.50823 -5.33971  1.95959
H  -2.64275 -5.18013 -2.22068
H  -1.39510 -5.79266 -3.33114
H  -1.18752 -5.97218 -1.57331
H  -0.09693 -3.02535 -5.45660
H   1.33920 -3.87382 -4.83972
H  -0.21991 -4.72284 -4.93792
```

TS(8-2)ctc

```
SCF  =          -2340.19156193
H(0 K)=         -2339.303224
G(298 K)=       -2339.397221
SCF(C6H6) =     -2340.19862300
BP86(D3BJ) =    -2340.54707675
Low Freq. = -413.8333cm-1, 14.6379cm-1
```

```
112
N  -0.68342 -2.91833 -1.44477
C  -0.07780 -1.66775 -1.56153
N   0.39912 -1.71053 -2.87072
C   0.11263 -2.92278 -3.51757
C  -0.58017 -3.68728 -2.61375
Ru  0.00752 -0.14222 -0.16642
C   0.70115 -1.41591  1.42672
N   0.46896 -1.36272  2.79687
C   1.14655 -2.36394  3.50742
C   1.84787 -3.08854  2.57686
N   1.57302 -2.50092  1.33326
C  -0.33351 -0.35200  3.47500
C   2.22309 -2.96187  0.11223
C   1.15086 -0.64140 -3.50944
C  -1.36744 -3.40071 -0.25652
P  -2.21838  0.32871  0.05462
C  -3.18452 -0.04792  1.64206
C  -2.53361  2.18869 -0.06513
C  -1.54702  3.22153 -0.00074
C  -1.94126  4.57155 -0.16862
C  -3.27249  4.92814 -0.39309
C  -4.25640  3.92713 -0.44009
C  -3.87643  2.59063 -0.27490
O  -0.18325  3.06808  0.11826
C   0.42326  2.43951  1.30357
C   1.85170  2.17325  1.14095
C   2.77340  2.59072  2.09789
C   2.35772  3.19775  3.30593
C   0.98059  3.37756  3.52215
C   0.02896  3.03359  2.55334
P   2.11711  1.02065 -0.28497
C   2.63110  2.20265 -1.66635
C  -3.41391 -0.29697 -1.26732
C   3.72433  0.11914  0.06305
H  -0.37924  0.83947 -1.43525
H  -0.12620  1.16562  1.05688
H  -1.02782  3.27427  2.71066
H   0.63378  3.84542  4.45211
H   3.09135  3.48704  4.06388
H   3.83667  2.37480  1.94015
H  -4.64842  1.81607 -0.33054
H  -5.30759  4.18024 -0.61237
H  -3.54082  5.98238 -0.52205
H  -1.15039  5.32554 -0.11546
C   2.76207 -4.26086  2.73909
C   1.04850 -2.51299  4.99233
C  -1.17426 -5.05371 -2.74048
C   0.52369 -3.21069 -4.92636
H  -0.70674  0.34243  2.71623
H  -1.18808 -0.81166  3.99910
H   0.28489  0.20985  4.19439
H   3.30711 -3.06592  0.28045
H   1.81018 -3.93061 -0.22225
H   2.04916 -2.20878 -0.66524
H  -1.23424 -2.63556  0.52067
H  -2.44279 -3.55073 -0.45966
H  -0.93281 -4.35645  0.08596
H   0.99625  0.26882 -2.91922
H   2.23083 -0.87285 -3.54619
H   0.78771 -0.48152 -4.53792
C  -3.47266 -1.38839  1.98894
C  -4.07764 -1.70958  3.21358
C  -4.39181 -0.69470  4.13434
C  -4.10764  0.64011  3.80773
C  -3.51724  0.96009  2.57312
H  -3.22878 -2.19223  1.28912
H  -4.30322 -2.75574  3.44939
H  -4.85778 -0.94334  5.09379
H  -4.35156  1.44240  4.51291
H  -3.31699  2.00751  2.33006
C  -4.70065 -0.81109 -0.99865
C  -5.52269 -1.26937 -2.04338
C  -5.07797 -1.20991 -3.37274
C  -3.80226 -0.69020 -3.65157
C  -2.97589 -0.24516 -2.60938
H  -5.07109 -0.85042  0.03031
H  -6.51838 -1.66499 -1.81323
H  -5.72111 -1.56229 -4.18642
H  -3.44753 -0.63304 -4.68673
H  -1.97608  0.14779 -2.82040
C   1.64071  2.71446 -2.52901
C   1.96308  3.67027 -3.50534
C   3.28141  4.13718 -3.62966
C   4.27173  3.65089 -2.76102
C   3.94960  2.69446 -1.78472
H   0.60642  2.37403 -2.40191
H   1.17783  4.05636 -4.16478
H   3.53403  4.88307 -4.39110
H   5.30053  4.02014 -2.83747
H   4.73167  2.33505 -1.10877
C   4.04083 -0.30992  1.37238
C   5.15263 -1.13421  1.61148
C   5.96401 -1.56297  0.54700
C   5.65010 -1.15978 -0.76220
C   4.54173 -0.33235 -1.00024
H   3.41119  0.00513  2.20961
H   5.38749 -1.43727  2.63808
H   6.83441 -2.20050  0.73566
H   6.27403 -1.48481 -1.60233
H   4.31611 -0.02134 -2.02607
H   1.64638 -3.37418  5.32989
H   1.42183 -1.61990  5.52635
H   0.00771 -2.67821  5.32636
H   3.79694 -4.02310  2.43065
H   2.79444 -4.57505  3.79422
H   2.43627 -5.13292  2.14269
H  -2.27185 -5.04257 -2.60791
H  -0.96714 -5.46819 -3.73953
H  -0.76373 -5.76182 -1.99688
H   0.05061 -2.52187 -5.65087
H   1.61708 -3.12951 -5.06728
H   0.22925 -4.23422 -5.20663
```

2ctc (from IRC)

SCF = -2340.29453567
H(0 K)= -2339.399980
G(298 K)= -2339.496671
SCF(C6H6) = -2340.30186232
BP86(D3BJ) = -2340.64303532
Low Freq. = 9.0046cm-1, 17.7904cm-1

112
N -1.03609 -0.04921 -2.98173
C -0.44421 0.60391 -1.88811
N -0.32598 1.91061 -2.38723
C -0.81017 2.04193 -3.69780
C -1.25501 0.80317 -4.07666
Ru -0.07054 -0.07294 -0.00046
C 0.27862 -2.16518 -0.48071
N -0.15943 -3.29887 0.19670
C 0.33372 -4.49312 -0.35006
C 1.12736 -4.13136 -1.41012
N 1.07886 -2.73137 -1.47216
C -1.05536 -3.28871 1.34900
C 1.81557 -1.97578 -2.47465
C 0.15496 3.07377 -1.64944
C -1.43366 -1.45005 -3.02488
P -2.29818 0.18594 0.64478
C -3.70785 -1.02126 0.29063
C -2.13830 0.19178 2.47924
C -0.83991 -0.18723 2.95465
C -0.64367 -0.20897 4.37300
C -1.67265 0.14651 5.24507
C -2.94238 0.53712 4.76141
C -3.16328 0.55658 3.37921
O 0.14399 -0.52467 2.14075
C 2.89850 -1.90043 1.72077
C 3.17452 -0.52509 1.58999
C 4.18520 0.04412 2.39385
C 4.90945 -0.74812 3.30011
C 4.63363 -2.11982 3.41580
C 3.62586 -2.69236 2.62342
P 2.23049 0.48931 0.31269
C 2.46949 2.20363 1.05789
C -3.17163 1.83250 0.31599
C 3.51319 0.54057 -1.07645
H -0.23776 1.46996 0.48962
H 2.09118 -2.33898 1.13013
H 3.39332 -3.75888 2.71737
H 5.19581 -2.73696 4.12516
H 5.68901 -0.28868 3.91781
H 4.40861 1.11254 2.31469
H -4.13814 0.86885 2.98512
H -3.73745 0.82947 5.45457
H -1.48754 0.12921 6.32601
H 0.34269 -0.50419 4.74663
C 1.93114 -4.96294 -2.35856
C -0.00673 -5.84314 0.19654
C -1.86545 0.34129 -5.36181
C -0.80532 3.34208 -4.43706
H -1.04950 -2.27690 1.76599
H -2.07795 -3.58246 1.05890
H -0.68214 -3.98538 2.11719
H 2.90104 -2.00443 -2.28190
H 1.62384 -2.38549 -3.48190
H 1.47525 -0.93469 -2.42526
H -1.45130 -1.82977 -1.99622
H -2.43676 -1.54328 -3.47036
H -0.72939 -2.05189 -3.62652
H 0.50980 2.73039 -0.67009
H 0.97467 3.57144 -2.19625
H -0.66505 3.79513 -1.49385
C -4.17601 -1.17791 -1.03404
C -5.16818 -2.12072 -1.34638
C -5.70573 -2.93805 -0.33783
C -5.24912 -2.79460 0.98208
C -4.26224 -1.84384 1.29450
H -3.76945 -0.54019 -1.82634
H -5.52351 -2.21631 -2.37877
H -6.47775 -3.67654 -0.57903
H -5.66782 -3.42013 1.77848
H -3.92014 -1.73236 2.32838
C -4.48236 1.94613 -0.19065
C -5.08229 3.20732 -0.36065
C -4.39005 4.37521 -0.01175
C -3.09106 4.27302 0.51586
C -2.48921 3.01634 0.67429
H -5.05380 1.04964 -0.44577
H -6.10112 3.26908 -0.75928
H -4.86025 5.35643 -0.13870
H -2.54374 5.17542 0.81074
H -1.47719 2.93977 1.08628
C 1.69174 2.52099 2.19510
C 1.82310 3.77082 2.81714
C 2.72043 4.72691 2.30756
C 3.49610 4.41780 1.18092
C 3.37918 3.15820 0.56325
H 0.99058 1.77610 2.58524
H 1.21930 3.99867 3.70230
H 2.81535 5.70580 2.79004
H 4.20166 5.15348 0.77884
H 4.00060 2.92455 -0.30663
C 4.78792 -0.05716 -0.97358
C 5.68703 -0.02514 -2.05404
C 5.33424 0.61163 -3.25381
C 4.06942 1.21271 -3.36849
C 3.16747 1.17001 -2.29404
H 5.08357 -0.55012 -0.04320
H 6.67031 -0.49758 -1.95118
H 6.03709 0.63839 -4.09339
H 3.77949 1.71020 -4.30076
H 2.17358 1.61130 -2.40772
H 0.46589 -6.63025 -0.41180
H 0.34277 -5.97467 1.23728
H -1.09608 -6.02952 0.19401
H 3.00593 -4.70628 -2.32855
H 1.83886 -6.02981 -2.10150
H 1.59869 -4.84644 -3.40707
H -2.90552 -0.01259 -5.23284
H -1.89076 1.16940 -6.08722
H -1.29596 -0.48624 -5.82358
H -1.41197 4.11195 -3.92608
H 0.21337 3.75599 -4.55326
H -1.22452 3.20759 -5.44642

2ttt

SCF = -2340.31236872
H(0 K)= -2339.416549
G(298 K)= -2339.510844
SCF(C6H6) = -2340.31828521
BP86(D3BJ) = -2340.66996203
Low Freq. = 12.4684cm-1, 17.6552cm-1

112
Ru -0.04095 0.02620 -0.03585
H 0.09552 0.04134 -1.62385
P -2.38431 -0.19717 0.08934
P 2.30426 0.18646 -0.02659
O -0.38820 0.14234 2.23198
N 0.06283 3.12842 0.69010
N -0.71440 2.84025 -1.30402
N 0.27146 -2.87140 -1.30815
N 0.14282 -3.06132 0.83764
C -0.25069 2.11711 -0.20787
C -0.18266 4.40831 0.17449
C -0.68370 4.22430 -1.08963
C 0.67836 2.92418 1.99877
H 0.49523 1.88216 2.29950
H 0.21376 3.59916 2.73596
H 1.76151 3.13471 1.95218
C 0.09384 5.66258 0.94025
H -0.15357 6.54354 0.32732
H 1.15812 5.74456 1.22608
H -0.50146 5.72806 1.86980

```
C  -1.16141  5.21177 -2.10568
H  -2.22886  5.06225 -2.35155
H  -0.59496  5.14802 -3.05296
H  -1.04777  6.23776 -1.72195
C  -1.17483  2.27994 -2.56823
H  -0.54180  2.64017 -3.39967
H  -2.22211  2.56436 -2.76163
H  -1.08899  1.18802 -2.49272
C   0.14538 -2.09245 -0.15830
C   0.34109 -4.24220 -1.02857
C   0.25541 -4.36432  0.33466
C   0.39982 -2.38775 -2.67715
H   1.41126 -2.59573 -3.06662
H   0.23344 -1.30174 -2.65778
H  -0.34777 -2.87715 -3.32471
C   0.47540 -5.28281 -2.09368
H  -0.39235 -5.29051 -2.77885
H   0.54505 -6.28389 -1.63980
H   1.38061 -5.12995 -2.70896
C   0.24791 -5.57667  1.20991
H   1.07391 -5.56914  1.94459
H   0.35516 -6.48716  0.59969
H  -0.69397 -5.66887  1.78116
C   0.04112 -2.80347  2.27033
H  -0.88096 -3.25414  2.67637
H   0.01314 -1.71466  2.42165
H   0.90955 -3.23632  2.79621
C  -2.77064  0.03091  1.87437
C  -1.61675  0.18509  2.71076
C  -1.84787  0.38828  4.10990
H  -0.97435  0.50348  4.76151
C  -3.14174  0.44739  4.62717
H  -3.28234  0.60783  5.70306
C  -4.27077  0.31398  3.78776
H  -5.28300  0.37574  4.19960
C  -4.07292  0.11285  2.41664
H  -4.93882  0.02969  1.74865
C  -3.15341 -1.83632 -0.40455
C  -3.86473 -2.64728  0.50237
H  -3.99593 -2.31277  1.53617
C  -4.40491 -3.87753  0.08934
H  -4.95590 -4.49432  0.80810
C  -4.24860 -4.31282 -1.23573
H  -4.67714 -5.26820 -1.55727
C  -3.53402 -3.51639 -2.14638
H  -3.40267 -3.84856 -3.18252
C  -2.98109 -2.29522 -1.72951
H  -2.40251 -1.69043 -2.43649
C  -3.59897  1.01677 -0.69668
C  -4.42746  0.70433 -1.79385
H  -4.43464 -0.31032 -2.20322
C  -5.26745  1.67849 -2.36143
H  -5.90857  1.41153 -3.20897
C  -5.29448  2.98110 -1.84000
H  -5.95300  3.73851 -2.27889
C  -4.48239  3.30022 -0.73720
H  -4.50709  4.30824 -0.30858
C  -3.64394  2.32790 -0.17156
H  -3.02343  2.58239  0.69356
C   3.22930 -0.99830 -1.17901
C   3.33754 -2.35316 -0.79279
H   2.93016 -2.67731  0.17014
C   3.96694 -3.29184 -1.62435
H   4.04386 -4.33523 -1.29927
C   4.50097 -2.89550 -2.86314
H   4.99607 -3.62670 -3.51127
C   4.40656 -1.55114 -3.25391
H   4.83114 -1.22449 -4.20973
C   3.77826 -0.61086 -2.41801
H   3.73227  0.43559 -2.73332
C   3.01718  1.83881 -0.58902
C   2.52227  2.39533 -1.79061
H   1.74622  1.85666 -2.34409
C   3.00053  3.62666 -2.26437
H   2.61309  4.03458 -3.20458
C   3.96684  4.33806 -1.53320
H   4.33626  5.30221 -1.89885
C   4.45062  3.80548 -0.32890
H   5.20103  4.35294  0.25229
C   3.98422  2.56351  0.13762
H   4.38038  2.15792  1.07322
C   3.30987 -0.14200  1.53488
C   2.65735 -0.16093  2.78504
H   1.57210 -0.00894  2.83080
C   3.39158 -0.39599  3.96215
H   2.87249 -0.41269  4.92710
C   4.77634 -0.61422  3.90659
H   5.34393 -0.79871  4.82538
C   5.43267 -0.60161  2.66357
H   6.51303 -0.77611  2.60927
C   4.70493 -0.37053  1.48642
H   5.22365 -0.37204  0.52202
```

2. Ligand Dissociation from Ru(IMe4)2(DPEphos)H2

(i) IMe4 Dissociation

8ccc-IMe4 trans P

```
SCF  =          -1956.77271846
H(0 K)=         -1956.060524
G(298 K)=       -1956.143413
SCF(C6H6) =     -1956.77953493
BP86(D3BJ) =    -1957.05050685
Low Freq. = 17.5117cm-1, 20.5285cm-1
```

```
91
N   0.18589  2.82796  0.69691
C  -0.19238  1.98485 -0.34427
N  -0.72158  2.88863 -1.25955
C  -0.66744  4.21504 -0.80863
C  -0.08668  4.17866  0.43504
Ru  0.03638 -0.10930 -0.56782
O   0.15516 -0.87874  1.66950
C  -1.05173 -0.89541  2.39221
C  -2.25016 -0.78635  1.65168
C  -3.45577 -0.72344  2.38205
C  -3.47069 -0.73979  3.78492
C  -2.26046 -0.80456  4.49255
C  -1.04384 -0.87572  3.79714
P  -2.15897 -0.65351 -0.23874
C  -3.56286  0.54178 -0.59836
C  -1.30913  2.52713 -2.54469
C   0.79213  2.37853  1.94172
P   2.28416 -0.38954 -0.33324
C   3.35174 -1.21481 -1.63891
C   2.35141 -1.62686  1.09029
C   1.20270 -1.79076  1.90491
C   1.10978 -2.83655  2.83484
C   2.19891 -3.70806  3.00320
C   3.36627 -3.54219  2.24627
C   3.42699 -2.51402  1.29177
C   3.45304  0.97365  0.23405
C  -2.93439 -2.29128 -0.76543
H   0.19280 -2.99092  3.40672
H   2.11712 -4.52685  3.72547
H   4.21355 -4.22309  2.37456
H   4.31242 -2.41826  0.65476
H   0.03715  0.12314 -2.12669
H   0.25419 -1.67660 -1.06216
H  -4.39957 -0.62740  1.83669
H  -4.42269 -0.68062  4.32217
H  -2.25481 -0.78627  5.58716
H  -0.09306 -0.90109  4.33619
H  -2.38915  2.75273 -2.55620
H  -0.81222  3.07612 -3.36342
H  -1.15092  1.44756 -2.67857
C  -1.18056  5.36654 -1.61303
C   0.24850  5.27647  1.39357
H   1.80143  2.80552  2.06646
H   0.86828  1.28520  1.89884
H   0.16734  2.66597  2.80597
H  -0.05155  6.25132  0.97779
```

```
H  -0.26725  5.15868  2.36489
H   1.33266  5.32514  1.60522
H  -0.65127  5.47217 -2.57820
H  -2.25684  5.26258 -1.84259
H  -1.04791  6.30919 -1.05890
C  -3.70965  1.71751  0.17085
C  -4.63077  2.70872 -0.20164
C  -5.41252  2.55078 -1.35926
C  -5.26576  1.39192 -2.13945
C  -4.34818  0.39734 -1.76373
H  -3.09528  1.85817  1.06616
H  -4.73724  3.60816  0.41501
H  -6.13179  3.32417 -1.64937
H  -5.86945  1.25762 -3.04385
H  -4.24404 -0.50116 -2.38090
C  -2.11291 -3.25429 -1.38125
C  -2.63265 -4.50658 -1.74939
C  -3.98039 -4.81162 -1.50397
C  -4.80942 -3.85616 -0.89190
C  -4.29127 -2.60401 -0.52742
H  -1.05999 -2.99830 -1.55441
H  -1.98043 -5.24410 -2.23006
H  -4.38648 -5.78806 -1.78992
H  -5.86366 -4.08453 -0.69984
H  -4.95532 -1.86335 -0.07017
C   4.75843 -1.09849 -1.65902
C   5.51011 -1.73448 -2.66109
C   4.86524 -2.48652 -3.65613
C   3.46470 -2.59566 -3.64859
C   2.71064 -1.96106 -2.64918
H   5.26915 -0.49974 -0.89739
H   6.60138 -1.63664 -2.66544
H   5.45198 -2.97818 -4.43977
H   2.95463 -3.17011 -4.42975
H   1.61699 -2.02201 -2.63607
C   4.23570  0.91676  1.40599
C   5.02689  2.01385  1.79152
C   5.04999  3.17854  1.00799
C   4.27492  3.24370 -0.16410
C   3.47778  2.15352 -0.54287
H   4.22548  0.01229  2.02339
H   5.62867  1.95456  2.70543
H   5.66762  4.03197  1.30807
H   4.28698  4.14922 -0.78085
H   2.85354  2.21490 -1.44169
```

8cct-IMe4 trans IMe4

```
SCF  =          -1956.77034029
H(0 K)=         -1956.059266
G(298 K)=       -1956.142055
SCF(C6H6) =     -1956.77715723
BP86(D3BJ) =    -1957.04222900
Low Freq. = 14.0281cm-1, 15.5829cm-1

91
N   0.50070  2.97499  0.96295
C  -0.00002  2.10039 -0.00012
N  -0.50080  2.97500 -0.96316
C  -0.32123  4.31954 -0.60645
C   0.32094  4.31955  0.60636
Ru -0.00001  0.11579 -0.00009
P   2.25378 -0.31020 -0.03247
C   3.39565  0.61637 -1.18460
C  -1.14872  2.55554 -2.19715
C  -0.77822  5.45909 -1.46071
C   0.77779  5.45913  1.46064
C   1.14857  2.55547  2.19696
C   2.26166 -2.06201 -0.72994
C   1.07134 -2.81497 -0.62703
C   0.94868 -4.08653 -1.20704
C   2.05840 -4.64561 -1.86115
C   3.26901 -3.94059 -1.93790
C   3.36047 -2.65637 -1.37764
O  -0.00009 -2.16237 -0.00009
C  -1.07122 -2.81480  0.62756
C  -2.26153 -2.06183  0.73056
C  -3.36011 -2.65593  1.37888
C  -3.26843 -3.93993  1.93962
C  -2.05785 -4.64497  1.86271
C  -0.94835 -4.08613  1.20799
P  -2.25382 -0.31030  0.03240
C  -3.39585  0.61660  1.18410
C  -3.32694 -0.59042 -1.49798
C   3.32707 -0.58963  1.49792
H  -0.95704  3.29748 -2.98792
H  -2.24016  2.44356 -2.06517
H  -0.72395  1.57740 -2.47113
H   2.23989  2.44253  2.06477
H   0.72318  1.57773  2.47143
H   0.95770  3.29790  2.98745
H  -0.18717  0.08634  1.68660
H  -4.29255 -2.08947  1.46874
H  -4.13226 -4.37814  2.44935
H  -1.96316 -5.63409  2.32235
H   0.00238 -4.62265  1.16750
H  -0.00207 -4.62302 -1.16666
H   1.96389 -5.63492 -2.32043
H   4.13302 -4.37899 -2.44716
H   4.29294 -2.08993 -1.46739
H   0.18710  0.08626 -1.68677
H   1.86480  5.42317  1.65870
H   0.56575  6.41639  0.95906
H   0.26705  5.48274  2.44165
H  -0.26754  5.48272 -2.44174
H  -1.86524  5.42300 -1.65872
H  -0.56627  6.41638 -0.95915
C  -4.72734 -0.76729 -1.46073
C  -5.43851 -1.05822 -2.63581
C  -4.75997 -1.18404 -3.85989
C  -3.36615 -1.01992 -3.90289
C  -2.65431 -0.72381 -2.72879
H  -5.26933 -0.67173 -0.51410
H  -6.52560 -1.18904 -2.59429
H  -5.31719 -1.41123 -4.77528
H  -2.82977 -1.12115 -4.85306
H  -1.56252 -0.59506 -2.73863
C  -4.28245  1.60035  0.69734
C  -5.02362  2.39785  1.58577
C  -4.89020  2.22186  2.97156
C  -4.00117  1.25145  3.46531
C  -3.25174  0.46265  2.58037
H  -4.40121  1.74278 -0.38190
H  -5.70877  3.15572  1.19023
H  -5.47157  2.83998  3.66401
H  -3.88446  1.11251  4.54574
H  -2.53639 -0.26939  2.96865
C   4.72756 -0.76579  1.46075
C   5.43882 -1.05626  2.63589
C   4.76030 -1.18232  3.85994
C   3.36639 -1.01887  3.90287
C   2.65446 -0.72321  2.72872
H   5.26954 -0.67002  0.51413
H   6.52598 -1.18652  2.59442
H   5.31759 -1.40915  4.77538
H   2.83002 -1.12028  4.85304
H   1.56261 -0.59490  2.73853
C   3.25194  0.46148 -2.58081
C   4.00123  1.25005 -3.46607
C   4.88971  2.22117 -2.97273
C   5.02273  2.39810 -1.58702
C   4.28170  1.60084 -0.69826
H   2.53703 -0.27114 -2.96880
H   3.88483  1.11038 -4.54644
H   5.47098  2.83911 -3.66543
H   5.70744  3.15653 -1.19180
H   4.40013  1.74401  0.38092
```

8ccc-IMe trans IMe4

```
SCF  =          -1956.77282011
H(0 K)=         -1956.061021
G(298 K)=       -1956.145537
SCF(C6H6) =     -1956.77980732
```

```
BP86(D3BJ) =    -1957.05201336
Low Freq. = 13.3468cm-1, 23.4979cm-1

91
C  -3.96616 -1.44179 -1.68462
C  -2.61216 -1.25266 -1.34903
C  -1.64640 -1.76533 -2.23688
C  -1.98921 -2.37847 -3.44786
C  -3.34887 -2.54459 -3.76353
C  -4.33545 -2.09118 -2.87543
P  -1.98851 -0.14496  0.05661
C  -3.43719  1.04062  0.24085
O  -0.28073 -1.55384 -1.91735
C   0.47829 -2.65262 -1.44867
C   1.59496 -2.36058 -0.63409
C   2.37315 -3.44310 -0.17027
C   2.06648 -4.76901 -0.50709
C   0.94692 -5.02970 -1.31047
C   0.14669 -3.97757 -1.78078
P   1.88839 -0.58893 -0.08836
C   2.42207 -0.90717  1.69737
Ru  0.03798  0.54029 -0.95522
C   0.35421  2.38741 -0.33072
N   0.48329  2.88460  0.96676
C   0.74745  4.26145  0.98793
C   0.78315  4.67209 -0.32168
N   0.53441  3.53311 -1.10576
C   0.37622  2.07472  2.17046
C   0.52924  3.54520 -2.56548
C  -2.26609 -1.25743  1.55296
C   3.54297 -0.14263 -0.85759
H   1.47709  3.14711 -2.96645
H   0.38072  4.57718 -2.91710
H  -0.28096  2.89488 -2.92410
C   1.02104  6.03288 -0.89602
C   0.92322  5.02837  2.25967
H  -0.34432  2.52501  2.87485
H   1.35320  1.96819  2.67286
H   0.02798  1.07822  1.86277
H   3.21803 -3.23232  0.49366
H   2.68628 -5.58978 -0.13279
H   0.68026 -6.05875 -1.57320
H  -0.73426 -4.19251 -2.38802
H  -0.98579  1.16149 -2.07053
H   1.03542  0.90463 -2.17651
H  -1.20183 -2.69906 -4.13592
H  -3.62977 -3.01704 -4.71025
H  -5.39516 -2.21535 -3.12100
H  -4.74029 -1.03960 -1.02321
H   0.00855  5.02328  2.88182
H   1.74129  4.62103  2.88188
H   1.16560  6.08022  2.04160
H   1.88627  6.05370 -1.58377
H   0.14707  6.41356 -1.45732
H   1.22619  6.75152 -0.08704
C   3.91086  1.22091 -0.82566
C   5.12884  1.64796 -1.37549
C   5.98822  0.72161 -1.99107
C   5.62247 -0.63182 -2.04539
C   4.41184 -1.06346 -1.47524
H   3.22366  1.95037 -0.38292
H   5.40109  2.70858 -1.33626
H   6.93342  1.05541 -2.43249
H   6.28061 -1.35978 -2.53261
H   4.14072 -2.12235 -1.52702
C   3.69680 -0.57781  2.20319
C   3.99362 -0.76111  3.56591
C   3.02603 -1.28266  4.43798
C   1.75340 -1.61685  3.94134
C   1.45166 -1.42236  2.58615
H   4.46427 -0.18009  1.53190
H   4.98956 -0.50068  3.94177
H   3.26006 -1.42774  5.49824
H   0.98702 -2.02454  4.60930
H   0.45179 -1.67175  2.21346
C  -2.28468 -0.68397  2.84634
C  -2.34279 -1.49107  3.99237
C  -2.36863 -2.89168  3.87073
C  -2.34377 -3.47374  2.59335
C  -2.29643 -2.66494  1.44543
H  -2.26722  0.40615  2.95631
H  -2.36945 -1.02467  4.98350
H  -2.41228 -3.52273  4.76467
H  -2.36845 -4.56376  2.48589
H  -2.28639 -3.13460  0.45641
C  -4.59931  0.74864  0.98843
C  -5.64623  1.68175  1.07220
C  -5.54610  2.91701  0.41163
C  -4.39295  3.21567 -0.33266
C  -3.34441  2.28584 -0.41562
H  -4.68429 -0.20719  1.51557
H  -6.54193  1.44095  1.65553
H  -6.36257  3.64420  0.47925
H  -4.30675  4.17842 -0.84859
H  -2.43944  2.50168 -0.99402
```

8ccc-IMe4 trans H

```
SCF  =          -1956.77434997
H(0 K)=         -1956.063504
G(298 K)=       -1956.148971
SCF(C6H6) =     -1956.78160744
BP86(D3BJ) =    -1957.06279461
Low Freq. = 12.9641cm-1, 21.5357cm-1

91
N  -3.09853 -2.15559 -1.25073
C  -2.11292 -1.91854 -0.31525
N  -2.57166 -2.60627  0.79661
C  -3.78673 -3.26620  0.55651
C  -4.12555 -2.97080 -0.74304
Ru -0.28988 -1.02283 -0.33237
P   2.02316 -0.75058 -0.18682
C   2.72195 -1.79552  1.21989
C  -1.78018 -2.67867  2.01573
C  -3.09885 -1.58737 -2.59196
C   2.79483  0.92366  0.18125
C   2.21625  1.69810  1.21386
C   2.60893  3.02263  1.45821
C   3.61127  3.59779  0.66241
C   4.21309  2.85107 -0.36121
C   3.80368  1.52811 -0.59556
O   1.27563  1.04839  2.01361
C   0.19768  1.77689  2.51946
C  -0.95578  1.97806  1.72131
C  -2.02950  2.67465  2.31977
C  -1.96815  3.12810  3.64826
C  -0.82412  2.88438  4.42290
C   0.26396  2.20411  3.85456
P  -1.03481  1.18726 -0.00627
C  -0.27569  2.57121 -1.03726
C  -2.85955  1.47178 -0.40259
C   3.10293 -1.35122 -1.60207
H  -2.13621 -1.07281 -2.72164
H  -3.92258 -0.86257 -2.71060
H  -3.19640 -2.38198 -3.35129
C  -5.31799 -3.36185 -1.55566
C  -4.48280 -4.09492  1.58837
H  -1.15579 -1.76767  2.07701
H  -1.11832 -3.56169  2.02517
H  -2.43498 -2.70392  2.90186
H   4.26528  0.95637 -1.40607
H   4.99592  3.29680 -0.98332
H   3.91985  4.63181  0.84901
H   2.13202  3.58996  2.26269
H   1.17115  1.99235  4.42884
H  -0.77375  3.22143  5.46344
H  -2.82020  3.66627  4.07691
H  -2.93767  2.85262  1.73596
H   0.18109 -2.52735 -0.80356
H  -0.33531 -0.80171 -1.87644
H  -4.76401 -3.50579  2.48148
H  -5.40953 -4.52019  1.17237
H  -3.85916 -4.93902  1.93625
```

```
H  -5.99423 -3.99681 -0.96199
H  -5.89654 -2.48146 -1.89085
H  -5.03729 -3.93260 -2.46003
C  -3.82948  0.75520  0.33581
C  -5.19300  0.84938  0.02048
C  -5.61564  1.64848 -1.05677
C  -4.66265  2.35766 -1.80350
C  -3.29733  2.27515 -1.47595
H  -3.51066  0.11457  1.16464
H  -5.92639  0.29250  0.61413
H  -6.67954  1.71926 -1.30778
H  -4.97898  2.98678 -2.64303
H  -2.56916  2.84234 -2.06302
C  -0.39445  3.93445 -0.69344
C   0.12123  4.92971 -1.53843
C   0.75858  4.57576 -2.73957
C   0.88399  3.22168 -3.08785
C   0.37272  2.22557 -2.23931
H  -0.89303  4.22018  0.23885
H   0.02460  5.98444 -1.25768
H   1.16041  5.35348 -3.39818
H   1.38722  2.93707 -4.01841
H   0.47595  1.16408 -2.49106
C   4.46995 -1.66140 -1.43644
C   5.23471 -2.11170 -2.52429
C   4.64196 -2.26018 -3.78981
C   3.28038 -1.96345 -3.96058
C   2.51446 -1.51537 -2.87151
H   4.93561 -1.55985 -0.45007
H   6.29406 -2.35226 -2.38189
H   5.23866 -2.61555 -4.63700
H   2.80894 -2.09116 -4.94135
H   1.44315 -1.31363 -2.98057
C   2.28841 -3.13643  1.32560
C   2.77422 -3.96550  2.34883
C   3.69302 -3.46686  3.28765
C   4.12853 -2.13598  3.19084
C   3.64921 -1.30641  2.16261
H   1.56495 -3.51583  0.59474
H   2.43395 -5.00531  2.41226
H   4.06919 -4.11275  4.08850
H   4.84766 -1.73896  3.91608
H   4.00240 -0.27258  2.09251
```

(ii) Ru-P dissociation

8ccc-P trans IMe4

```
SCF  =          -2340.22942530
H(0 K)=         -2339.340766
G(298 K)=       -2339.444599
SCF(C6H6) =     -2340.23732936ctc
BP86(D3BJ) =    -2340.55676224
Low Freq. = 6.9295cm-1, 13.1773cm-1
```

```
112
C   6.30604  1.02858  0.67166
C   4.94329  1.12976  1.02204
C   4.53333  2.21061  1.83578
C   5.45368  3.17729  2.26680
C   6.80808  3.06729  1.90889
C   7.23076  1.98946  1.11524
P   3.61928 -0.11869  0.56278
C   2.92570  0.67345 -0.99152
C   1.70162  0.18671 -1.50788
C   1.10438  0.74481 -2.65170
C   1.73684  1.81185 -3.30458
C   2.95017  2.32295 -2.81044
C   3.53107  1.76054 -1.66277
O   1.03581 -0.82508 -0.81070
C   0.67810 -1.98075 -1.51902
C  -0.57345 -2.57999 -1.25173
C  -0.85093 -3.80149 -1.90470
C   0.05676 -4.39011 -2.79788
C   1.28390 -3.76064 -3.05553
C   1.59906 -2.55726 -2.41135
P  -1.85514 -1.71566 -0.16128
C  -1.54750 -2.57837  1.49079
C  -0.54531 -3.54683  1.70650
C  -0.32743 -4.08130  2.98881
C  -1.11685 -3.66538  4.07250
C  -2.12618 -2.70729  3.86749
C  -2.33183 -2.16279  2.59085
Ru -1.92701  0.58506 -0.37596
C  -1.96857  1.13132  1.64401
N  -1.03551  0.88412  2.65130
C  -1.44343  1.35659  3.90666
C  -2.67331  1.93839  3.71625
N  -2.96862  1.79950  2.35099
C   0.23938  0.21898  2.42419
C  -0.62144  1.18294  5.14374
C  -3.59760  2.60667  4.68360
C  -4.19954  2.29277  1.75025
C  -2.02387  2.48404 -1.12215
N  -2.91590  3.03783 -2.03490
C  -2.58077  4.35419 -2.38358
C  -1.43986  4.66554 -1.68354
N  -1.12823  3.53085 -0.92002
C  -4.05059  2.31830 -2.59592
C   0.05359  3.41659 -0.07780
C  -0.61824  5.91485 -1.65787
C  -3.38879  5.16916 -3.34235
C  -3.41060 -2.63031 -0.71873
C  -4.07569 -2.15351 -1.86971
C  -5.24191 -2.78084 -2.33242
C  -5.77176 -3.88725 -1.64598
C  -5.11976 -4.36681 -0.49980
C  -3.94313 -3.74704 -0.04200
H  -3.48187  0.48450 -0.39164
H  -1.99813  0.14081 -2.00052
H   0.12680  0.36491 -2.96850
H   1.26988  2.25398 -4.19088
H   3.44613  3.15765 -3.31719
H   4.47357  2.16213 -1.27713
H  -1.81331 -4.28818 -1.72005
H  -0.19790 -5.33359 -3.29164
H   2.00530 -4.20727 -3.74788
H   2.56354 -2.06924 -2.57746
H   1.08659  0.89693  2.63188
H   0.33631 -0.67741  3.05876
H   0.27977 -0.09313  1.37078
H  -4.15270  2.03759  0.68213
H  -5.08295  1.80924  2.20411
H  -4.28899  3.38665  1.87496
H  -4.05698  2.40899 -3.69553
H  -3.92415  1.26362 -2.30932
H  -5.00804  2.70380 -2.20048
H   0.19056  4.33429  0.51867
H  -0.11425  2.56447  0.59899
H   0.96246  3.23460 -0.67805
H  -3.66089 -1.27912 -2.38313
H  -5.74359 -2.40022 -3.22948
H  -6.68755 -4.37158 -2.00252
H  -5.52300 -5.22968  0.04225
H  -3.44024 -4.13646  0.84860
H   0.06931 -3.88782  0.86715
H   0.45870 -4.83028  3.13743
H  -0.94960 -4.08560  5.07034
H  -2.75123 -2.37906  4.70568
H  -3.10091 -1.39739  2.43817
H   3.48183  2.29056  2.13701
H   5.11584  4.01053  2.89256
H   7.53082  3.81442  2.25358
H   8.28607  1.89381  0.83688
H   6.64663  0.19261  0.05262
H  -0.44839  0.11578  5.37519
H   0.37187  1.66159  5.05803
H  -1.13140  1.63320  6.01018
H  -4.58003  2.10184  4.74089
H  -3.16304  2.59780  5.69574
H  -3.79223  3.66222  4.41631
H  -2.95441  6.17559 -3.44952
H  -3.42562  4.71576 -4.35045
H  -4.43469  5.29342 -3.00524
```

```
H   0.43071  5.72791 -1.95311
H  -1.03151  6.65780 -2.35823
H  -0.59551  6.38419 -0.65624
C   4.64767 -1.50490 -0.16654
C   5.17463 -1.50239 -1.47650
C   5.93851 -2.58674 -1.93899
C   6.18691 -3.68525 -1.09907
C   5.66600 -3.69811  0.20486
C   4.89673 -2.61755  0.66528
H   4.98257 -0.64829 -2.13511
H   6.34084 -2.57279 -2.95794
H   6.77998 -4.53124 -1.46231
H   5.85006 -4.55483  0.86195
H   4.47761 -2.63727  1.67790
```

5cct-P

```
SCF  =           -2340.21980500
H(0 K)=          -2339.330387
G(298 K)=        -2339.429324
SCF(C6H6) =      -2340.22773529
BP86(D3BJ) =     -2340.56063825
```

```
112
C   4.16800 -3.07738 -0.23451
C   3.44960 -2.17626 -1.05154
C   2.92430 -2.63795 -2.27646
C   3.11891 -3.96900 -2.68242
C   3.83819 -4.85491 -1.86528
C   4.36227 -4.40738 -0.64009
P   3.14307 -0.37611 -0.62712
C   2.93078 -0.50896  1.23891
C   1.68125 -0.91682  1.75765
C   1.44466 -1.01949  3.13798
C   2.46927 -0.70926  4.04133
C   3.71909 -0.28526  3.55844
C   3.93863 -0.18373  2.17523
O   0.63689 -1.21992  0.86347
C   0.06772 -2.50101  1.00239
C  -1.30361 -2.66240  0.72766
C  -1.83922 -3.96827  0.79089
C  -1.04996 -5.06971  1.14541
C   0.31481 -4.87870  1.41950
C   0.87900 -3.59974  1.34273
P  -2.31311 -1.17765  0.19052
C  -3.72690 -1.12498  1.42535
C  -3.81206 -1.94123  2.57068
C  -4.85907 -1.77428  3.49388
C  -5.84336 -0.79746  3.28091
C  -5.76836  0.02266  2.14177
C  -4.71451 -0.13264  1.22908
Ru -0.88078  0.61210  0.08230
C  -2.21697  1.92495 -0.51674
N  -2.91047  2.00761 -1.73375
C  -3.77151  3.11353 -1.78745
C  -3.63504  3.77608 -0.59399
N  -2.70249  3.05164  0.16321
C  -2.74638  1.08906 -2.85087
C  -4.63147  3.41223 -2.97439
C  -4.29965  5.01967 -0.09443
C  -2.31919  3.43633  1.51367
C   0.52071  2.14396  0.14477
N   1.14359  2.75346  1.23392
C   1.96604  3.82268  0.85765
C   1.87574  3.91721 -0.51174
N   1.00969  2.89740 -0.92152
C   0.96557  2.36454  2.62708
C   0.68447  2.65983 -2.32258
C   2.52607  4.86830 -1.46644
C   2.74568  4.63524  1.84300
C  -3.16501 -1.99847 -1.28927
C  -2.35658 -2.25445 -2.41853
C  -2.89383 -2.86574 -3.55979
C  -4.25533 -3.21827 -3.59801
C  -5.06820 -2.96229 -2.48382
C  -4.52567 -2.36205 -1.33206
H  -1.24263  0.86370  1.71990
H  -0.37810  0.20263 -1.49024
H   0.45473 -1.33178  3.48254
H   2.28619 -0.78809  5.11793
H   4.52735 -0.04118  4.25590
H   4.91544  0.14327  1.80574
H  -2.89705 -4.11238  0.54671
H  -1.49155 -6.07024  1.19400
H   0.95285 -5.73034  1.67745
H   1.94605 -3.45214  1.52368
H  -2.62417  1.65471 -3.79085
H  -1.84042  0.49700 -2.63508
H  -3.61052  0.41053 -2.94927
H  -1.91862  2.52688  1.99375
H  -1.54105  4.22125  1.50484
H  -3.19957  3.80932  2.06213
H   1.88679  1.91490  3.03300
H   0.69489  3.24569  3.23516
H   0.14509  1.62542  2.64167
H   0.24492  1.64761 -2.36565
H  -0.04417  3.40249 -2.69323
H   1.60005  2.71229 -2.93446
H  -1.30387 -1.94528 -2.39260
H  -2.25174 -3.06125 -4.42618
H  -4.67820 -3.68858 -4.49252
H  -6.12954 -3.23482 -2.50339
H  -5.16830 -2.17833 -0.46518
H  -3.05380 -2.70965  2.75018
H  -4.90466 -2.41562  4.38131
H  -6.66133 -0.67176  3.99848
H  -6.52827  0.79278  1.96782
H  -4.64788  0.53103  0.35965
H   2.35116 -1.94838 -2.90629
H   2.70197 -4.31473 -3.63436
H   3.98684 -5.89378 -2.17865
H   4.92350 -5.09617  0.00114
H   4.57602 -2.73292  0.72241
H  -5.31048  2.57414 -3.21706
H  -5.25551  4.29872 -2.77962
H  -4.03833  3.62010 -3.88484
H  -4.95307  5.44042 -0.87500
H  -4.92869  4.83557  0.79689
H  -3.56776  5.80093  0.18224
H   3.33182  5.40831  1.32141
H   2.09305  5.15118  2.57146
H   3.45270  4.01560  2.42441
H   1.78413  5.40375 -2.08622
H   3.10591  5.62558 -0.91528
H   3.22120  4.35724 -2.15813
C   4.87873  0.33609 -0.72363
C   5.00629  1.72627 -0.48846
C   6.24784  2.36595 -0.62051
C   7.38120  1.63578 -1.01760
C   7.26201  0.26157 -1.27431
C   6.02304 -0.38517 -1.12451
H   4.12331  2.31176 -0.20084
H   6.32861  3.44068 -0.42329
H   8.34824  2.13704 -1.13211
H   8.13799 -0.31605 -1.58984
H   5.94802 -1.45847 -1.32370
```

3. Ru(DPEphos)$_2$H$_2$

5ccc

```
SCF  =           -3050.34279863
H(0 K)=          -3049.290751
G(298 K)=        -3049.398271
SCF(C6H6) =      -3050.34972720
Low Freq. = 15.3771cm-1, 18.5766cm-1
```

```
137
C  -3.46097  1.19741 -1.58729
C  -2.44976  2.18265 -1.57322
C  -2.86677  3.53069 -1.58584
C  -4.22586  3.88201 -1.59017
C  -5.20560  2.87918 -1.58128
C  -4.82562  1.53042 -1.58307
```

P -0.63671 1.65001 -1.44986
C -0.30538 1.21801 -3.25755
C -1.23057 1.44187 -4.29887
C -0.91237 1.09614 -5.62375
C   0.33807 0.53485 -5.92920
C   1.26893 0.31360 -4.90070
C   0.94431 0.64529 -3.57571
Ru 0.02308 0.19892 0.38877
P   2.17340 0.95547 1.05831
C   3.50873 1.95023 0.14210
C   4.77391 2.15634 0.73763
C   5.78485 2.84672 0.05198
C   5.55135 3.33602 -1.24459
C   4.30283 3.12607 -1.84809
C   3.29213 2.43622 -1.15726
P -2.12898 -0.29600 1.23538
C -3.23805 1.14669 1.79060
C -4.59801 0.92858 2.10621
C -5.39103 1.96296 2.62475
C -4.84211 3.23764 2.83969
C -3.49437 3.46717 2.52817
C -2.70176 2.42985 2.00774
C -2.02865 -1.19377 2.89248
C -1.73335 -2.57355 2.93675
C -1.60159 -3.24341 4.16347
C -1.74288 -2.54108 5.37132
C -2.02078 -1.16585 5.33963
C -2.16974 -0.49845 4.11177
C -3.47444 -1.33599 0.38556
C -3.76571 -1.08963 -0.97456
C -4.73872 -1.81895 -1.67375
C -5.47916 -2.80570 -1.00717
C -5.23045 -3.05760 0.34965
C -4.23780 -2.33436 1.03104
P   0.59532 -1.95838 -0.68834
C -0.77354 -2.85826 -1.62762
C -1.19874 -2.33229 -2.86852
C -2.15204 -3.00758 -3.64557
C -2.72726 -4.20235 -3.18190
C -2.35220 -4.70563 -1.92759
C -1.37946 -4.04271 -1.15966
C   1.99557 -2.25204 -1.97497
C   1.85527 -3.04877 -3.13308
C   2.92685 -3.27540 -4.01349
C   4.18208 -2.70133 -3.76422
C   4.35593 -1.90430 -2.62376
C   3.27843 -1.70435 -1.74585
C   1.17192 -3.36750 0.45644
C   1.23826 -3.17865 1.84753
C   1.67441 -4.21091 2.69718
C   2.04799 -5.45279 2.16563
C   1.99007 -5.65516 0.77560
C   1.56232 -4.62158 -0.06961
C   0.10537 3.39356 -1.42473
C   0.35818 4.13199 -2.60118
C   0.87862 5.43539 -2.53145
C   1.14025 6.02859 -1.28607
C   0.88428 5.30616 -0.10972
C   0.37798 3.99873 -0.18046
C   2.02469 1.97355 2.64287
C   1.28774 1.44519 3.72799
C   1.13435 2.17352 4.91728
C   1.68667 3.46002 5.03743
C   2.39629 4.00596 3.95841
C   2.57155 3.26694 2.77429
C   3.32991 -0.41822 1.61797
C   3.81215 -1.24839 0.58315
C   4.63480 -2.35408 0.83254
C   5.00318 -2.63094 2.15905
C   4.56337 -1.80589 3.20583
C   3.73068 -0.70492 2.93426
H   0.37388 -0.52370 1.78914
H -0.30440 1.47432 1.30494
H -1.60588 -3.13275 2.00501
H -1.37851 -4.31555 4.16979
H -1.63830 -3.06243 6.32885
H -2.13570 -0.60601 6.27440
H -2.40068 0.57047 4.10605
H -5.04557 -0.05712 1.94965
H -6.44295 1.76908 2.86202
H -5.46191 4.04500 3.24459
H -3.05127 4.45630 2.68646
H -4.89709 -1.60187 -2.73365
H -6.24484 -3.37189 -1.54747
H -5.80761 -3.81878 0.88512
H -4.05736 -2.54801 2.08796
H   0.95266 -2.20547 2.25925
H   1.72233 -4.03351 3.77652
H   2.38875 -6.25798 2.82589
H   2.28336 -6.61956 0.34608
H   1.53579 -4.79387 -1.14995
H -0.76357 -1.40113 -3.24148
H -2.44384 -2.59348 -4.61681
H -3.46869 -4.73168 -3.79015
H -2.80514 -5.62672 -1.54515
H -1.07200 -4.47243 -0.20196
H   0.88593 -3.49932 -3.35967
H   2.77208 -3.90170 -4.89840
H   5.01915 -2.86664 -4.45041
H   5.31066 -1.41895 -2.39985
O   3.45614 -0.82253 -0.68830
H   4.97322 -2.98326 0.00437
H   5.64311 -3.49414 2.36935
H   3.38503 -0.06836 3.75466
H   4.97458 1.76674 1.74118
H   6.75896 2.99683 0.53064
H   6.34140 3.87269 -1.78113
H   4.10747 3.49796 -2.85942
H   2.32615 2.27345 -1.63737
H   0.80942 0.46584 3.62684
H   0.56814 1.73605 5.74662
H   1.55766 4.03309 5.96194
H   2.82444 5.01176 4.03332
H   3.13484 3.70862 1.94819
H   0.14948 3.68851 -3.57957
H   1.07507 5.98895 -3.45642
H   1.54525 7.04489 -1.23340
H   1.09039 5.75210 0.86918
H   0.21095 3.42634 0.73775
H -2.20347 1.89054 -4.07508
H -1.64536 1.27120 -6.41927
H   0.58393 0.26702 -6.96248
H   2.24267 -0.13582 -5.12094
H   1.66000 0.43955 -2.77389
H -2.11442 4.32274 -1.57909
H -4.51308 4.93832 -1.59293
H -6.26908 3.14041 -1.57632
H -5.57592 0.73517 -1.57868
O -3.03037 -0.12438 -1.66237
H   4.86471 -2.01712 4.23698
H -1.64884 2.60864 1.77058

5ccc’ (from IRC)

SCF =   -3050.33423059
H(0 K)=   -3049.281869
G(298 K)=   -3049.390616
SCF(C6H6) =   -3050.34123065
BP86(D3BJ) =   -3050.84876609
Low Freq. = 11.8472cm-1, 19.8233cm-1

137
C -2.92139 0.47446 -2.59443
C -1.99480 1.54173 -2.56506
C -1.92538 2.33493 -3.73337
C -2.67866 2.03994 -4.88103
C -3.53002 0.92552 -4.89987
C -3.65392 0.14019 -3.74530
P -0.91283 1.82069 -1.01187
C   0.26646 3.12914 -1.71699
C   0.97782 2.86334 -2.90927
C   1.90050 3.78841 -3.42381
C   2.15431 4.99022 -2.74241
C   1.47074 5.25572 -1.54605

```
C   0.53108  4.34025 -1.04216
Ru -0.02783 -0.02243  0.35646
P   2.06471  0.64829  1.20030
C   3.68246  0.94068  0.26511
C   4.93047  0.43021  0.67918
C   6.09861  0.72220 -0.04444
C   6.04288  1.54292 -1.18172
C   4.80654  2.06321 -1.59755
C   3.63522  1.75411 -0.88671
P  -2.00828 -0.93443  1.23871
C  -2.05700 -0.66427  3.11351
C  -1.90035 -1.71801  4.03415
C  -1.89927 -1.46858  5.41896
C  -2.07665 -0.16626  5.90594
C  -2.24884  0.89100  4.99550
C  -2.22841  0.64578  3.61421
C  -2.30411 -2.79355  1.20977
C  -3.59767 -3.35350  1.16966
C  -3.77425 -4.74607  1.21715
C  -2.66268 -5.59696  1.31828
C  -1.37134 -5.04738  1.36355
C  -1.19122 -3.65660  1.30107
C  -3.73887 -0.30277  0.81944
C  -4.04455  0.01668 -0.52096
C  -5.27571  0.58518 -0.88853
C  -6.24882  0.80928  0.09496
C  -5.99358  0.46152  1.43007
C  -4.74940 -0.08464  1.78145
P   0.81132 -1.61641 -1.34511
C   1.06996 -0.95741 -3.10781
C   2.23065 -0.21780 -3.42942
C   2.44075  0.26824 -4.73026
C   1.47405  0.06400 -5.72848
C   0.30019 -0.63707 -5.41150
C   0.10581 -1.15287 -4.11885
C   2.53750 -2.37929 -1.09413
C   3.37763 -2.72391 -2.18047
C   4.66771 -3.24082 -1.99534
C   5.15990 -3.43452 -0.69560
C   4.34157 -3.14958  0.40253
C   3.04007 -2.65343  0.19748
C  -0.11935 -3.20559 -1.80462
C   0.51867 -4.43776 -2.05785
C  -0.22470 -5.56231 -2.45297
C  -1.61611 -5.47363 -2.61094
C  -2.26167 -4.25406 -2.35514
C  -1.52113 -3.13380 -1.94468
C  -2.06740  3.00426 -0.08355
C  -3.20697  3.58913 -0.67334
C  -3.98595  4.51974  0.03442
C  -3.62939  4.90019  1.33698
C  -2.48828  4.33682  1.92942
C  -1.72154  3.39057  1.22908
C   2.00792  2.25113  2.20817
C   2.83720  3.35954  1.94701
C   2.73733  4.52944  2.72296
C   1.82547  4.60224  3.78516
C   1.00391  3.49563  4.06412
C   1.08604  2.33716  3.27768
C   2.71651 -0.51068  2.56660
C   2.65921 -1.92032  2.44244
C   3.00686 -2.77559  3.50067
C   3.48767 -2.24180  4.70476
C   3.62003 -0.85223  4.83449
C   3.23527 -0.00731  3.78071
H   0.31661 -1.11812  1.47275
H  -0.43273  0.96225  1.55374
H  -4.47433 -2.70194  1.10522
H  -4.78636 -5.16430  1.17979
H  -2.80142 -6.68284  1.35697
H  -0.49644 -5.70249  1.43154
H  -0.18146 -3.23302  1.31476
H  -1.77605 -2.74361  3.67606
H  -1.76674 -2.30375  6.11546
H  -2.08519  0.02560  6.98427
H  -2.40200  1.91283  5.36054
H  -5.46619  0.83948 -1.93463
H  -7.21088  1.24849 -0.18949
H  -6.75537  0.62050  2.19992
H  -4.55171 -0.33604  2.82694
H   1.60264 -4.52881 -1.94910
H   0.29137 -6.51069 -2.64043
H  -2.19398 -6.35084 -2.92167
H  -3.34941 -4.17489 -2.45589
H  -2.03697 -2.19741 -1.71492
H   2.98967 -0.03295 -2.66399
H   3.36361  0.81375 -4.95697
H   1.63488  0.44532 -6.74261
H  -0.46686 -0.80145 -6.17572
H  -0.79946 -1.72868 -3.90726
H   3.01411 -2.57318 -3.19971
H   5.28422 -3.48615 -2.86602
H   6.16991 -3.82587 -0.53396
H   4.68987 -3.32364  1.42465
O   2.17304 -2.51030  1.27560
H   2.89723 -3.85476  3.35586
H   3.76286 -2.90905  5.52806
H   3.32423  1.07403  3.91261
H   4.99635 -0.19587  1.57323
H   7.05708  0.30911  0.28876
H   6.95642  1.77506 -1.73976
H   4.74249  2.71141 -2.47812
H   2.67686  2.15771 -1.22596
H   3.56891  3.31803  1.13593
H   3.38680  5.38188  2.49538
H   1.75632  5.51006  4.39408
H   0.29391  3.53034  4.89784
H   0.43216  1.48696  3.50029
H  -3.49327  3.32212 -1.69422
H  -4.87352  4.95153 -0.44101
H  -4.23421  5.63093  1.88468
H  -2.18594  4.63099  2.94047
H  -0.83620  2.95324  1.70140
H   0.80781  1.92927 -3.44935
H   2.42065  3.56038 -4.36076
H   2.87468  5.71260 -3.14101
H   1.65655  6.18775 -1.00128
H  -0.00186  4.58233 -0.11893
H  -1.26171  3.20252 -3.75081
H  -2.59040  2.68283 -5.76295
H  -4.10460  0.67506 -5.79760
H  -4.31975 -0.72755 -3.71100
O  -3.07727 -0.32343 -1.46190
H   4.00973 -0.41708  5.76048
H  -2.36200  1.47935  2.91715
```

TS(5-9)ccc

```
SCF  =          -3050.28854574
H(0 K)=         -3049.239059
G(298 K)=       -3049.343606
SCF(C6H6) =     -3050.29520578
Low Freq. = -462.1166cm-1, 23.0332cm-1
```

```
137
C  -2.36720  1.28317 -2.80025
C  -1.12239  1.93643 -2.64783
C  -0.61292  2.57796 -3.79990
C  -1.28049  2.53188 -5.03474
C  -2.48843  1.83119 -5.16059
C  -3.03495  1.20379 -4.03213
P  -0.26000  1.92284 -0.93758
C   1.35142  2.80042 -1.37950
C   2.15409  2.35407 -2.45261
C   3.34633  3.01890 -2.78378
C   3.78174  4.11679 -2.02446
C   3.01863  4.53657 -0.92239
C   1.81159  3.89093 -0.60762
Ru -0.08495  0.00189  0.44709
P   2.14361  0.19029  1.36726
C   3.83514 -0.07990  0.59532
C   4.54736 -1.28291  0.77244
C   5.80838 -1.45874  0.17648
C   6.37582 -0.43397 -0.59699
```

```
C   5.67536  0.77282 -0.76929
C   4.41467  0.94862 -0.18020
P  -2.36923 -0.43228  0.95094
C  -2.69146 -0.56096  2.81544
C  -3.78878 -1.30412  3.30779
C  -4.06832 -1.34966  4.68304
C  -3.25787 -0.65410  5.59459
C  -2.16146  0.08030  5.11924
C  -1.87920  0.12227  3.74372
C  -3.25997 -2.00335  0.42175
C  -4.57756 -2.00149 -0.07917
C  -5.22419 -3.21147 -0.38455
C  -4.56576 -4.43435 -0.18359
C  -3.25329 -4.44200  0.31660
C  -2.59982 -3.23607  0.61135
C  -3.66580  0.86140  0.52101
C  -3.72593  1.31431 -0.81340
C  -4.57368  2.35890 -1.21056
C  -5.40770  2.95695 -0.25437
C  -5.39432  2.50718  1.07475
C  -4.52735  1.47008  1.45531
P   0.60844 -1.76386 -1.23377
C   1.53277 -1.19643 -2.78660
C   2.92216 -0.94101 -2.75684
C   3.60064 -0.51055 -3.90923
C   2.90092 -0.29711 -5.10757
C   1.51459 -0.51900 -5.14138
C   0.83898 -0.97081 -3.99561
C   1.83096 -3.04183 -0.58076
C   2.84666 -3.67723 -1.32869
C   3.71778 -4.59879 -0.72654
C   3.59230 -4.90063  0.64159
C   2.57498 -4.30637  1.39979
C   1.68440 -3.40571  0.77967
C  -0.61833 -2.91633 -2.09485
C  -0.30877 -4.25207 -2.42471
C  -1.21821 -5.03661 -3.15193
C  -2.44584 -4.49738 -3.56755
C  -2.76591 -3.17226 -3.23523
C  -1.86308 -2.39114 -2.49530
C  -1.17617  3.43735 -0.23390
C  -1.35015  4.58885 -1.03502
C  -1.95885  5.74075 -0.51738
C  -2.41187  5.76384  0.81270
C  -2.25529  4.62422  1.61380
C  -1.64161  3.47150  1.09217
C   2.57310  1.72680  2.38927
C   3.89126  1.95912  2.84527
C   4.18537  3.05979  3.66556
C   3.17148  3.95500  4.04266
C   1.86075  3.73887  3.59124
C   1.56838  2.63516  2.77308
C   2.00204 -1.12033  2.68595
C   0.79429 -1.92973  2.56656
C   0.30440 -2.53111  3.77653
C   1.00915 -2.42799  4.97840
C   2.24197 -1.75096  5.04558
C   2.70366 -1.07997  3.89719
H  -0.04235 -1.04619  1.91131
H  -0.37931  1.03872  1.63123
H  -5.11041 -1.05571 -0.21738
H  -6.24889 -3.19430 -0.77241
H  -5.07188 -5.37709 -0.41779
H  -2.72778 -5.39083  0.46746
H  -1.56821 -3.24939  0.97964
H  -4.43442 -1.84891  2.61319
H  -4.92439 -1.93285  5.03972
H  -3.47458 -0.69281  6.66760
H  -1.50750  0.61196  5.81842
H  -4.57630  2.69277 -2.25184
H  -6.07238  3.77318 -0.55583
H  -6.05139  2.96686  1.81985
H  -4.50894  1.13626  2.49638
H   0.64561 -4.68713 -2.11521
H  -0.96123 -6.07335 -3.39646
H  -3.15242 -5.10991 -4.13810
H  -3.72927 -2.74623 -3.53404
H  -2.13631 -1.37387 -2.20581
H   3.48730 -1.08465 -1.83324
H   4.68081 -0.33746 -3.85754
H   3.42982  0.03683 -6.00687
H   0.95154 -0.35282 -6.06597
H  -0.23626 -1.16284 -4.05362
H   2.96558 -3.44462 -2.39074
H   4.49447 -5.08213 -1.32834
H   4.27489 -5.61651  1.11250
H   2.43253 -4.54374  2.45815
O   0.58440 -2.92027  1.43223
H  -0.61175 -3.12679  3.71860
H   0.60740 -2.92237  5.87067
H   3.58049 -0.42757  3.97844
H   4.11668 -2.08382  1.38050
H   6.34511 -2.40208  0.32280
H   7.36057 -0.57092 -1.05705
H   6.10905  1.58494 -1.36270
H   3.88758  1.89742 -0.31239
H   4.69948  1.28298  2.55159
H   5.21423  3.21761  4.00742
H   3.40329  4.81597  4.67887
H   1.05968  4.43368  3.86628
H   0.54852  2.47451  2.41346
H  -1.00399  4.58994 -2.07316
H  -2.07842  6.62313 -1.15562
H  -2.88810  6.66328  1.21771
H  -2.61588  4.62242  2.64797
H  -1.52942  2.57954  1.71530
H   1.84662  1.49005 -3.04609
H   3.93558  2.66221 -3.63484
H   4.71103  4.63579 -2.28282
H   3.35400  5.37855 -0.30757
H   1.22242  4.24770  0.24121
H   0.33150  3.12352 -3.73685
H  -0.84650  3.04443 -5.89956
H  -3.00708  1.77753 -6.12319
H  -3.97906  0.65291 -4.08216
O  -2.92933  0.61320 -1.71336
H   2.79655 -1.68485  5.98627
H  -1.00652  0.67528  3.38403
```

9ccc (from IRC)

```
SCF  =           -3050.37443468
H(0 K)=          -3049.319992
G(298 K)=        -3049.427895
SCF(C6H6) =      -3050.38156243
BP86(D3BJ) =     -3050.90086685
Low Freq. = 10.7689cm-1, 16.8291cm-1

137
C  -2.55169  0.72861 -2.82725
C  -1.17694  0.99281 -3.01583
C  -0.72497  1.01443 -4.35446
C  -1.58977  0.76816 -5.43428
C  -2.94453  0.48852 -5.20783
C  -3.42706  0.46361 -3.89172
P  -0.07003  1.40667 -1.50260
C   1.56338  1.63011 -2.42702
C   2.10941  0.53365 -3.13122
C   3.29755  0.67023 -3.86797
C   3.98422  1.89530 -3.88203
C   3.47184  2.98007 -3.15302
C   2.26718  2.85302 -2.44090
Ru -0.01411  0.04767  0.40821
P   2.32283  0.39397  0.98487
C   3.70907 -0.72952  0.39377
C   3.99355 -1.92714  1.09034
C   5.00860 -2.78916  0.64498
C   5.75540 -2.47493 -0.50328
C   5.46156 -1.30214 -1.21669
C   4.44051 -0.44224 -0.77886
P  -2.28425  0.26128  1.22053
C  -2.30018  0.74213  3.05204
C  -3.02852  0.01412  4.01524
C  -3.06497  0.42996  5.35745
```

C -2.39104 1.59169 5.76096
C -1.67006 2.33299 4.81040
C -1.61993 1.90553 3.47424
C -3.48327 -1.18307 1.27920
C -4.84411 -1.05321 0.93655
C -5.71269 -2.15168 1.04584
C -5.23557 -3.38742 1.50907
C -3.88248 -3.52189 1.86010
C -3.00919 -2.43023 1.74071
C -3.40647 1.61429 0.55602
C -3.58068 1.67324 -0.84297
C -4.30266 2.70053 -1.46697
C -4.90504 3.68258 -0.66639
C -4.79438 3.62336 0.73175
C -4.04861 2.59704 1.33504
P 0.04517 -2.19582 -0.62397
C 1.04018 -2.69830 -2.14009
C 2.42110 -2.97565 -2.07329
C 3.13930 -3.30957 -3.23450
C 2.49698 -3.35808 -4.48127
C 1.12314 -3.07338 -4.55979
C 0.40067 -2.75228 -3.40069
C 0.79687 -3.11630 0.79041
C 1.43098 -4.37558 0.74610
C 1.87134 -5.00505 1.91934
C 1.65331 -4.36930 3.15964
C 1.03296 -3.11909 3.22959
C 0.60857 -2.44732 2.04302
C -1.47492 -3.25048 -1.03811
C -1.46696 -4.65148 -0.87209
C -2.56308 -5.42630 -1.28002
C -3.68543 -4.81526 -1.86326
C -3.70796 -3.42186 -2.01989
C -2.61188 -2.64583 -1.60561
C -0.62242 3.22241 -1.39356
C -0.69929 4.03353 -2.54805
C -1.09411 5.37631 -2.45646
C -1.43229 5.93021 -1.20998
C -1.37638 5.13008 -0.05944
C -0.97283 3.78737 -0.15552
C 3.08338 2.11652 0.78037
C 4.46814 2.34703 0.63589
C 4.97948 3.65473 0.62382
C 4.12271 4.75674 0.77261
C 2.74723 4.53789 0.94389
C 2.23793 3.22985 0.94870
C 2.63119 0.33691 2.85479
C 1.58042 0.56116 3.76329
C 1.83527 0.65648 5.14187
C 3.14276 0.52961 5.63350
C 4.19967 0.31678 4.73358
C 3.94711 0.22718 3.35644
H 0.55906 0.64050 3.39264
H 0.02705 1.35105 1.36646
H -5.23464 -0.09030 0.59441
H -6.76775 -2.03523 0.77417
H -5.91482 -4.24208 1.59716
H -3.49820 -4.48194 2.21925
H -1.95717 -2.53222 2.02052
H -3.57341 -0.88633 3.72259
H -3.63010 -0.16035 6.08678
H -2.42613 1.91823 6.80580
H -1.13574 3.24190 5.10697
H -4.38944 2.72469 -2.55669
H -5.47199 4.48972 -1.14181
H -5.28266 4.37840 1.35600
H -3.95276 2.56678 2.42362
H -0.60696 -5.14347 -0.40977
H -2.53820 -6.51297 -1.14110
H -4.53984 -5.42157 -2.18320
H -4.58467 -2.93012 -2.45471
H -2.65321 -1.55999 -1.69525
H 2.94043 -2.94003 -1.11295
H 4.20874 -3.53037 -3.15301
H 3.05872 -3.62268 -5.38375
H 0.60614 -3.11110 -5.52485
H -0.67300 -2.55504 -3.47669
H 1.60527 -4.85724 -0.22280
H 2.37560 -5.97529 1.87017
H 1.98698 -4.85295 4.08537
H 0.88473 -2.60934 4.18649
O 0.05714 -1.24236 2.14384
H 0.99765 0.82638 5.82688
H 3.33992 0.59958 6.70891
H 4.78677 0.07350 2.67266
H 3.42710 -2.18852 1.98905
H 5.21132 -3.70918 1.20277
H 6.55672 -3.14151 -0.84026
H 6.02587 -1.04865 -2.12066
H 4.22419 0.46147 -1.35341
H 5.16084 1.50779 0.53489
H 6.05756 3.80850 0.50353
H 4.52531 5.77534 0.76381
H 2.06158 5.38306 1.06761
H 1.16761 3.06261 1.08635
H -0.44647 3.61557 -3.52756
H -1.14014 5.99044 -3.36249
H -1.74483 6.97763 -1.13942
H -1.65481 5.54414 0.91533
H -0.94132 3.15383 0.73525
H 1.59884 -0.43262 -3.12448
H 3.68393 -0.19352 -4.41838
H 4.91337 2.00252 -4.45221
H 4.00399 3.93703 -3.13977
H 1.88081 3.71521 -1.89277
H 0.32625 1.22561 -4.56293
H -1.19465 0.79396 -6.45509
H -3.62152 0.28580 -6.04390
H -4.47281 0.23175 -3.66814
O -3.03673 0.59831 -1.52814
H 5.22784 0.22485 5.10052
H -1.03476 2.48215 2.75137

5cct (lowest conf)

SCF = -3050.31565805
H(0 K)= -3049.265430
G(298 K)= -3049.372250
SCF(C6H6) = -3050.32270708
Low Freq. = 13.9902cm-1, 15.9991cm-1

137
C 2.02331 -4.17123 1.29201
C 1.33263 -2.95761 1.50270
C 0.26296 -2.94542 2.42410
C -0.08362 -4.10431 3.13653
C 0.60954 -5.30570 2.91962
C 1.65758 -5.33690 1.98716
P 1.84836 -1.31934 0.70522
C 2.94995 -0.73543 2.15654
C 3.19159 -1.54135 3.29341
C 3.99791 -1.10613 4.35868
C 4.59176 0.16313 4.32258
C 4.37664 0.98610 3.20905
C 3.57961 0.53115 2.14609
C 3.22410 -1.90089 -0.45498
C 2.99915 -1.81865 -1.84080
C 3.96604 -2.27928 -2.75016
C 5.17088 -2.82538 -2.28306
C 5.41260 -2.89805 -0.90064
C 4.44871 -2.43374 0.00746
Ru 0.02131 0.01352 -0.01333
P -1.82790 1.24767 0.86057
C -1.92521 1.38881 2.76204
C -0.80312 1.16536 3.58202
C -0.88677 1.30203 4.97873
C -2.10113 1.66046 5.58235
C -3.22916 1.88756 4.77709
C -3.14074 1.75765 3.38217
P 1.24267 1.92283 -0.73540
C 0.66821 2.68028 -2.38012
C 0.51383 1.83708 -3.50443
C 0.15182 2.35312 -4.75903
C -0.08395 3.72930 -4.91516

```
C   0.04760  4.57557 -3.80503
C   0.42323  4.05822 -2.55242
P  -1.30958 -1.74229 -0.91014
C  -2.72046 -1.19106 -2.04675
C  -3.84081 -2.00688 -2.30629
C  -4.80303 -1.61813 -3.25217
C  -4.65426 -0.41758 -3.96473
C  -3.53854  0.39759 -3.71847
C  -2.58391  0.01531 -2.76166
C   1.57801  3.48774  0.28603
C   2.44959  4.49917 -0.17567
C   2.75402  5.60548  0.63259
C   2.20419  5.71087  1.92147
C   1.35573  4.69962  2.39633
C   1.04889  3.59726  1.58177
C   3.04664  1.61887 -1.20212
C   3.61097  1.66365 -2.48986
C   4.97257  1.37507 -2.68886
C   5.79069  1.04202 -1.59852
C   5.25954  1.01889 -0.29977
C   3.90188  1.31455 -0.12337
C  -2.22796 -3.08625  0.09161
C  -2.26174 -4.45726 -0.25105
C  -2.86384 -5.41570  0.58101
C  -3.44759 -5.02903  1.79607
C  -3.46252 -3.67183  2.14921
C  -2.88646 -2.72399  1.28966
C  -0.40818 -2.84076 -2.16471
C  -0.46663 -2.54894 -3.54475
C   0.23993 -3.32597 -4.47749
C   1.00939 -4.42066 -4.05348
C   1.07817 -4.71915 -2.68451
C   0.38568 -3.93189 -1.75022
C  -2.12351  3.06429  0.40625
C  -2.21278  4.10686  1.35286
C  -2.50744  5.41992  0.95196
C  -2.73644  5.71448 -0.40097
C  -2.64331  4.68837 -1.35375
C  -2.32236  3.38032 -0.95467
C  -3.62256  0.66380  0.61705
C  -3.96159 -0.63663  1.04191
C  -5.26216 -1.14971  0.91185
C  -6.26561 -0.35175  0.34686
C  -5.96323  0.95037 -0.07503
C  -4.65778  1.44796  0.06586
H   0.15274 -0.04912 -1.67774
H   0.04854 -0.22851  1.62381
H  -3.96632 -2.95219 -1.76999
H  -5.67135 -2.26105 -3.43386
H  -5.40401 -0.11987 -4.70583
H  -3.40490  1.33501 -4.26952
H  -1.70804  0.64180 -2.56196
H  -1.07659 -1.71428 -3.90113
H   0.17269 -3.07944 -5.54300
H   1.54975 -5.03407 -4.78234
H   1.68079 -5.56360 -2.33330
H   0.46495 -4.17797 -0.68889
H  -1.80434 -4.78742 -1.18693
H  -2.86850 -6.46726  0.27579
H  -3.90325 -5.77280  2.45804
H  -3.92657 -3.32308  3.07712
O  -2.95143 -1.38030  1.65754
H  -5.47731 -2.16681  1.25069
H  -7.28080 -0.74936  0.24413
H  -6.73943  1.58609 -0.51306
H  -4.44325  2.46713 -0.26292
H  -2.05938  3.89690  2.41422
H  -2.56449  6.21303  1.70544
H  -2.97999  6.73643 -0.71092
H  -2.80580  4.90214 -2.41510
H  -2.24417  2.59176 -1.70845
H   0.13932  0.85496  3.11690
H   0.00231  1.11827  5.59172
H  -2.17083  1.75890  6.67115
H  -4.18571  2.16482  5.23370
H  -4.03066  1.94042  2.77209
H   2.98376  1.92033 -3.34807
H   5.39082  1.40881 -3.70009
H   6.84933  0.80921 -1.75293
H   5.88128  0.78012  0.56823
O   3.31633  1.43365  1.12886
H   2.90883  4.41499 -1.16627
H   3.43104  6.38099  0.25739
H   2.44711  6.57112  2.55479
H   0.93540  4.75957  3.40616
H   0.41878  2.79241  1.96862
H   0.67053  0.75899 -3.38707
H   0.05133  1.67514 -5.61367
H  -0.37112  4.13481 -5.89136
H  -0.14361  5.64968 -3.90559
H   0.51050  4.73992 -1.70366
H   2.73503 -2.53117  3.35642
H   4.15333 -1.76608  5.21849
H   5.21456  0.51385  5.15201
H   4.80414  1.99096  3.14261
H   4.65211 -2.48363  1.08241
H   6.35593 -3.31201 -0.52712
H   5.92472 -3.18478 -2.99217
H   3.77167 -2.21103 -3.82542
H   2.05886 -1.38463 -2.19780
H  -0.29410 -2.01546  2.58000
H  -0.91079 -4.06768  3.85230
H   0.33186 -6.21127  3.46956
H   2.20384 -6.26855  1.80227
H   2.85685 -4.21040  0.58551
```

5cct (from IRC)

```
SCF  =           -3050.30870370
H(0 K)=          -3049.258299
G(298 K)=        -3049.364005
SCF(C6H6) =      -3050.31642846
BP86(D3BJ) =     -3050.83591557
Low Freq. = 14.2336cm-1, 18.1403cm-1
```

```
137
C   4.46509  1.94984  0.40461
C   3.53980  1.17271 -0.31980
C   4.04099  0.07384 -1.04399
C   5.40762 -0.24065 -1.07520
C   6.30803  0.55007 -0.34702
C   5.83570  1.63983  0.39924
P   1.69124  1.56793 -0.52138
C   1.72206  3.21315  0.41139
C   1.81897  4.48893 -0.17874
C   1.91154  5.64105  0.61952
C   1.91078  5.54089  2.01920
C   1.79951  4.27624  2.61857
C   1.70118  3.12597  1.82013
O   3.10943 -0.65205 -1.78480
C   3.15272 -2.03957 -1.66344
C   2.54211 -2.67903 -0.55404
C   2.60900 -4.08938 -0.53599
C   3.25233 -4.81983 -1.54809
C   3.82623 -4.15746 -2.64092
C   3.76536 -2.75841 -2.70114
P   1.57722 -1.63456  0.71394
C   0.97766 -2.93344  1.96309
C  -0.33297 -2.84153  2.46484
C  -0.79170 -3.72437  3.45886
C   0.06254 -4.70856  3.97551
C   1.38804 -4.78433  3.51376
C   1.84576 -3.89708  2.52842
Ru -0.01679 -0.01199  0.02184
P  -1.60311  1.70788  0.53507
C  -1.40799  3.43650 -0.21668
C  -1.21113  3.50845 -1.61225
C  -1.16514  4.74322 -2.27852
C  -1.30655  5.93722 -1.55347
C  -1.47786  5.88002 -0.16171
C  -1.52908  4.64275  0.50192
P  -1.68829 -1.57538 -0.69476
C  -2.97937 -2.46213  0.43255
C  -3.59741 -1.73573  1.47779
```

```
C  -4.40433 -2.34574  2.45150
C  -4.68788 -3.71624  2.35885
C  -4.17021 -4.44960  1.28132
C  -3.32761 -3.82998  0.34304
C  -1.00981 -3.08621 -1.61153
C  -0.88592 -3.05443 -3.01720
C  -0.43744 -4.17866 -3.72736
C  -0.12007 -5.36560 -3.04915
C  -0.21179 -5.40104 -1.65039
C  -0.62987 -4.26581 -0.93573
C  -2.88291 -1.01872 -2.06415
C  -2.55364  0.07355 -2.88599
C  -3.34506  0.41005 -3.99680
C  -4.48812 -0.34404 -4.29999
C  -4.83515 -1.42959 -3.47933
C  -4.04055 -1.76493 -2.37253
C   1.90489  2.11714 -2.33383
C   2.95972  2.98552 -2.69850
C   3.14837  3.36752 -4.03593
C   2.30155  2.87090 -5.04041
C   1.27652  1.97746 -4.69530
C   1.08580  1.60527 -3.35399
C  -3.43655  1.50264  0.09832
C  -4.17561  2.35747 -0.74377
C  -5.53691  2.12803 -1.00226
C  -6.18900  1.03592 -0.41165
C  -5.48186  0.17780  0.44197
C  -4.12194  0.42263  0.68679
C  -1.91628  2.15069  2.36155
C  -3.03321  2.94212  2.71676
C  -3.28686  3.27103  4.05765
C  -2.43459  2.80832  5.07342
C  -1.33206  2.01021  4.73445
C  -1.08031  1.68759  3.39049
C   2.98322 -1.08762  1.86711
C   4.29334 -1.59566  1.77324
C   5.25148 -1.28896  2.75468
C   4.91148 -0.48517  3.85259
C   3.60265  0.01168  3.96342
C   2.64981 -0.28803  2.97906
H   0.00117 -0.04027  1.68709
H   0.04208 -0.22191 -1.63587
H   4.57379 -2.23378  0.93040
H   6.26805 -1.68518  2.65643
H   5.65867 -0.25081  4.61834
H   3.31956  0.63141  4.82140
H   1.62315  0.07920  3.07253
H  -0.97920 -2.04432  2.09102
H  -1.81995 -3.63162  3.82329
H  -0.29131 -5.39905  4.74878
H   2.07630 -5.52583  3.93419
H   2.89663 -3.93555  2.22653
H   2.13207 -4.63730  0.27892
H   3.28199 -5.91249 -1.48629
H   4.31441 -4.72046 -3.44314
H   4.18765 -2.19894 -3.54135
H   5.75058 -1.10020 -1.65865
H   7.37689  0.31208 -0.36577
H   6.53153  2.25794  0.97562
H   4.11298  2.80821  0.98222
H   1.79836  4.59588 -1.26553
H   1.97656  6.62299  0.13850
H   1.98630  6.44134  2.63833
H   1.78385  4.18085  3.70948
H   1.61898  2.14252  2.29029
H   3.65640  3.35144 -1.93826
H   3.97002  4.04509 -4.29290
H   2.45380  3.16338 -6.08508
H   0.62843  1.55525 -5.47140
H   0.32144  0.87061 -3.07764
H  -1.08856  2.58510 -2.18349
H  -1.01099  4.76636 -3.36258
H  -1.27300  6.90386 -2.06765
H  -1.56983  6.80444  0.41890
H  -3.72142  3.29556  1.94265
H  -4.16019  3.88369  4.30719
H  -2.63672  3.05852  6.12059
H  -0.66899  1.62500  5.51709
H  -0.24907  1.02909  3.11822
H  -3.68080  3.21257 -1.21055
H  -6.08313  2.80516 -1.66677
H  -7.25018  0.85010 -0.60784
H  -5.97285 -0.67630  0.91681
O  -3.38859 -0.36295  1.57948
H  -1.65958  0.65044 -2.63718
H  -3.06696  1.26623 -4.62117
H  -5.10772 -0.08635 -5.16576
H  -5.72900 -2.02304 -3.70106
H  -2.92425 -4.43434 -0.47257
H  -4.41025 -5.51210  1.16959
H  -5.32562 -4.19689  3.10798
H  -4.81459 -1.72340  3.25271
H  -0.67582 -4.31064  0.15616
H   0.04970 -6.31360 -1.10347
H   0.20471 -6.25134 -3.60534
H  -0.35463 -4.12790 -4.81847
H  -1.15935 -2.14987 -3.56731
H  -1.65275  4.62587  1.58716
H  -4.32587 -2.61745 -1.74965
```

TS(5-9)cct

```
SCF  =          -3050.25020474
H(0 K)=         -3049.202713
G(298 K)=       -3049.308167
SCF(C6H6) =     -3050.25735062
Low Freq. = -958.8278cm-1, 13.1310cm-1
```

```
137
C   4.84533 -1.28895 -0.23851
C   3.55638 -1.09286 -0.76650
C   3.07251 -2.00439 -1.73150
C   3.81818 -3.13087 -2.12035
C   5.08941 -3.32643 -1.55956
C   5.60524 -2.40333 -0.63284
P   2.37506  0.35719 -0.49168
C   3.39558  1.37155  0.72135
C   3.76674  2.71280  0.49320
C   4.51354  3.42347  1.44868
C   4.90698  2.80901  2.64615
C   4.53600  1.47661  2.88930
C   3.78108  0.76966  1.94113
O   1.90421 -1.62810 -2.34604
C   0.67632 -2.42977 -2.14663
C   0.53409 -3.29528 -0.97674
C   0.12342 -4.62809 -1.14895
C  -0.19580 -5.15310 -2.41127
C  -0.17212 -4.29377 -3.52928
C   0.25960 -2.97486 -3.40913
P   0.49263 -2.32567  0.59547
C  -0.65479 -3.18461  1.82826
C  -1.59370 -2.40178  2.52481
C  -2.41406 -2.97112  3.51491
C  -2.30594 -4.33576  3.81813
C  -1.34832 -5.12091  3.15222
C  -0.51627 -4.54693  2.18018
Ru  0.01986 -0.08197 -0.02403
P  -0.32618  2.26211  0.53011
C   0.63748  3.67639 -0.28415
C   0.66618  3.72323 -1.69331
C   1.30062  4.77620 -2.37236
C   1.92001  5.80702 -1.64807
C   1.89601  5.77451 -0.24456
C   1.26281  4.71883  0.43240
P  -2.32059 -0.41324 -0.56300
C  -3.72935 -0.46968  0.74200
C  -3.70667  0.38767  1.86780
C  -4.58842  0.23684  2.94974
C  -5.59010 -0.74381  2.89818
C  -5.69975 -1.55020  1.75628
C  -4.77780 -1.41756  0.70469
C  -2.82502 -1.95817 -1.53282
C  -3.26134 -1.88803 -2.87322
C  -3.73977 -3.03118 -3.53499
```

```
C  -3.79210 -4.26507 -2.87192
C  -3.33887 -4.35208 -1.54686
C  -2.85335 -3.21353 -0.88643
C  -3.01375  0.86090 -1.77037
C  -2.16026  1.34313 -2.78234
C  -2.64741  2.18580 -3.79416
C  -3.99865  2.56721 -3.79992
C  -4.85584  2.09384 -2.79384
C  -4.37076  1.23843 -1.79109
C   2.79405  1.17152 -2.15172
C   4.09004  1.65274 -2.43264
C   4.40571  2.15295 -3.70614
C   3.44000  2.15460 -4.72558
C   2.15921  1.64123 -4.46554
C   1.84308  1.15401 -3.18792
C  -2.02988  3.08123  0.39344
C  -2.31415  4.23515 -0.36491
C  -3.59548  4.80976 -0.36737
C  -4.61972  4.24572  0.40726
C  -4.36381  3.10060  1.17378
C  -3.08058  2.53190  1.15187
C  -0.03915  2.69723  2.35943
C  -0.62044  3.85104  2.93246
C  -0.40557  4.17060  4.28252
C   0.40296  3.34857  5.08392
C   0.99611  2.20893  4.52123
C   0.77333  1.88717  3.17212
C   2.01984 -2.79179  1.59443
C   2.87406 -3.83669  1.19635
C   3.90412 -4.27549  2.04620
C   4.08364 -3.68592  3.30638
C   3.22468 -2.65168  3.71740
C   2.19922 -2.20850  2.86807
H  -0.04238  0.00787  1.57018
H   0.00084 -1.16423 -1.63509
H   2.73718 -4.30062  0.21508
H   4.56656 -5.08345  1.71739
H   4.88276 -4.03367  3.96973
H   3.34762 -2.19422  4.70539
H   1.51447 -1.42094  3.20259
H  -1.66299 -1.33490  2.29278
H  -3.13982 -2.34060  4.03843
H  -2.94943 -4.78491  4.58234
H  -1.23335 -6.18059  3.40542
H   0.27406 -5.15586  1.73090
H  -0.01453 -5.26290 -0.26881
H  -0.50679 -6.19693 -2.51274
H  -0.45205 -4.67505 -4.51809
H   0.37609 -2.34346 -4.29596
H   3.40658 -3.81291 -2.87005
H   5.68914 -4.19142 -1.86341
H   6.60613 -2.54907 -0.21337
H   5.26056 -0.58035  0.48367
H   3.47839  3.21211 -0.43438
H   4.78624  4.46496  1.24734
H   5.49535  3.36329  3.38518
H   4.83438  0.98082  3.81952
H   3.49873 -0.26701  2.14877
H   4.86586  1.62617 -1.66162
H   5.41541  2.52927 -3.90345
H   3.69059  2.53699 -5.72106
H   1.40676  1.60903 -5.26146
H   0.86064  0.71903 -2.97810
H   0.17946  2.92950 -2.26476
H   1.31873  4.77928 -3.46698
H   2.41878  6.62844 -2.17363
H   2.37575  6.57297  0.33234
H  -1.25329  4.50435  2.32413
H  -0.87591  5.06432  4.70724
H   0.56783  3.59539  6.13837
H   1.63473  1.56172  5.13198
H   1.22383  0.99022  2.73981
H  -1.52328  4.69465 -0.96234
H  -3.78581  5.70287 -0.97102
H  -5.61834  4.69496  0.42074
H  -5.14771  2.64295  1.78380
O  -2.77024  1.41713  1.93581
H  -1.10939  1.03646 -2.76593
H  -1.96983  2.54482 -4.57656
H  -4.38188  3.22941 -4.58369
H  -5.91133  2.38669 -2.78908
H  -4.86886 -2.08166 -0.15709
H  -6.49473 -2.29929  1.68241
H  -6.28863 -0.85883  3.73354
H  -4.48401  0.91208  3.80458
H  -2.53138 -3.30004  0.15423
H  -3.36045 -5.31013 -1.01714
H  -4.17668 -5.15345 -3.38434
H  -4.08029 -2.94670 -4.57275
H  -3.24789 -0.93590 -3.40790
H   1.26494  4.70795  1.52485
H  -5.05402  0.86385 -1.02324
```

9cct (from IRC)

```
SCF  =           -3050.36443296
H(0 K)=          -3049.309476
G(298 K)=        -3049.416602
SCF(C6H6) =      -3050.37175381
BP86(D3BJ) =     -3050.89489127
Low Freq. = 12.4244cm-1, 17.0004cm-1
```

```
137
C  -2.69921 -2.47008 -3.07669
C  -1.85806 -1.62197 -2.32489
C  -0.88563 -0.79674 -2.97327
C  -0.75333 -0.92881 -4.39313
C  -1.55757 -1.81517 -5.11090
C  -2.54252 -2.59246 -4.46271
P  -2.06753 -1.25389 -0.52658
C  -3.84478 -0.57942 -0.66486
C  -4.95824 -1.08741  0.03279
C  -6.25008 -0.59282 -0.21891
C  -6.45174  0.42398 -1.16355
C  -5.35021  0.92958 -1.87478
C  -4.06475  0.42270 -1.63554
O  -0.13541  0.06674 -2.31310
C  -1.83018  2.90676 -1.85536
C  -2.19023  3.04027 -0.49752
C  -3.15547  4.01057 -0.14048
C  -3.73976  4.83031 -1.11977
C  -3.36848  4.69361 -2.46775
C  -2.41426  3.73068 -2.83217
P  -1.32239  1.87788  0.69773
C  -0.41394  3.07352  1.86140
C   0.52982  2.54760  2.76955
C   1.12836  3.36440  3.74268
C   0.81567  4.72999  3.81116
C  -0.09673  5.27267  2.89316
C  -0.70901  4.45277  1.93136
Ru -0.07311  0.03066 -0.07289
P   1.19388 -1.97355  0.42691
C   0.89394 -3.58894 -0.50552
C   0.83439 -3.51829 -1.91427
C   0.68337 -4.67958 -2.68936
C   0.58580 -5.93404 -2.06560
C   0.63721 -6.01512 -0.66498
C   0.78749 -4.85301  0.10991
P   1.93868  1.33476 -0.57410
C   3.09702  2.00573  0.76977
C   3.47531  1.16182  1.84114
C   4.24239  1.62406  2.92149
C   4.71306  2.94555  2.92527
C   4.41745  3.78433  1.84112
C   3.61881  3.31821  0.78409
C   1.59984  2.89198 -1.59098
C   1.70847  2.85749 -2.99804
C   1.42918  3.99851 -3.76751
C   1.04907  5.19943 -3.14920
C   0.93011  5.24369 -1.75214
C   1.18925  4.09880 -0.98233
C   3.22715  0.57200 -1.73492
C   2.81348 -0.33432 -2.73261
C   3.72663 -0.80691 -3.68962
```

```
C   5.06508 -0.38647 -3.66113
C   5.48429  0.51354 -2.66911
C   4.57313  0.99295 -1.71458
C  -2.44824 -2.85265  0.37088
C  -2.84738 -2.76430  1.72469
C  -3.21427 -3.91416  2.44081
C  -3.17818 -5.17274  1.81855
C  -2.76284 -5.27160  0.48232
C  -2.39764 -4.12179 -0.23802
C   3.08415 -2.00209  0.33135
C   3.82732 -2.94155 -0.41159
C   5.23150 -2.93403 -0.40123
C   5.92140 -1.98508  0.36682
C   5.20740 -1.04119  1.11809
C   3.80487 -1.05734  1.08822
C   1.08413 -2.54924  2.22876
C   2.04206 -3.44594  2.75513
C   1.97520 -3.86658  4.09276
C   0.95162 -3.39683  4.93143
C  -0.00267 -2.50567  4.41923
C   0.06497 -2.08733  3.07990
C  -2.69001  1.43954  1.94010
C  -4.06476  1.60309  1.67794
C  -5.02558  1.30041  2.65981
C  -4.63479  0.81636  3.91507
C  -3.26784  0.62962  4.18249
C  -2.30911  0.93760  3.20598
H   0.04518  0.03285  1.52062
H  -1.10363  2.13605 -2.14330
H  -4.40323  1.95433  0.70061
H  -6.08633  1.43637  2.42517
H  -5.38499  0.58537  4.67890
H  -2.94244  0.25575  5.15960
H  -1.25024  0.80386  3.44310
H   0.80142  1.48909  2.70171
H   1.85556  2.92892  4.43516
H   1.28622  5.36853  4.56639
H  -0.34002  6.34054  2.92341
H  -1.40968  4.90089  1.22279
H  -3.46922  4.11910  0.90233
H  -4.48985  5.57338 -0.82727
H  -3.82580  5.33374 -3.23023
H  -2.11985  3.61445 -3.88016
H  -0.01067 -0.30174 -4.89850
H  -1.43199 -1.89390 -6.19761
H  -3.18633 -3.26792 -5.03452
H  -3.49332 -3.03079 -2.57145
H  -4.83316 -1.88285  0.77108
H  -7.10091 -1.01236  0.32938
H  -7.45916  0.80829 -1.35705
H  -5.48702  1.71331 -2.62712
H  -3.22709  0.79768 -2.22785
H  -2.88957 -1.78647  2.21804
H  -3.52679 -3.82420  3.48658
H  -3.46554 -6.07098  2.37560
H  -2.71322 -6.24937 -0.00835
H  -2.05795 -4.21854 -1.27245
H   0.90229 -2.54875 -2.41193
H   0.62989 -4.59490 -3.77945
H   0.46626 -6.84158 -2.66728
H   0.55440 -6.98720 -0.16650
H   2.85737 -3.80884  2.12182
H   2.73185 -4.55780  4.47978
H   0.90315 -3.71952  5.97710
H  -0.80504 -2.12789  5.06238
H  -0.66471 -1.37570  2.68683
H   3.30037 -3.68885 -1.00990
H   5.78108 -3.67270 -0.99326
H   7.01621 -1.97539  0.38571
H   5.72782 -0.29087  1.71976
O   3.05016 -0.16506  1.85788
H   1.75579 -0.61096 -2.77142
H   3.38395 -1.50404 -4.46209
H   5.77711 -0.75524 -4.40747
H   6.52657  0.84920 -2.63469
H   3.39336  3.99344 -0.04473
H   4.80069  4.80944  1.81486
H   5.32020  3.30788  3.76132
H   4.46932  0.92960  3.73627
H   1.07349  4.15534  0.10193
H   0.62523  6.16899 -1.25184
H   0.84514  6.09176 -3.75055
H   1.52275  3.94551 -4.85772
H   2.01728  1.93813 -3.49970
H   0.81106 -4.93721  1.19927
H   4.91851  1.69975 -0.95478
```

5ctc

```
SCF  =           -3050.33158733
H(0 K)=          -3049.279744
G(298 K)=        -3049.388343
SCF(C6H6) =      -3050.33852248
BP86(D3BJ) =     -3050.84781805
Low Freq. = 10.0004cm-1, 19.3333cm-1
```

```
137
C  -2.91003 -2.35761 -0.84305
C  -2.06953 -2.08983 -1.94924
C  -2.64943 -2.19594 -3.23296
C  -4.00614 -2.51920 -3.40305
C  -4.82618 -2.73050 -2.28380
C  -4.27494 -2.64744 -0.99609
P  -0.29182 -1.49366 -1.65630
Ru  0.05943  0.25248 -0.00360
P  -0.04982 -1.18730  1.97327
C  -0.62411 -2.98719  1.84393
C  -1.72993 -3.30548  1.02495
C  -2.18145 -4.62519  0.86006
C  -1.52830 -5.66532  1.53450
C  -0.43817 -5.38153  2.37035
C   0.00260 -4.05681  2.51903
O  -2.40023 -2.23288  0.44777
P   2.25565  1.00485 -0.20400
C   2.50853  2.63800 -1.18773
C   1.54488  3.67360 -1.27787
C   1.81235  4.88839 -1.93712
C   3.06463  5.11752 -2.51775
C   4.03889  4.11083 -2.45437
C   3.75495  2.90115 -1.80488
O   0.24175  3.45354 -0.85668
C  -0.27957  3.93374  0.32547
C  -1.45020  3.26239  0.76673
C  -2.08845  3.76590  1.91618
C  -1.56969  4.86865  2.61965
C  -0.38748  5.48266  2.18277
C   0.26446  5.01929  1.02841
P  -1.92151  1.61967 -0.04913
C  -3.64016  1.38407  0.73001
C  -3.90646  0.27733  1.55424
C  -5.17802  0.09868  2.12893
C  -6.20007  1.02654  1.88432
C  -5.94697  2.13540  1.05744
C  -4.67980  2.31135  0.48327
C   3.20580  1.59506  1.33419
C   4.56669  1.97369  1.27108
C   5.22797  2.48049  2.39904
C   4.54061  2.62895  3.61519
C   3.19046  2.26066  3.69091
C   2.53330  1.74678  2.56010
C   3.57220 -0.02272 -1.08096
C   4.57572 -0.73215 -0.38430
C   5.45557 -1.58927 -1.06934
C   5.35069 -1.75210 -2.45963
C   4.35313 -1.05411 -3.16212
C   3.47084 -0.20341 -2.48091
C  -2.57570  2.19098 -1.72700
C  -2.48858  3.51735 -2.19583
C  -3.03622  3.87450 -3.44071
C  -3.68770  2.91590 -4.23146
C  -3.79525  1.59564 -3.76444
C  -3.24591  1.23847 -2.52410
H   0.10162  1.17448 -1.31176
H   0.27291  1.40637  1.10246
```

```
H   1.01164  5.63172 -1.99888
H   3.26840  6.06438 -3.02833
H   5.02047  4.25847 -2.91643
H   4.52461  2.12528 -1.79485
H  -2.99413  3.27337  2.28069
H  -2.08837  5.23787  3.51034
H   0.03199  6.33231  2.73192
H   1.18203  5.49391  0.66948
H  -3.11956 -0.45488  1.73675
H  -5.36116 -0.76860  2.77242
H  -7.19093  0.88859  2.33100
H  -6.73982  2.86425  0.85550
H  -4.49838  3.17473 -0.16520
H  -2.00470  4.28359 -1.58538
H  -2.95783  4.91138 -3.78640
H  -4.11520  3.19668 -5.20006
H  -4.30943  0.83626 -4.36307
H  -3.34906  0.20891 -2.17034
H   4.67559 -0.61242  0.69822
H   6.23361 -2.12173 -0.51046
H   6.03968 -2.41616 -2.99219
H   4.25308 -1.17450 -4.24573
H   2.69385  0.32468 -3.04270
H   1.48053  1.46194  2.62311
H   2.64007  2.36692  4.63156
H   5.05602  3.02903  4.49505
H   6.28322  2.76533  2.32367
H   5.12246  1.87748  0.33471
C   1.53855 -1.50108  2.95492
C  -1.13711 -0.61557  3.41031
C   0.56432 -3.17570 -1.55240
C   0.16406 -1.05231 -3.44080
H  -3.03822 -4.82374  0.20964
H  -1.87846 -6.69535  1.40884
H   0.07416 -6.18718  2.90581
H   0.85796 -3.84895  3.16710
H  -2.02928 -2.00408 -4.11328
H  -4.42219 -2.59355 -4.41326
H  -5.88948 -2.95969 -2.40787
H  -4.88539 -2.79567 -0.10063
C  -0.06778 -4.39223 -1.88454
C   0.64652 -5.60192 -1.85694
C   2.00635 -5.61368 -1.51037
C   2.64756 -4.40678 -1.18939
C   1.93030 -3.20030 -1.20610
H  -1.12310 -4.39886 -2.17212
H   0.13506 -6.53692 -2.11100
H   2.56274 -6.55710 -1.49265
H   3.71094 -4.39536 -0.92858
H   2.44040 -2.26264 -0.97026
C  -0.06304  0.26495 -3.89476
C   0.26023  0.63863 -5.20916
C   0.82526 -0.29628 -6.09218
C   1.05337 -1.60985 -5.65293
C   0.72178 -1.98686 -4.33992
H  -0.48734  1.00465 -3.20940
H   0.07234  1.66621 -5.53779
H   1.08569 -0.00294 -7.11499
H   1.49151 -2.34966 -6.33219
H   0.90603 -3.01488 -4.01547
C  -1.22576  0.76674  3.67596
C  -1.96598  1.24356  4.76914
C  -2.64875  0.34528  5.60488
C  -2.57917 -1.03236  5.34251
C  -1.82190 -1.51069  4.25957
H  -0.73132  1.47590  3.00366
H  -2.01819  2.32175  4.95412
H  -3.23621  0.71628  6.45162
H  -3.11018 -1.74233  5.98642
H  -1.76198 -2.58825  4.07751
C   1.66248 -1.27382  4.34073
C   2.87080 -1.54483  5.00692
C   3.96690 -2.06593  4.30429
C   3.85016 -2.30577  2.92486
C   2.65177 -2.01367  2.25628
H   0.81370 -0.88529  4.91011
H   2.94814 -1.35265  6.08278
H   4.90507 -2.28402  4.82567
H   4.69555 -2.72025  2.36457
H   2.57002 -2.19919  1.18133
```

5ctc (from IRC)

```
SCF  =           -3050.32151335
H(0 K)=          -3049.269257
G(298 K)=        -3049.375825
SCF(C6H6) =      -3050.32849114
BP86(D3BJ) =     -3050.84033967
Low Freq. = 17.4315cm-1, 21.2633cm-1
```

```
137
C   1.61804  2.58378  2.32761
C   0.99622  1.50398  2.99603
C   0.32064  1.79109  4.19850
C   0.28054  3.09591  4.71746
C   0.92208  4.14202  4.03712
C   1.59962  3.89002  2.83478
P   1.00586 -0.14930  2.10391
Ru -0.08767 -0.28433 -0.06755
P  -0.18608  2.09740 -0.64824
C   1.42412  3.06171 -0.93460
C   2.42227  3.08045  0.06945
C   3.61647  3.80163 -0.07862
C   3.84856  4.53114 -1.25446
C   2.88878  4.51405 -2.27648
C   1.69490  3.79027 -2.11300
O   2.30735  2.23440  1.17294
P   1.51514 -1.20316 -1.55734
C   1.62826 -3.07650 -1.27931
C   0.46022 -3.85305 -1.03911
C   0.55648 -5.15724 -0.52168
C   1.81420 -5.74354 -0.31514
C   2.97841 -5.03138 -0.62450
C   2.87398 -3.70862 -1.08387
O  -0.75558 -3.25096 -1.36755
C  -1.94746 -3.65215 -0.76712
C  -2.69067 -2.74994  0.04705
C  -3.86563 -3.27437  0.63570
C  -4.32480 -4.57735  0.39639
C  -3.63425 -5.40169 -0.50132
C  -2.45146 -4.93121 -1.08347
P  -2.33165 -0.86650  0.18336
C  -3.47661 -0.30136 -1.21210
C  -3.94559  1.03085 -1.20900
C  -4.79957  1.49424 -2.22033
C  -5.18825  0.63977 -3.26478
C  -4.71390 -0.67973 -3.28794
C  -3.86818 -1.14855 -2.26760
C   1.12276 -1.10780 -3.40170
C   1.52936  0.03805 -4.12108
C   1.27613  0.15010 -5.49652
C   0.60663 -0.87955 -6.17847
C   0.19220 -2.01865 -5.47132
C   0.44977 -2.13405 -4.09409
C   3.34803 -0.76240 -1.73080
C   4.14143 -1.25245 -2.79565
C   5.49147 -0.88944 -2.90497
C   6.06827 -0.02481 -1.95752
C   5.28657  0.47270 -0.90578
C   3.93309  0.10682 -0.79517
C  -3.48980 -0.42316  1.63855
C  -4.89478 -0.31436  1.50646
C  -5.69613  0.04602  2.60191
C  -5.11131  0.30483  3.85248
C  -3.71846  0.20917  3.99535
C  -2.91875 -0.14461  2.89541
H  -0.01035 -1.81814  0.37682
H  -0.78345 -0.56213 -1.49642
H  -0.34694 -5.71873 -0.27868
H   1.87069 -6.76282  0.08130
H   3.96512 -5.48518 -0.48725
H   3.78856 -3.13654 -1.25598
H  -4.45336 -2.63274  1.29571
H  -5.23880 -4.92797  0.88648
```

```
H  -4.00699 -6.40043 -0.75106
H  -1.90064 -5.54441 -1.80218
H  -3.65287  1.71296 -0.40446
H  -5.15035  2.53096 -2.19554
H  -5.85384  1.00197 -4.05550
H  -5.00822 -1.35765 -4.09679
H  -3.52493 -2.18630 -2.29687
H  -5.36827 -0.50400  0.53865
H  -6.78133  0.12635  2.47434
H  -5.73691  0.58661  4.70630
H  -3.24744  0.41601  4.96222
H  -1.83303 -0.19034  3.00962
H   3.70012 -1.91736 -3.54526
H   6.09297 -1.27833 -3.73412
H   7.12230  0.26081 -2.04535
H   5.72371  1.14816 -0.16360
H   3.32527  0.49595  0.02399
H   0.12191 -3.02940 -3.56108
H  -0.32669 -2.83096 -5.99229
H   0.41341 -0.79560 -7.25335
H   1.60334  1.04598 -6.03496
H   2.05767  0.84599 -3.60404
C  -1.11466  2.57676 -2.23648
C  -0.98842  3.35320  0.52629
C   2.82457 -0.64925  2.31269
C   0.44450 -1.34248  3.47167
H   4.35763  3.76138  0.72550
H   4.77957  5.09482 -1.37302
H   3.06216  5.06749 -3.20531
H   0.95099  3.79630 -2.91485
H  -0.18280  0.98284  4.73693
H  -0.25306  3.29247  5.65285
H   0.89511  5.16069  4.43723
H   2.09813  4.69259  2.28359
C   3.79782  0.14501  2.94958
C   5.10962 -0.33245  3.11799
C   5.46227 -1.61306  2.66575
C   4.49259 -2.41647  2.04356
C   3.18656 -1.93742  1.86722
H   3.53669  1.13806  3.32707
H   5.85457  0.30010  3.61409
H   6.48377 -1.98436  2.80118
H   4.74910 -3.42071  1.68978
H   2.44035 -2.57332  1.38009
C  -0.43652 -2.39886  3.17283
C  -0.77905 -3.35244  4.14743
C  -0.24749 -3.25982  5.44163
C   0.64524 -2.21885  5.75034
C   0.99808 -1.27842  4.77147
H  -0.84669 -2.47962  2.16137
H  -1.46459 -4.16624  3.88797
H  -0.51606 -3.99910  6.20398
H   1.08083 -2.14643  6.75301
H   1.72724 -0.49907  5.01534
C  -0.70364  4.73744  0.49578
C  -1.41732  5.63413  1.30686
C  -2.41811  5.16329  2.17304
C  -2.68737  3.78788  2.23412
C  -1.97337  2.89407  1.41839
H   0.08336  5.11986 -0.16258
H  -1.18671  6.70456  1.26311
H  -2.97399  5.86436  2.80513
H  -3.44705  3.39879  2.92037
H  -2.16668  1.82046  1.48453
C  -1.66399  3.85882 -2.46624
C  -2.31023  4.15985 -3.67564
C  -2.41986  3.18651 -4.68206
C  -1.89080  1.90699 -4.46116
C  -1.25262  1.60567 -3.24693
H  -1.60013  4.63189 -1.69613
H  -2.72956  5.16079 -3.82798
H  -2.92297  3.42288 -5.62620
H  -1.97565  1.13012 -5.22726
H  -0.87050  0.59752 -3.07177
```

TS(5-9)ctc

```
SCF  =           -3050.28281141
H(0 K)=          -3049.233218
G(298 K)=        -3049.338271
SCF(C6H6) =      -3050.28988954
BP86(D3BJ) =     -3050.80803949
Low Freq. = -233.4547cm-1, 20.6206cm-1

137
C   1.33714 -0.84879  3.36309
C   0.64710 -1.92136  2.75258
C  -0.12230 -2.75712  3.58471
C  -0.19508 -2.53259  4.96987
C   0.50989 -1.46496  5.54632
C   1.28750 -0.61613  4.74376
P   0.75013 -2.01287  0.88142
Ru -0.09413 -0.03589 -0.36138
P  -0.18827  1.60411  1.35928
C   1.46746  2.19428  2.06326
C   2.35755  1.26362  2.65008
C   3.56523  1.66148  3.24194
C   3.91839  3.01929  3.25765
C   3.06699  3.96182  2.66342
C   1.85718  3.55076  2.07752
O   2.12396 -0.10179  2.49560
P   1.84179  0.77506 -1.61700
C   1.79745 -0.48969 -2.96763
C   0.53600 -1.22585 -2.99770
C   0.59559 -2.53819 -3.59446
C   1.75070 -2.99417 -4.23762
C   2.90501 -2.19595 -4.33565
C   2.91599 -0.95720 -3.66240
O  -0.65364 -0.38902 -3.49870
C  -1.84438 -1.07365 -3.54898
C  -2.70322 -1.23468 -2.42007
C  -3.87312 -2.01027 -2.60197
C  -4.21638 -2.56757 -3.84178
C  -3.38817 -2.35730 -4.95514
C  -2.20737 -1.62474 -4.79976
P  -2.38987 -0.34359 -0.77384
C  -3.36136  1.23872 -1.09561
C  -3.86789  1.98019 -0.00652
C  -4.60334  3.15551 -0.22106
C  -4.83240  3.61709 -1.52712
C  -4.31815  2.89600 -2.61512
C  -3.59011  1.71330 -2.40227
C   1.64193  2.43574 -2.47291
C   2.16076  3.61630 -1.89853
C   1.99208  4.85426 -2.53883
C   1.29920  4.92980 -3.75863
C   0.77229  3.76006 -4.33070
C   0.94128  2.51908 -3.69357
C   3.67634  0.89800 -1.20537
C   4.57879  1.55803 -2.07297
C   5.95308  1.58335 -1.79020
C   6.44793  0.95494 -0.63512
C   5.55962  0.30274  0.23220
C   4.18327  0.27544 -0.05038
C  -3.66486 -1.22955  0.32707
C  -5.05052 -0.95195  0.26324
C  -5.95945 -1.63642  1.08621
C  -5.50269 -2.61209  1.98758
C  -4.12986 -2.89341  2.06378
C  -3.22197 -2.20364  1.24276
H   0.13280 -1.21731 -1.74207
H  -0.59258  1.13872 -1.32782
H  -0.29880 -3.16716 -3.58334
H   1.73565 -3.99164 -4.69310
H   3.79745 -2.55439 -4.85668
H   3.85207 -0.39209 -3.59701
H  -4.54657 -2.16889 -1.75662
H  -5.13889 -3.15010 -3.93522
H  -3.65205 -2.77276 -5.93369
H  -1.51929 -1.46298 -5.63453
H  -3.70109  1.63449  1.01872
H  -4.98865  3.71451  0.63768
H  -5.40717  4.53420 -1.69454
H  -4.48980  3.24554 -3.63906
```

```
H  -3.20884  1.15524 -3.26181
H  -5.42349 -0.19418 -0.43181
H  -7.02807 -1.40430  1.02093
H  -6.21242 -3.14505  2.62939
H  -3.75896 -3.64724  2.76618
H  -2.15252 -2.41203  1.32566
H   4.20678  2.06051 -2.97107
H   6.63727  2.09667 -2.47490
H   7.52080  0.97585 -0.41442
H   5.93254 -0.19627  1.13255
H   3.50227 -0.24313  0.62629
H   0.52372  1.60947 -4.13744
H   0.23300  3.80900 -5.28317
H   1.17368  5.89483 -4.26162
H   2.40401  5.76113 -2.08281
H   2.70888  3.56777 -0.95165
C  -0.95088  3.28706  0.91263
C  -1.05659  1.33859  3.02427
C   2.52826 -2.62857  0.66685
C  -0.00286 -3.71195  0.51912
H   4.22441  0.89443  3.65911
H   4.86159  3.33223  3.71674
H   3.33765  5.02271  2.65647
H   1.19561  4.29777  1.63001
H  -0.66935 -3.59603  3.14410
H  -0.80159 -3.19415  5.59655
H   0.45810 -1.28720  6.62528
H   1.84097  0.22371  5.17356
C   3.40467 -2.88728  1.73985
C   4.69112 -3.40171  1.50126
C   5.11435 -3.67188  0.19052
C   4.24085 -3.42868 -0.88161
C   2.95818 -2.91198 -0.64652
H   3.08637 -2.69753  2.76936
H   5.35930 -3.59815  2.34753
H   6.11686 -4.07288  0.00640
H   4.55093 -3.63248 -1.91157
H   2.29173 -2.73751 -1.49707
C  -0.93241 -3.86138 -0.52643
C  -1.43816 -5.12753 -0.86914
C  -1.02046 -6.26537 -0.16468
C  -0.08137 -6.13296  0.87340
C   0.43251 -4.87057  1.20333
H  -1.26240 -2.97891 -1.08226
H  -2.16017 -5.21497 -1.68779
H  -1.41443 -7.25309 -0.42719
H   0.26309 -7.01778  1.41983
H   1.19270 -4.78744  1.98683
C  -0.69563  2.04550  4.19470
C  -1.44236  1.90525  5.37517
C  -2.55556  1.04929  5.41277
C  -2.90952  0.32778  4.26333
C  -2.16277  0.47382  3.08216
H   0.17120  2.71365  4.18644
H  -1.14988  2.46729  6.26918
H  -3.13614  0.94044  6.33520
H  -3.76294 -0.35830  4.27646
H  -2.43569 -0.10449  2.19800
C  -1.52066  4.14978  1.87661
C  -2.02437  5.40746  1.50867
C  -1.96819  5.83003  0.17107
C  -1.41566  4.97801 -0.79600
C  -0.91823  3.71737 -0.42849
H  -1.58173  3.84383  2.92391
H  -2.46271  6.05654  2.27502
H  -2.36004  6.81231 -0.11484
H  -1.36975  5.28276 -1.84591
H  -0.51293  3.05431 -1.19570
```

9ctc

```
SCF  =          -3050.37280812
H(0 K)=         -3049.317995
G(298 K)=       -3049.427141
SCF(C6H6) =     -3050.38010609
BP86(D3BJ) =    -3050.89111623
Low Freq. = 14.5528cm-1, 16.3801cm-1
```

```
137
C  -0.14532 -2.24328  2.99876
C  -0.16315 -2.98288  1.79347
C  -0.91084 -4.17285  1.75474
C  -1.66254 -4.58349  2.87188
C  -1.66878 -3.80094  4.03723
C  -0.90497 -2.62325  4.11417
P   0.84973 -2.23188  0.40175
Ru -0.02046  0.08021 -0.42175
P  -0.56627  1.26219  1.56066
C   0.17789  1.13958  3.33320
C   0.64393 -0.09378  3.83392
C   1.13083 -0.24550  5.14404
C   1.10896  0.84910  6.01809
C   0.60391  2.07928  5.56856
C   0.14791  2.21594  4.24871
O   0.71817 -1.16297  2.95253
P   2.05743  1.13441 -0.99169
C   3.49792  0.16223 -1.76177
C   3.28015 -1.08798 -2.38173
C   4.34976 -1.79022 -2.96142
C   5.64928 -1.26101 -2.93371
C   5.87432 -0.01758 -2.32236
C   4.80865  0.68943 -1.74199
O   0.39754 -0.63536 -2.38227
C  -0.55270 -0.76813 -3.29361
C  -1.93467 -0.64543 -2.97460
C  -2.91725 -0.84159 -3.96943
C  -2.55432 -1.14564 -5.28539
C  -1.18387 -1.26030 -5.61069
C  -0.19814 -1.07798 -4.64241
P  -2.27857 -0.22529 -1.20682
C  -3.29155  1.35301 -1.36840
C  -4.32764  1.68273 -0.46848
C  -5.04186  2.88533 -0.61278
C  -4.72935  3.77476 -1.65326
C  -3.69393  3.45632 -2.54872
C  -2.98072  2.25579 -2.40762
C   1.77710  2.38936 -2.36114
C   1.04442  3.56478 -2.08631
C   0.79602  4.50206 -3.09989
C   1.25528  4.27080 -4.40773
C   1.97079  3.09841 -4.69109
C   2.23736  2.16578 -3.67362
C   3.02139  2.12499  0.27952
C   3.62271  3.37167  0.00558
C   4.37337  4.03321  0.99230
C   4.53867  3.45668  2.26245
C   3.94471  2.21487  2.54410
C   3.18646  1.55821  1.56136
C  -3.67235 -1.44662 -0.80450
C  -4.99157 -1.28324 -1.28540
C  -5.96985 -2.26135 -1.04462
C  -5.65309 -3.41727 -0.31293
C  -4.34883 -3.58673  0.17672
C  -3.37186 -2.60898 -0.07026
H   2.26373 -1.48861 -2.42066
H  -0.50005  1.44929 -1.07679
H   4.15868 -2.76060 -3.43196
H   6.48180 -1.81324 -3.38313
H   6.88321  0.40870 -2.29319
H   5.00849  1.65324 -1.26505
H  -3.97731 -0.75540 -3.70917
H  -3.32287 -1.28946 -6.05123
H  -0.88757 -1.49747 -6.63936
H   0.86457 -1.17134 -4.88723
H  -4.58483  1.00135  0.34777
H  -5.84326  3.12306  0.09488
H  -5.29217  4.70748 -1.76897
H  -3.44231  4.14037 -3.36630
H  -2.17925  2.01206 -3.11189
H  -5.26691 -0.38098 -1.83901
H  -6.98541 -2.11459 -1.42863
H  -6.41920 -4.17641 -0.12184
H  -4.08535 -4.47506  0.76066
H  -2.36341 -2.74752  0.32806
```

```
H   3.49670  3.83236 -0.97893
H   4.83128  5.00210  0.76451
H   5.12459  3.97395  3.02982
H   4.05730  1.75918  3.53324
H   2.70781  0.60236  1.79418
H   2.80081  1.25894 -3.90767
H   2.32781  2.90270 -5.70794
H   1.05395  4.99952 -5.20039
H   0.23519  5.41214 -2.86198
H   0.66455  3.75194 -1.07826
C  -0.47456  3.13425  1.31005
C  -2.32795  1.08591  2.24284
C   2.53843 -2.62981  1.19074
C   0.77877 -3.60251 -0.89008
H   1.52507 -1.21823  5.45302
H   1.48174  0.73940  7.04163
H   0.56497  2.93982  6.24443
H  -0.24346  3.18396  3.92634
H  -0.90828 -4.77598  0.84048
H  -2.23705 -5.51448  2.83035
H  -2.26242 -4.10953  4.90407
H  -0.89686 -2.01522  5.02261
C   2.69133 -3.76058  2.02706
C   3.93297 -4.07045  2.60269
C   5.05152 -3.25712  2.35602
C   4.91451 -2.13746  1.52411
C   3.66928 -1.83099  0.94799
H   1.83367 -4.40796  2.23262
H   4.02372 -4.95259  3.24623
H   6.02015 -3.49761  2.80745
H   5.77444 -1.49303  1.31367
H   3.59275 -0.96526  0.29150
C  -0.34915 -3.67392 -1.73523
C  -0.45540 -4.67119 -2.71718
C   0.57741 -5.60639 -2.88654
C   1.71498 -5.53360 -2.06770
C   1.81647 -4.54211 -1.07753
H  -1.14832 -2.93465 -1.64421
H  -1.34111 -4.69879 -3.35988
H   0.50073 -6.38024 -3.65797
H   2.53237 -6.25204 -2.19514
H   2.71142 -4.50283 -0.45152
C  -3.04246  2.14328  2.84588
C  -4.30600  1.91729  3.41341
C  -4.86989  0.62978  3.40362
C  -4.15674 -0.43221  2.82701
C  -2.89491 -0.20168  2.25304
H  -2.62118  3.15234  2.86479
H  -4.85177  2.75302  3.86531
H  -5.85737  0.45735  3.84512
H  -4.57928 -1.44181  2.80642
H  -2.34388 -1.03034  1.80233
C  -1.51258  3.77607  0.59706
C  -1.45591  5.15127  0.32034
C  -0.35479  5.91525  0.74032
C   0.69100  5.28681  1.43276
C   0.63400  3.91030  1.71354
H  -2.37711  3.20329  0.25340
H  -2.28051  5.61760 -0.22850
H  -0.31089  6.98874  0.52710
H   1.56458  5.86164  1.75728
H   1.46521  3.44797  2.25113
```

4. Small Model of cis-Ru(DPEphos)$_2$H$_2$

5ccc

```
SCF  =           -1201.99987247
H(0 K)=          -1201.588220
G(298 K)=        -1201.649536
SCF(C6H6) =      -1202.00529593
BP86(D3BJ) =     -1202.18062579
Low Freq. = 19.6051cm-1, 22.9291cm-1
```

57

```
C   2.57760 -1.78376 -0.05606
C   1.76961 -1.56215  1.08260
C   1.41339 -2.68664  1.85486
C   1.83835 -3.97864  1.50485
C   2.63067 -4.17103  0.36261
C   3.00182 -3.06890 -0.42351
P   1.11871  0.15545  1.46923
H   0.49440 -0.21624  2.71930
Ru  0.00004  1.60321  0.00007
P  -1.80744  1.97906  1.37133
H  -1.85875  1.40385  2.69773
P   1.80741  1.97899 -1.37132
H   2.07509  3.33193 -1.77381
H   1.85853  1.40370 -2.69770
C   3.52263  1.57034 -0.77874
C   3.82843  0.22510 -0.48697
C   5.03425 -0.13916  0.13145
C   5.96357  0.86263  0.45340
C   5.69149  2.20770  0.15392
C   4.47528  2.55543 -0.45675
P  -1.11845  0.15530 -1.46909
H  -0.49386 -0.21649 -2.71899
C  -1.76953 -1.56225 -1.08249
C  -1.41337 -2.68679 -1.85469
C  -1.83859 -3.97872 -1.50472
C  -2.63109 -4.17097 -0.36259
C  -3.00216 -3.06878  0.42349
C  -2.57769 -1.78371  0.05607
H  -2.32399  0.66587 -2.07708
H   2.32439  0.66607  2.07689
H  -2.07509  3.33203  1.77372
C  -3.52262  1.57046  0.77860
C  -3.82844  0.22522  0.48684
C  -5.03423 -0.13901 -0.13164
C  -5.96351  0.86279 -0.45365
C  -5.69142  2.20786 -0.15417
C  -4.47523  2.55557  0.45656
H  -0.65942  2.85080 -0.82618
H   0.65944  2.85084  0.82632
H   5.23618 -1.19231  0.34896
H   6.90695  0.58603  0.93548
H   6.42013  2.98585  0.40174
H   4.25239  3.60683 -0.67023
H  -0.78444 -2.54659 -2.74095
H  -1.54559 -4.83347 -2.12277
H  -2.96216 -5.17566 -0.08059
H  -3.61320 -3.18536  1.32376
O  -2.89210 -0.71945  0.90797
H  -5.23618 -1.19216 -0.34915
H  -6.90688  0.58620 -0.93576
H  -4.25232  3.60697  0.67002
H   0.78461 -2.54634  2.74121
H   1.54531 -4.83334  2.12295
H   2.96155 -5.17577  0.08058
H   3.61274 -3.18558 -1.32385
O   2.89208 -0.71959 -0.90805
H  -6.42003  2.98603 -0.40203
```

TS(5-9)ccc

```
SCF  =           -1201.93459417
H(0 K)=          -1201.525703
G(298 K)=        -1201.586308
SCF(C6H6) =      -1201.94040354
BP86(D3BJ) =     -1202.11669541
Low Freq. = -234.6537cm-1, 19.3151cm-1
```

57

```
C   3.77640 -0.77900 -0.12823
C   3.11997 -2.00692 -0.35025
C   3.64918 -3.16539  0.25089
C   4.79585 -3.09378  1.05853
C   5.42311 -1.85570  1.27673
C   4.91859 -0.68872  0.68079
P   1.49070 -1.98119 -1.24197
H   1.94500 -1.61386 -2.56392
Ru -0.29766 -0.96070 -0.26938
P  -1.96635 -2.38898 -1.11281
H  -2.44865 -2.37142 -2.47769
```

```
P   0.96647  0.19683  1.27251
H   1.86767 -0.57690  2.08347
H   0.26340  0.82903  2.35651
C   2.09018  1.63884  0.87870
C   3.09206  1.52540 -0.11299
C   3.87207  2.62478 -0.49961
C   3.66829  3.86897  0.11771
C   2.68862  4.00536  1.11359
C   1.90714  2.89889  1.48440
P  -0.66518  0.82151 -1.77750
H  -1.31909  0.61345 -3.04803
C  -1.72853  2.13917 -1.03926
C  -1.34324  3.48867 -0.91596
C  -2.17940  4.40592 -0.26064
C  -3.40704  3.97577  0.27572
C  -3.80268  2.63586  0.16265
C  -2.96660  1.70332 -0.49371
H   0.39325  1.66063 -2.28565
H   1.40090 -3.39979 -1.49339
H  -1.80860 -3.81516 -1.01120
C  -3.36218 -1.98333 -0.01065
C  -3.17098 -0.63934  0.51857
C  -3.94159 -0.31025  1.68761
C  -4.89271 -1.20496  2.18993
C  -5.13815 -2.45464  1.58820
C  -4.34343 -2.83843  0.48810
H  -1.91969 -0.46706  0.63677
H  -0.25391 -2.11608  0.89587
H   4.62364  2.48870 -1.28297
H   4.27672  4.72852 -0.18102
H   2.52594  4.97273  1.59896
H   1.13153  3.01639  2.24916
H  -0.39119  3.82417 -1.34209
H  -1.88154  5.45665 -0.18210
H  -4.06682  4.69433  0.77420
H  -4.76638  2.29214  0.54861
O  -3.34544  0.40639 -0.68355
H  -3.79641  0.66424  2.16443
H  -5.47262 -0.90829  3.07225
H  -4.44345 -3.84633  0.06677
H   3.14860 -4.12765  0.09670
H   5.19435 -4.00192  1.52121
H   6.31434 -1.79453  1.90952
H   5.40148  0.28140  0.83232
O   3.26157  0.32626 -0.81084
H  -5.88452 -3.13908  2.00065
```

5cct

```
SCF  =           -1201.98085177
H(0 K)=          -1201.569827
G(298 K)=        -1201.631331
SCF(C6H6) =      -1201.98812535
BP86(D3BJ) =     -1202.15785058
Low Freq. = 16.0201cm-1, 17.8416cm-1
```

```
57
C   3.38607 -2.66706 -1.96155
C   2.99180 -2.00388 -0.78432
C   3.94838 -1.22464 -0.10239
C   5.26082 -1.08450 -0.57909
C   5.62890 -1.74736 -1.76032
C   4.69779 -2.54353 -2.44843
P   1.23271 -1.94153 -0.18147
H   0.76927 -3.12616 -0.87175
O   3.53266 -0.65924  1.10369
C   3.81129  0.68761  1.34922
C   3.16129  1.69776  0.60424
C   3.42229  3.03797  0.95332
C   4.28763  3.36157  2.01128
C   4.90677  2.33854  2.74592
C   4.66985  0.99532  2.41397
P   1.89697  1.25751 -0.70580
Ru  0.00000 -0.00001 -0.37472
P  -1.89696 -1.25755 -0.70580
C  -3.16128 -1.69777  0.60425
C  -3.42231 -3.03798  0.95333
C  -4.28767 -3.36155  2.01127
C  -4.90683 -2.33852  2.74588
C  -4.66988 -0.99530  2.41393
C  -3.81129 -0.68761  1.34920
O  -3.53264  0.65924  1.10366
C  -3.94836  1.22466 -0.10240
C  -2.99179  2.00389 -0.78433
C  -3.38606  2.66709 -1.96155
C  -4.69779  2.54358 -2.44841
C  -5.62889  1.74741 -1.76031
C  -5.26082  1.08453 -0.57908
P  -1.23269  1.94151 -0.18149
H  -0.76925  3.12614 -0.87177
H  -1.40139  2.56570  1.10355
H   1.40143 -2.56571  1.10357
H  -2.79978 -0.81523 -1.73404
H   2.79978  0.81517 -1.73403
H  -1.72271 -2.60116 -1.20122
H   1.72272  2.60112 -1.20124
H   0.00009 -0.00005 -2.07616
H  -0.00007 -0.00002  1.29859
H   2.92996  3.84126  0.39294
H   4.47084  4.41086  2.26363
H   5.57591  2.58183  3.57746
H   5.13272  0.17586  2.97187
H   5.97534 -0.46756 -0.02575
H   6.65094 -1.64457 -2.13936
H   4.98987 -3.06305 -3.36640
H   2.65457 -3.27128 -2.50934
H  -2.92996 -3.84127  0.39297
H  -4.47090 -4.41084  2.26363
H  -5.57599 -2.58179  3.57741
H  -5.13276 -0.17583  2.97182
H  -2.65456  3.27131 -2.50934
H  -4.98987  3.06311 -3.36638
H  -6.65095  1.64464 -2.13933
H  -5.97534  0.46759 -0.02574
```

TS(5-9)cct

```
SCF  =           -1201.92582482
H(0 K)=          -1201.517891
G(298 K)=        -1201.578637
SCF(C6H6) =      -1201.93188774
BP86(D3BJ) =     -1202.10867360
Low Freq. = -753.6418cm-1, 17.3887cm-1
```

```
57
C  -4.55794  0.42379 -2.34125
C  -3.43580  0.69597 -1.53489
C  -3.60969  0.93261 -0.14932
C  -4.89232  0.85841  0.42764
C  -5.99992  0.58248 -0.38755
C  -5.83925  0.37471 -1.76980
P  -1.68174  0.59014 -2.11426
H  -1.94215  0.23731 -3.48789
O  -2.50029  1.30724  0.57016
C  -1.91468  0.32246  1.54009
C  -2.37714 -1.06141  1.56020
C  -2.61623 -1.72484  2.77088
C  -2.43097 -1.08187  4.00843
C  -1.95842  0.24674  4.00700
C  -1.72905  0.94542  2.81942
P  -1.96542 -1.91688 -0.00644
Ru -0.17408 -0.65216 -0.85353
P   1.53226  0.71070 -1.57914
C   2.36032  1.97347 -0.47442
C   2.32075  3.34855 -0.78123
C   2.84876  4.30689  0.09909
C   3.42435  3.90023  1.31266
C   3.48243  2.53622  1.63883
C   2.96036  1.58969  0.74632
O   2.95770  0.25039  1.14264
C   3.73321 -0.66358  0.43012
C   3.10201 -1.87747  0.09104
C   3.86281 -2.87270 -0.55082
C   5.21603 -2.65226 -0.85598
```

C	5.81906	-1.42744	-0.52382
C	5.08196	-0.42468	0.12537
P	1.26774	-2.00011	0.34868
H	1.14952	-3.42623	0.17321
H	1.21016	-2.01302	1.79026
H	-1.37323	1.97839	-2.36673
H	2.70947	0.11200	-2.15633
H	-3.17355	-2.10845	-0.76921
H	1.26815	1.62117	-2.66674
H	-1.81530	-3.29101	0.40906
H	0.12632	-1.60429	-2.15364
H	-0.69002	0.21841	0.70594
H	-2.89418	-2.78620	2.74579
H	-2.60986	-1.61541	4.94602
H	-1.80053	0.76630	4.95970
H	-1.42439	1.99638	2.83935
H	-5.00120	1.03466	1.50134
H	-6.99940	0.53663	0.05798
H	-6.71036	0.16910	-2.40010
H	-4.42811	0.24131	-3.41405
H	1.85790	3.67393	-1.71976
H	2.80214	5.36892	-0.16139
H	3.82985	4.64175	2.00831
H	3.91835	2.18869	2.58012
H	3.38688	-3.81971	-0.82769
H	5.79588	-3.43317	-1.35754
H	6.87239	-1.25048	-0.76433
H	5.53905	0.53071	0.40006

References

1 M. K. Cybulski, J. E. Nicholls, J. P. Lowe, M. F. Mahon, M. K. Whittlesey, *Organometallics* **2017**, *36*, 2308-2316.

2 R. Young, G. Wilkinson, *Inorg. Synth.* **1990**, *28*, 337-338.

3 G. M. Sheldrick, *Acta Cryst.* **2015**, *C71*, 3–8.

4 O. V. Dolomanov, L. J. Bourhis, R. J. Gildea, J. A. K. Howard, H. Puschmann, *J. Appl. Cryst.* **2009**, *42*, 339–341.

5 Gaussian 09, Revision D.01: M. J. Frisch, G. W. Trucks, H. B. Schlegel, G. E. Scuseria, M. A Robb, J. R. Cheeseman, G. Scalmani, V. Barone, B. Mennucci, G. A. Petersson, H. Nakatsuji, M. Caricato, X. Li, H. P. Hratchian, A. F. Izmaylov, J. Bloino, G. Zheng, J. L. Sonnenberg, M. Hada, M. Ehara, K. Toyota, R. Fukuda, J. Hasegawa, M. Ishida, T. Nakajima, Y. Honda, O. Kitao, H. Nakai, T. Vreven, J. A. Montgomery, J. E. Peralta, F. Ogliaro, M. Bearpark, J. J. Heyd, E. Brothers, K. N. Kudin, V. N. Staroverov, R. Kobayashi, J. Normand, K. Raghavachari, A. Rendell, J. C. Burant, S. S. Iyengar, J. Tomasi, M. Cossi, N. Rega, J. M. Millam, M. Klene, J. E. Knox, J. B. Cross, V. Bakken, C. Adamo, J. Jaramillo, R. Gomperts, R. E. Stratmann, O. Yazyev, A. J. Austin, R. Cammi, C. Pomelli, J. W. Ochterski, R. L. Martin, K. Morokuma, V. G. Zakrzewski, G. A. Voth, P. Salvador, J. J. Dannenberg, S. Dapprich, A. D. Daniels, O. Farkas, J. B. Foresman, J. V. Ortiz, J. Cioslowski, D. J. Fox, Wallingford CT, 2013.

6 D. Andrae, U. Häußermann, M. Dolg, H. Stoll, H. Preuß, *Theor. Chim. Acta.* **1990**, *77*, 123-141.

7 a) W. J. Hehre, R. Ditchfield, J. A. Pople, *J. Chem. Phys*. **1972**, *56*, 2257-2261; b) P. C. Hariharan, J. A. Pople, *Theor. Chim. Acta.* **1973**, *28*, 213-222.

8 A. Hollwarth, M. Bohme, S. Dapprich, A. W. Ehlers, A. Gobbi, V. Jonas, K. F. Kohler, R. Stegmann, A. Veldkamp, G. Frenking, *Chem. Phys. Lett.* **1993**, *208*, 237-240.

9 a) A. D. Becke, *Phys. Rev. A* **1988**, *38*, 3098-3100; b) J. P. Perdew, *Phys. Rev. B* **1986**, *33*, 8822-8824.

10 J. Tomasi, B. Mennucci, R. Cammi, *Chem. Rev.* **2005**, *105*, 2999-3094.

11 S. Grimme, S. Ehrlich, L. Goerigk, *J. Comput. Chem.* **2011**, *32*, 1456-1465.

12 NBO 6.0.: E. D. Glendening, J. K. Badenhoop, A. E. Reed, J. E. Carpenter, J. A. Bohmann, C. M. Morales, C. R. Landis, F. Weinhold, Theoretical Chemistry Institute, University of Wisconsin, Madison, WI., 2013.

13 Chemcraft version 1.7 see http://www.chemcraftprog.com.

14 C. F. Macrae, I. J. Bruno, J. A. Chisholm, P. R. Edgington, P. McCabe, E. Pidcock, L. Rodriguez-Monge, R. Taylor, J. van de Streek, P. A. Wood, *J. Appl. Crystallogr.* **2008**, *41*, 466-470.

15 L. J. L. Häller, M. J. Page, S. Erhardt, S. A. Macgregor, M. F. Mahon, M. A. Naser, A. Vélez, M. K. Whittlesey, *J. Am. Chem. Soc.* **2010**, *132*, 18408-18416.

16 a) P. Ren, J. W. Ponder, *J. Phys. Chem. B* **2003**, *107*, 5933-5947; b) P. Ren, J. W. Ponder, *J. Comput. Chem.* **2002**, *23*, 1497-1506; c) R. V. Pappu, R. K. Hart, J. W. Ponder, *J. Phys. Chem. B* **1998**, *102*, 9725-9742.

17 D. Beeman, *J. Comput. Phys.* **1976**, *20*, 130-139.

18 H. J. C. Berendsen, J. P. M. Postma, W. F. van Gunsteren, A. DiNola, J. R. Haak, *J. Chem. Phys.* **1984**, *81*, 3684-3690.

19 P. J. Hay, W. R. Wadt, *J. Chem. Phys.* **1985**, *82*, 299-310.
